# High-power lithium–selenium batteries enabled by atomic cobalt electrocatalyst in hollow carbon cathode

**DOI:** 10.1038/s41467-020-18820-y

**Published:** 2020-10-06

**Authors:** Hao Tian, Huajun Tian, Shijian Wang, Shuangming Chen, Fan Zhang, Li Song, Hao Liu, Jian Liu, Guoxiu Wang

**Affiliations:** 1 Faculty of Science, Centre for Clean Energy Technology, School of Mathematical and Physical Sciences, University of Technology Sydney, Broadway, NSW 2007 Australia; 2 State Key Laboratory of Catalysis, Dalian Institute of Chemical Physics, Chinese Academy of Sciences, 457 Zhongshan Road, 116023 Dalian, China; 3 National Synchrotron Radiation Laboratory, CAS Centre for Excellence in Nanoscience, University of Science and Technology of China, 230029 Hefei, Anhui China; 4 DICP-Surrey Joint Centre for Future Materials, Department of Chemical and Process Engineering, and Advanced Technology Institute, University of Surrey, Guilford, Surrey GU2 7XH UK

**Keywords:** Energy, Materials for energy and catalysis, Batteries, Nanoscale materials

## Abstract

Selenium cathodes have attracted considerable attention due to high electronic conductivity and volumetric capacity comparable to sulphur cathodes. However, practical development of lithium-selenium batteries has been hindered by the low selenium reaction activity with lithium, high volume changes and rapid capacity fading caused by the shuttle effect of polyselenides. Recently, single atom catalysts have attracted extensive interests in electrochemical energy conversion and storage because of unique electronic and structural properties, maximum atom-utilization efficiency, and outstanding catalytic performances. In this work, we developed a facile route to synthesize cobalt single atoms/nitrogen-doped hollow porous carbon (Co_SA_-HC). The cobalt single atoms can activate selenium reactivity and immobilize selenium and polyselenides. The as-prepared selenium-carbon (Se@Co_SA_-HC) cathodes deliver a high discharge capacity, a superior rate capability, and excellent cycling stability with a Coulombic efficiency of ~100%. This work could open an avenue for achieving long cycle life and high-power lithium-selenium batteries.

## Abstract (web summary)

Lithium selenium batteries are attractive energy storage systems, but they are hindered by low selenium reaction activity and rapid capacity fading. Herein, the authors report a selenium host with atomic cobalt electrocatalyst which exhibits superior performances in lithium-selenium batteries.

## Introduction

Rechargeable lithium-ion batteries (LIBs) are considered to be the promising candidates towards sustainable energy storage devices due to its long cycle life, high specific power and energy density^1,2^. However, the energy density of current LIBs can not meet the ever-increasing demands from many emerging applications such as electric vehicles^3^. On one hand, lithium–sulfur (Li–S) batteries have attracted growing attention because of several advantages such as the natural abundance of sulfur, high specific energy density (2600 W h kg^−1^) and high theoretical capacity (1675 mA h g^−1^)^4^. However, the development of Li–S batteries still suffers from the inherent issues of low electronic conductivity of sulfur and the shuttle effect of polysulfides. As an element in the same group of sulfur in the periodic table, selenium owns similar chemical properties to sulfur and has been considered as an alternative cathode material for lithium–selenium battery because of its high theoretical volumetric capacity (3253 mA h cm^−3^)^5,6^. Additionally, the conductivity of Se (1 × 10^−3^ S m^−1^) is much higher than that of S (5 × 10^−30^ S m^−1^), which enables higher active material utilization and better rate capability^7^. However, the Se cathodes also have a dissolution issue associated with high-order lithium selenides (Li_2_Se_*x*_, *x* > 4) and large volume expansion during the charge/discharge process, resulting in a low Se utilization, inferior capacity and short cycle life^7–9^.

From the pioneering work by Amine et al.^10,11^, various strategies have been proposed to improve the electrochemical performance of selenium cathode. The most effective method is to incorporate Se particles with electronically conductive materials and encapsulate Se particles within a porous carbon matrix^6,12,13^. For Se/porous carbon composite materials, the charge transfer resistance can be decreased and the shuttle effect of polyselenides can be suppressed because of the high conductivity of carbon matrix and the strong affinity of porous carbon for Se particles^7^. Many porous carbon materials have been studied to construct Se/porous carbon composites for Li–Se batteries, such as carbon nanospheres^14,15^, carbon nanofibers^16^, hierarchical porous carbon^17^ and porous hollow carbon bubbles^18^. However, high-power Li–Se batteries with long cycling performance under high currents have never been reported due to the unsatisfactory performance of Se cathodes.

Single-atom catalysts (SACs) consist of isolated metal atoms dispersed or anchored on matrix materials. SACs attracted extensive attention due to their maximum atom utilization efficiency, homogenous active centres, and unique reaction mechanisms^19–21^. Currently, SACs have been successfully applied in batteries, including metal-air batteries and metal sulfur batteries^22–26^. In addition, the metal-organic framework (MOF)-derived SACs have been intensively investigated in the area of electrocatalysts because of their high electrical conductivity, superior activity, and maximum atomic utilization^27–30^. However, based on literature reviews, as a result of the great challenge of controllable synthesis atomic metals with a selenium host, there has been no report of single atoms in Li–Se batteries, where single atoms can maximize the multi-functions of a selenium host to achieve high rate and cycling performance in a Li–Se battery.

**Fig. 1:**
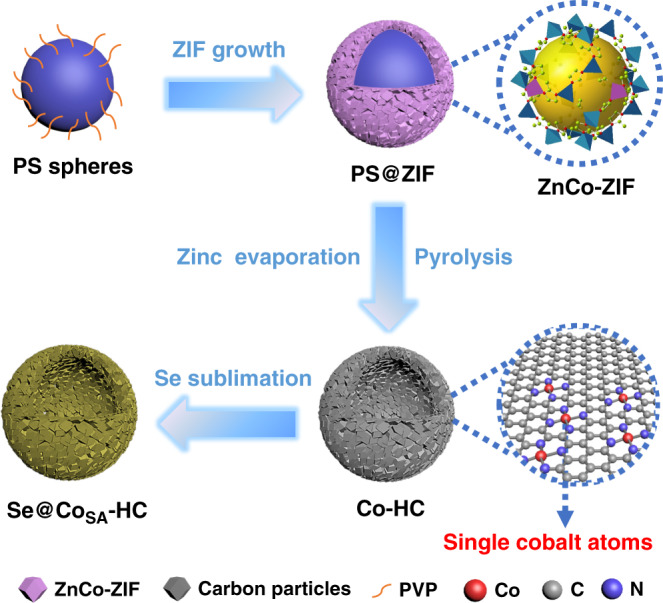
SAC preparation process. Schematic illustration of the procedures for synthesising cobalt single atoms/nitrogen-doped hollow porous carbon (Co_SA_-HC) particles.

Herein, for the first time, we demonstrate that single-atom catalysts can enable highly effective cathodes for Li–Se batteries with superior rate capability and outstanding long-term cycling performance. A facile and straightforward approach (Fig. 1) facilitates the delicate control of Zeolitic Imidazolate Framework (ZIF) particles deposited on the surface of polystyrene (PS) spheres. More importantly, the core–shell ZIF hybrid structure can be further converted into hollow structured carbon materials via a pyrolysis process with zinc evaporation. Through finely tuning the ratio between Zn and Co, we successfully prepared atomic cobalt electrocatalyst/nitrogen-doped hollow porous carbon (Co_SA_-HC), nitrogen-doped hollow porous carbon (HC) and cobalt nanoparticles/nitrogen-doped hollow porous carbon (Co_NP_-HC). In addition, by embedding Se in hollow structured carbon particles, carbon/selenium composites (Se@Co_SA_-HC) was obtained. When applied as cathode materials for Li–Se batteries, the Se@Co_SA_-HC cathode exhibited a superior electrochemical performance, including a superior rate capability (311 mA h g^−1^ at 50 C) and excellent cycling stability (267 mA h g^−1^ after 5000 cycles with a 0.0067% capacity decay per cycle at a current density of 50 C) with the Coulombic efficiency of ~100%. This work reveals that the maximal utilization of cobalt single atoms can optimize the features of porous carbon materials towards the activation of selenium reactivity and immobilization of selenium and polyselenides. Our results demonstrate that the Se@Co_SA_-HC composite is a promising cathode material for lithium–selenium batteries with long cycle life and high-power.

## Results

### Synthesis and characterization

**Fig. 2:**
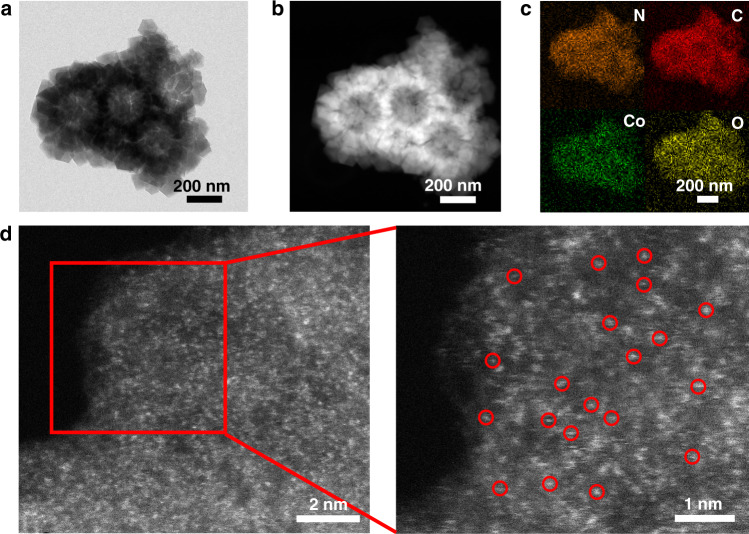
Representative electron microscopy images. **a** TEM image, **b** HAADF image, **c** STEM element mapping images, **d** aberration-corrected HAADF-STEM and magnified images of Co_SA_-HC.

The synthesis strategy for PS@ZIFs is schematically depicted in Fig. 1. After mixing the PVP-modified PS spheres with zinc nitrate, cobalt nitrate, and 2-methyl-imidazolate successively in methanol solution at room temperature, PS@ZIF particles and small ZnCo-ZIFs clusters are formed on the surface of the PS spheres. Three samples with Zn/Co molar ratios of 20:1, 21:0 and 17:4 have been obtained, designated as PS@ZIF-1, PS@ZIF-2, and PS@ZIF-3, respectively. The growth of ZIF particles on the surface of PS spheres is determined by the interfacial reaction between the substrate and surfactant. Polyvinylpyrrolidone (PVP) plays an important role in the homogeneous growth of ZIFs particles on the surface of PS spheres. To improve electrostatic forces and provide enough coordination sites to uniformly adsorb metal ions, the negatively charged PS spheres are firstly enriched with PVP molecules. The amide carbonyl groups of PVP can fully coordinate with metal ions through chemical bonds and make it possible to achieve the coating of ZIFs on the surfaces of the PS spheres^31^. As shown in Supplementary Fig. 1a, the particle size of the prepared PS@ZIF-1 is about 700 nm and the particle size of the polyhedral-structured ZIFs on the surface of PS spheres is ~200 nm. The XRD result for PS@ZIF-1 shown in Supplementary Fig. 2 agrees well with that of as-prepared bimetallic ZnCo-ZIFs^32^, confirming the formation of bimetallic ZIF structures. This growth strategy can be extended to the fabrication of other types of ZIFs with different dimensions and components. The SEM images and XRD patterns in Supplementary Fig. 3 show the successful preparation of rGO@ZIF and MnO_2_@ZIF. The as-prepared PS@ZIF materials were converted into carbonaceous nanocomposites via one-step pyrolysis. The PS template was removed in situ by evaporation to form a hollow morphology at 700 °C under a N_2_ atmosphere. Owing to the composition and size of cobalt in the three products, the obtained carbon materials are denoted Co single atom/nitrogen-doped hollow porous carbon (Co_SA_-HC), nitrogen-doped hollow carbon (HC) and cobalt nanoparticle/nitrogen-doped hollow porous carbon (Co_NP_-HC), respectively. In the previous reports, hollow-structured materials have shown excellent electrochemical performance in energy storage due to large interior voids, high surface area, and shortened mass/charge transport lengths^33–37^. As shown in the SEM image (Supplementary Fig. 4), Co_SA_-HC particles with a decreased particle size of the polyhedral ZIF particle ~130 nm were achieved. TEM images (Fig. 2a) and high angle annular dark-field scanning transmission electron microscopy (HAADF-STEM) images (Fig. 2b) also confirmed the hollow nature of the as-prepared carbon materials and no cobalt aggregates are observed, suggesting that the cobalt nanoparticles are anchored uniformly on the carbon matrix. Energy-dispersive X-ray spectroscopy (EDS) images were obtained to identify the elemental distribution of cobalt, nitrogen, oxygen, and carbon. As shown in Fig. 2c, elements are homogenously distributed in the whole carbon framework. From aberration-corrected HAADF-STEM images in Fig. 2d and Supplementary Fig. 5, high-density bright dots (highlighted by red circles) can be detected, revealing the formation of atomic cobalt. The generation of atomically dispersed Co catalysts was attributed to the Co ions being reduced by carbonized organic linkers with the evaporation of elemental Zn during the calcination process^28,38^. The TEM images in Supplementary Fig. 6 and Supplementary Fig. 7 show that the generation of HC particles and Co_NP_-HC particles from pyrolyzed bimetallic ZnCo-ZIFs with Zn/Co molar ratios of 21:0 and 17:4. It is revealed that the distance of adjacent Co atoms can be expanded through the introduction of zinc species, achieving the controllable synthesis of different aggregation degrees of atomic Co.

X-ray diffraction (XRD) measurement was performed to investigate the phase structure of Co_SA_-HC, HC and Co_NP_-HC particles (Supplementary Fig. 8). The peaks around 26.8° and 44.1° in the XRD pattern of Co_SA_-HC and HC particles fit the (002) and (100) planes of graphitic carbon. After increasing the cobalt content in the Co_NP_-HC particles, newly formed peaks are observed at 44.6°, 51.9° and 76.7°, which can be ascribed to cubic cobalt metal (JCPDS 15-0806). The porosity of the Co_SA_-HC particles was then studied by nitrogen adsorption-desorption analysis. The Co_SA_-HC displays mixed type I and IV isotherms (Supplementary Fig. 9a). The sharp adsorption increase in the low-pressure region indicates that the existence of abundant micropores and the hysteresis loop in the medium-pressure region originates from the mesopores with the carbon framework. The pore size distribution of Co_SA_-HC particles was then analysed based on the isotherms (Supplementary Fig. 9b), which shows peaks centred at 1, 17 and 35 nm. The Brunauer–Emmett–Teller (BET) surface area obtained on the basis of the aforementioned adsorption isotherm of Co_SA_-HC, HC and Co_NP_-HC are 221, 265 and 136 m^2^ g^−1^, respectively. The graphitization degree of a carbon material can be characterized by an *I*_D_/*I*_G_ value (*I*_D_ and *I*_G_ represents the intensity of Raman D- and G- bands of carbon materials) in Raman spectra (Supplementary Fig. 10). The ratios for Co_SA_-HC, HC and Co_NP_-HC were 0.99, 1.00 and 1.01, respectively, indicating the similar graphitic structures in these three hybrids. This allows the exclusion of the influence of carbon supports on electrochemical performance, allowing us to concentrate instead on variances of Co particle size and configuration.

Selenium encapsulation into carbon particles was carried out at 300 °C under Ar atmosphere to promote the infusion of Se into the microporous carbon clusters. The obtained selenium carbon composites are denoted Se@Co_SA_-HC, Se@HC and Se@Co_NP_-HC, respectively. To obtain a high selenium content in the composite materials, selenium powders were mixed with HC particles in a weight ratio of 1:3, respectively. SEM images (Supplementary Fig. 11a) and TEM images (Supplementary Fig. 11b) of the as-formed selenium composite material (Se@Co_SA_-HC) revealed the formation of a size-reduced structure. The HAADF-STEM and EDS images in Supplementary Fig. 11c suggest Se atoms are dispersed uniformly throughout the hollow carbon microstructure. The diffraction peaks of Se in Supplementary Fig. 12 correspond to trigonal crystalline (JCPDS 06-0362) and the XRD pattern of Se@Co_SA_-HC (Supplementary Fig. 12) indicated the successful incorporation of Se in the HC carbon matrix. After the selenization process, the crystallinity of selenium is low, which can be attributed to the transformation of trigonal Se to amorphous Se with the formation of low ordering of selenium and successful confinement in the carbon matrix^7–9^. The Se contents in Se@Co_SA_-HC, Se@HC, Se@Co_NP_-HC and Se@Co_SA_-HC with high Se loading were determined to be 57, 55, 57 and 73 wt%, respectively, through thermogravimetric analysis (TGA) (Supplementary Fig. 13).

### Atomic structure analysis

**Fig. 3:**
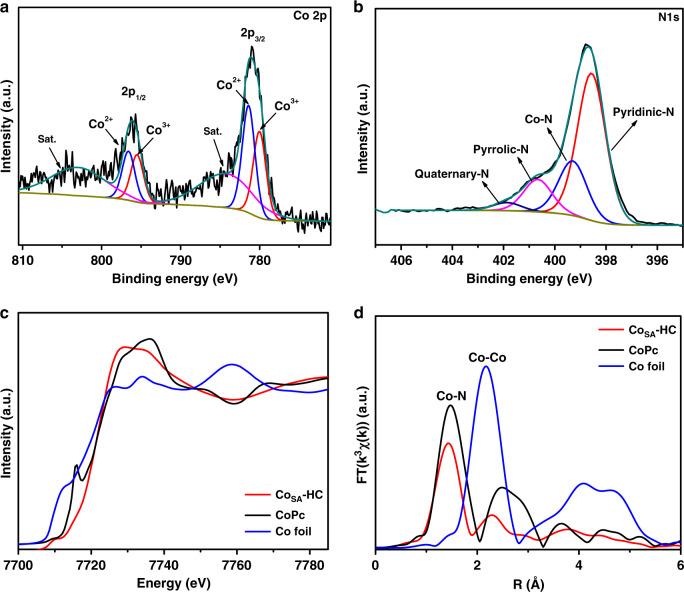
Structure analysis of the Co_SA_-HC particles. **a** Co 2p and **b** N 1 s XPS spectra of Co_SA_-HC particles, **c** Co XANES spectra and **d** Fourier-transform EXAFS spectra of Co_SA_-HC, Co foil and Co phthalocyanine (CoPc).

XPS measurements were also used to explore the chemical environment of Co_SA_-HC particles. The XPS high-resolution C 1 s spectrum of Co_SA_-HC particles is shown in Supplementary Fig. 14 and can be divided into four individual peaks corresponding to C–C (284.8 eV), C–N (286.2 eV), C–O (287.8 eV) and C=O (289.3 eV)^39,40^. In the high-resolution Co 2p spectrum (Fig. 3a), the deconvoluted peaks at 781.4 eV and 796.6 eV can be ascribed to Co 2p_3/2_ and Co 2p_1/2_ orbitals of Co^2+^ species and the peaks at 780.0 eV and 795.5 eV correspond to Co 2p_3/2_ and Co 2p_1/2_ orbitals of Co^3+^ species^28,38^. The peaks at 784.6 and 802.8 eV are satellite peaks that can be ascribed to the shakeup excitation of the high-spin Co^2+^ ions^36^. The N 1 s spectrum in Fig. 3b can be deconvoluted into four peaks located at 398.6, 399.3, 400.7 and 401.8 eV, corresponding to pyridinic N, Co–N, pyrrolic N, and quaternary-N, respectively^41–43^. X-ray absorption near-edge structure (XANES) and extended X-ray absorption fine structure measurements (EXAFS) were conducted to investigate the chemical state and coordination environment of Co atoms in the Co_SA_-HC particles. As shown in the XANES spectra (Fig. 3c), the comparison of near-edge adsorption energy with reference cobalt phthalocyanine (CoPc) and Co foil implies that the Co single atoms in Co_SA_-HC particles are positively charged, which agrees well with previous results^38,44^. Furthermore, from the EXAFS spectra of the three samples (Fig. 3d), a main peak at ~1.48 Å can be observed for CoPc, which is typically assigned to the Co–N tetrahedral coordination (Co–N_4_)^44^. The Co–N coordination peak shifts to a low R-position at 1.43 Å in the HC particles, revealing a slightly variation of Co–N coordination. Compared with the spectra of HC particles and Co foil, no Co-Co peak around 2.1 Å is observed in Co_SA_-HC particles, indicating atomically dispersed Co single atoms^45,46^. Furthermore, according to the fitting parameters given in Supplementary Table 1, the Co–N coordination number of Co_SA_-HC particles is 3.3, implying that the Co–N interaction within HC particles are consisted of Co–N tridentate (Co–N_3_) and tetrahedral (Co–N_4_) coordination. Inductively Coupled Plasma Optical Emission Spectrometry (ICP-OES) analysis of Co_SA_-HC particles indicates that the cobalt content was determined to be ~1.3%.

### Electrochemical performance

**Fig. 4:**
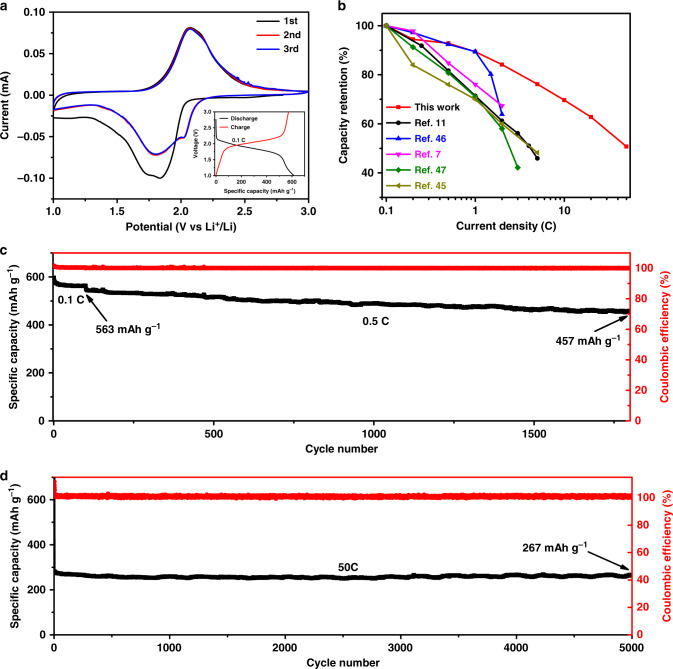
Lithium-ion-storage behaviour of a Se@Co_SA_-HC cathode. **a** CV curves of Se@Co_SA_-HC at a scan rate of 0.1 mV/s (the first three cycles) (inset: discharge-charge voltage profiles of Se@Co_SA_-HC at 0.1 C). **b** The rate capability of Se@Co_SA_-HC compared with other reported Se-based electrodes for Li–Se battery. **c** Cycling performance and Coulombic efficiency at 0.1 C for 100 cycles and then 0.5 C for 1700 cycles. **d** Long cycling performance and Coulombic efficiency at 50 C for 5000 cycles.

Figure 4a reveals the cyclic voltammogram (CV) curves of the first three cycles of a Se@Co_SA_-HC electrodes at a scan rate of 0.1 mV/s between 1.0 V and 3.0 V. During the first discharge process, two peaks at around 1.74 and 1.78 V are observed, which may be related to the lithiation of different Se molecules in the Se@Co_SA_-HC composites^47,48^. However, these two peaks disappear in the second discharge process, simultaneous with the appearance of two reductions peak at around 1.81 and 2.02 V, resulting from the electrochemical activation behaviour of the Se@Co_SA_-HC electrode during the lithiation process^16,49^. During the charge process, there is only one single anodic peak at 2.07 V in all cycles, and this peak remains stable during following lithiation/delithiation cycles. The CV curves after the second cycle are overlapping, demonstrating the good electrochemical stability of the Se@Co_SA_-HC electrodes. As illustrated in Supplementary Fig. 15, the CV curves after the 10th and 100th cycle overlap very well at a higher scan rate of 2.0 mV/s. The inset of Fig. 4a shows the second-cycle galvanostatic discharge-charge voltage profiles of Se@Co_SA_-HC at the current density of 0.1 C. Both discharge and charge processes exhibit stable voltage plateaus at around 2.0 V, which is consistent with the characteristic peaks from the CV curves. The specific capacities of Se@Co_SA_-HC electrodes at different current densities as a function of cycle number (from the second cycle) are presented in Supplementary Fig. 16a. The Se@Co_SA_-HC electrodes exhibit high specific capacities of 613, 579, 569, 548, 516, 467, 427, 385 and 311 mA h g^−1^ at current densities of 0.1, 0.2, 0.5, 1, 2, 5, 10, 20 and 50 C, respectively, which are higher than those of Se@HC and Se@Co_NP_-HC (Supplementary Fig. 16b). When the current rate was returned to 1 C, a high discharge capacity of 537 mA h g^−1^ could still be attained with almost no capacity degradation, suggesting excellent stability of Se@Co_SA_-HC cathodes. Supplementary Figure 17 shows the discharge and charge profiles of Se@Co_SA_-HC, Se@HC and Se@Co_NP_-HC at various rates and cycles. It indicates that both discharge and charge processes exhibit stable voltage plateaus at around 2.0 V. Compared with Se/rGO and Se/MnO_2_ electrodes (Supplementary Fig. 18), Se@Co_SA_-HC cathodes exhibit superior rate electrochemical performances mainly due to the enhanced conductive network, which was previously reported using rGO-based electrodes for Li–Se batteries^50^. Supplementary Figure 19 exhibits the rate capability of an unselenated Co_SA_-HC electrode at different current densities from 0.1 to 5 C. It exhibits a much lower specific capacity of 43 mA h g^−1^ at 0.1 C. This indicates that the capacity contribution from the Co_SA_-HC matrix alone in the Se@Co_SA_-HC composite is negligible. This superior rate performance exceeds those of the reported Se-based materials, especially at high rates (Fig. 4b and Supplementary Table 2)^9,12,49,51,52^.

Figure 4c, d show the cycling stability of the Se@Co_SA_-HC cathode. After 100 cycles at current density 0.1 C (Fig. 4c), the Se@Co_SA_-HC cathodes delivered a high reversible capacity of 563 mA h g^−1^ with 94% capacity retention. Figure 4c further shows the cycling performance of the Se@Co_SA_-HC at a current density of 0.5 C. A large reversible specific capacity of 457 mA h g^–1^ was obtained after 1700 cycles. To highlight the role of atomic Co, the cycling stability of the Se@HC and Se@Co_NP_-HC cathodes were also tested, as presented in Supplementary Fig. 20, which indicates the better capacity retention of Se@Co_SA_-HC. Furthermore, Supplementary Fig. 21 shows the cycling performance of the Se@Co_SA_-HC cathode with a high areal loading of selenium about 5 mg cm^−2^ at 0.2 C for 100 cycles, demonstrating stable cyclability and high capacities. When the selenium mass ratio was increased to 73% in the composite materials, the resultant Se@Co_SA_-HC cathodes with high Se loading delivered 242 mA h g^−1^ at 0.2 C after 100 cycles and 220 mA h g^−1^ at 0.5 C after 100 cycles (Supplementary Fig. 22). As a highlight in Supplementary Fig. 23, the Se@Co_SA_-HC composite cathode operated for 1500 cycles at 5 C with only 0.015% capacity decay per cycle from the 10th to the 1500th cycle, along with a Coulombic efficiency of nearly 100%, indicative of a quite stable prolonged cycle life. Moreover, to further demonstrate the long cycle life of Li–Se battery, the long-term cycle life at a high rate of 20 C (Supplementary Fig. 24) and 50 C (Fig. 4d) was also tested. The Se@Co_SA_-HC electrodes delivered a remarkable capacity of 237 mA h g^−1^ after 2500 cycles with 0.015% capacity decay per cycle at a current density of 20 C and 267 mA h g^−1^ after 5000 cycles with 0.0067% capacity decay per cycle at a current density of 50 C, and both had nearly 100% Coulombic efficiency. To the best of our knowledge, such an exceedingly good cycling stability of Li–Se batteries especially at high current rates such as 50 C for 5000 cycles has not been reported previously^9,12,49,51,52^.

In order to further clarify the electrochemical processes in Se@Co_SA_-HC electrodes after the first discharge-charge cycle at 3.0 V, XPS measurements were carried out. Compared with bare Co_SA_-HC, there is a newly formed peak at 290.5 eV in the C 1 s XPS spectrum for Se@Co_SA_-HC electrodes as shown in Supplementary Fig. 25a. This peak was detected after the first discharge-charge cycle to 3.0 V, indicating the formation of C–Se bonds^7,47,53^. This peak can probably be ascribed to the strong interaction between the Se molecular and the carbon framework or the interaction between Se particles and a carbonyl group^7^. The peaks centred at 55.6 and 56.3 eV in the Se 3d XPS spectrum in Supplementary Fig. 25b are attributed to Se 3d_5/2_ and Se 3d_3/2_, respectively. In addition, the broad peak located at 59.6 eV is ascribed to C–Se bonding^7,47,53^, which is consistent with the results from high-resolution C1s XPS spectra. Only one peak can be deconvoluted from the N 1s spectrum in Supplementary Fig. 25c. This is the peak located at 400.0 eV, which corresponds to pyrrolic N.

**Fig. 5:**
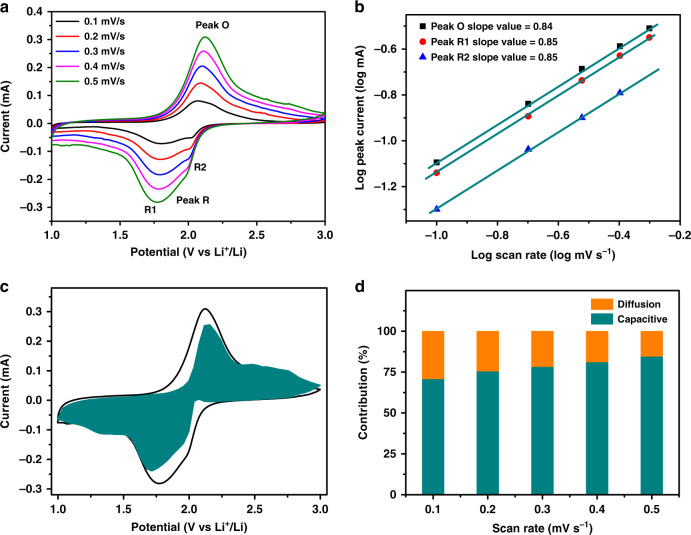
Kinetic analysis of the electrochemical behaviour of a Se@Co_SA_-HC electrode. **a** CV curves of Se@Co_SA_-HC from 0.1 to 0.5 mV/s. **b** The linear fitting plots of the log_10_-transformed peak currents versus scan rates. **c** Contribution of the capacitive process at a scan rate of 0.5 mV s^−1^. **d** Contribution ratio of the capacitive process at different scan rates.

To understand the excellent rate capability of Se@Co_SA_-HC in Li–Se batteries, the electrochemical kinetics were investigated by CV measurements at various scan rates from 0.1 mV/s to 0.5 mV/s. As shown in Fig. 5a, there are two cathodic peaks (denoted as R1 and R2) related to two sequential reactions at 0.1 mV/s. With increasing scan rate to 0.5 mV/s, the peak R1 becomes dominant, while the intensity of the peak R2 is relatively weak. At a scan rate of 0.5 mV/s, the R2 peak in the CV curve almost disappears. To further analyse the difference in kinetics, the current changes with regard to the scan rates could be analysed via as log(*i*) = log(*a*) + *b*log(*v*), where *i* and *v* are the peak current and scan rate, and *a* and *b* are derived parameters^54^. The reaction kinetics can be analysed via the b values. When the *b*-value reaches around 0.5, a diffusion-limited process is occurring during electrochemical reactions. The electrochemical behaviour is an interface-limited process and more capacitive, then *b* value is closer to 1, indicating faster kinetics processes^55^. As shown in Fig. 5b, the fitting curve is almost a linear relationship, and the peak R1 slope value, peak R2 slope value, peak O slope value are calculated to be 0.85, 0.85 and 0.84, respectively. This indicates that the conversion of Se into Li_2_Se is faster than that of Li_2_Se into Se and both conversions are far from being a diffusion-controlled process. Figure 5c reveals that 85% of the total capacity is contributed by a capacitive process at a scan rate of 0.5 mV s^−1^. With an increase in the scan rate, the relative ratio of capacitive contribution to the total capacity gradually increases, as presented in Fig. 5d. It is believed that the capacitive behaviour is closely related to the degree of electrochemical dynamics or kinetics in battery electrodes^56^. Thus, the high ratio of capacitive-controlled contribution in battery electrodes is highly beneficial for fast transport of lithium ions, which would lead to superior electrochemical performance of rate capability and long-term cyclability.

To further evaluate the electrochemical processes and kinetics of Li–Se reactions when using Se@Co_SA_-HC composite cathodes, EIS spectra were collected as a function of the state of discharge/charge. Supplementary Fig. 26a illustrates the typical discharge/charge profile of a Li–Se battery using a Se@Co_SA_-HC composite cathode at 0.1 C. The EIS spectra of the Se@Co_SA_-HC electrode at various depths (marked in Supplementary Fig. 26a) during the discharge/charge process are shown in Supplementary Fig. 26b. All the results exhibit two depressed or overlapping semicircles followed by a sloping line. For Li–Se batteries based on ether-based electrolyte, the first semicircle and second semicircle in EIS spectra for Li–Se batteries could be ascribed to the charge transfer resistance of Se/C electrode and accumulation of interfacial layer on cathode surface, respectively^57^. Combined with the XPS results in Supplementary Fig. 25, it is proposed that a stable layer formed at the surface of the Se@Co_SA_-HC particles during the first lithiation process. Despite the solubility of Se, lithium polyselenides and Li_2_Se in ether-based electrolytes^11^, side reactions have been restricted when using Se@Co_SA_-HC composites and the formed Li_*x*_Se may be protected from further reaction by the stable layer. The equivalent circuit for fitting is shown in the inset of Supplementary Fig. 26c. In the equivalent circuits, R_0_ represents the impedance that is mainly derived from the resistance of the electrolyte, R_1_ is the charge transfer resistance at the conductive agent interface, and R_2_ is the resistance of interfacial layer^57^. CPE1 represents double-layer capacitance (C_dl_), while CPE2 (Constant phase element) describes the space charge capacitance of the layer. W_0_ is the Warburg impedance corresponding to the polyselenide diffusion processes. The resistance values (R_1_ and R_2_) obtained from Supplementary Fig. S26b are summarized in Supplementary Fig. S26c. Supplementary Fig. S27 shows the typical Nyquist plots collected at different discharge-charge state and their fitted curves with two depressed or overlapping semicircles. The obtained resistance values are presented in Supplementary Table 3. The R_1_ value is relatively stable throughout the whole cycle process, indicating the excellent charge transfer capability. The variation of the charge transfer resistance is attributed to the transformation of crown-like Se_8_ to Li_2_Se_x_ during the discharge process, then maintaining amorphous chain-like Se molecules during the charge process. The formation of a stable layer and the reversible Se transformations are responsible for the superior electrochemical performance. The cell with the Se@Co_SA_-HC cathode after 1700 cycles was dissembled and the retrieved Se@Co_SA_-HC cathode materials were characterized by TEM. As shown in Supplementary Fig. 28a, it is observed that the morphology of the Se@Co_SA_-HC cathode has been well preserved, indicating the superior stability of the cathode structure. From STEM element mapping images in Supplementary Fig. 28c, d, energy-dispersive X-ray spectroscopy (EDS) images were obtained to identify the elemental distribution of cobalt and selenium, which are homogeneously distributed in the whole carbon framework. As shown in Supplementary Fig. 28b, a homogeneous thin layer with a thickness of ~20 nm has been identified on the surface of the electrode after long-term cycling. These characterizations confirmed that the composition of the layer could be Li_2_Se_2_/Li_2_Se, which could enhance the cycling performance of the Li–Se batteries. This result is consistent with the previously reported literature^58^. Furthermore, EIS spectra were recorded at open-circuit voltage before cycling and after 1st cycle, 2nd cycle, 5th cycle, 10th cycle and 50th cycle at 0.1 C (Supplementary Fig. 29), 2 C (Supplementary Fig. 30), 5 C (Supplementary Fig. 31) and 20 C (Supplementary Fig. 32). As shown in Supplementary Fig. 29–32, the charge-transfer resistance is very stable after charge/discharge at various current densities. These results further strengthen our claim that Se@Co_SA_-HC materials possess superior rate performance. Furthermore, it is also found that the diameter of the semicircle at 5 C and 20 C decreases with time, which may be attributed to the further conversion reaction with lithium ions and the stabilization of the layer with increasing cycling time^59^.

**Fig. 6:**
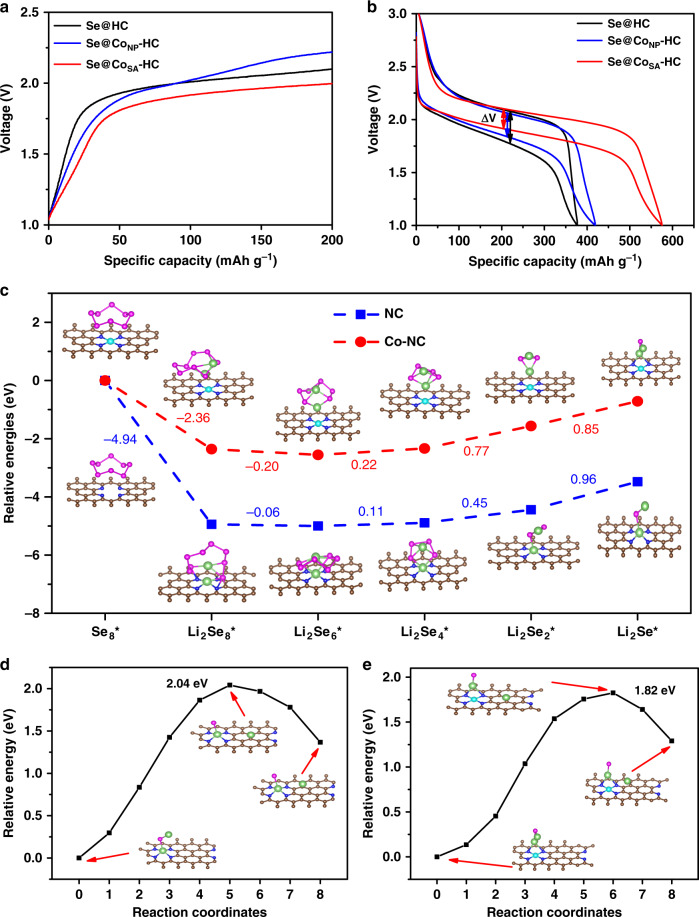
Catalytic effects of Co_SA_-HC particles for Li–Se batteries. **a** The first cycle charge profiles at 0.1 C and **b** discharge-charge profile at 0.1 C of Se@Co_SA_-HC, Se@HC and Se@Co_NP_-HC. **c** Energy profiles for the reduction of lithium polyselenides on NC and Co–NC supports (insets: the optimized adsorption conformations of intermediate species on NC and Co–NC substrate). Energy profiles of the transformation of Li_2_Se clusters on NC (**d**) and Co–NC (**e**). (The insets are: the initial, transition, and final structures, respectively.) The brown, pink, green, blue, and cyan balls represent C, Se, Li, N and Co atoms, respectively.

Owing to the electrocatalytic effect resulting from single cobalt atoms within Co_SA_-HC particles, the Se@Co_SA_-HC electrode shows the lowest overpotentials in charge profiles among the three as-synthesized materials (Fig. 6a). This is in accordance with the smaller value of voltage change ΔV (lowest voltage hysteresis) of test cells by using Se@Co_SA_-HC to produce discharge-charge curves under a current density of 0.1 C compared with Se@HC and Se@Co_NP_-HC, as shown in Fig. 6b. Galvanostatic intermittent titration technique (GITT) testing and EIS spectra were employed to determine the catalyst effect of the single cobalt atoms in Li–Se batteries. The GITT plots of Se@Co_SA_-HC and Se@HC (Supplementary Fig. 33a) and the diffusion coefficients D_Li_^+^ (Supplementary Fig. 33b) of the Se@Co_SA_-HC calculated from GITT plots were estimated to be 1.02 × 10^−13^ ~ 1.7 × 10^−13^ (Supplementary Note 1), which is almost ten times that of the Se@HC cathode (1.03 × 10^−14^ ~ 8.35 × 10^−14^ (Supplementary Note 1)) material, indicating the better electrochemical kinetics for Se@Co_SA_-HC cathode materials in Li–Se batteries. According to the Butler–Volmer equation and EIS spectra (Supplementary Fig. 34), the exchange current density i_0_ of Se@Co_SA_-HC and Se@HC cathode is 1.14 and 0.76 mA cm^−2^ (Supplementary Note 2), respectively. To confirm the catalytic role and stability of single cobalt atoms after long-term cycling, the cell after cycled for 1700 cycles was dissembled and the Se@Co_SA_-HC cathode was retrieved and characterized. From high angle annular dark-field scanning transmission electron microscopy (HAADF-STEM) images in Supplementary Fig. 35, high-density bright dots (highlighted by red circles) have been clearly detected, revealing that single cobalt atoms in the Se@Co_SA_-HC cathode are stable during long-term cycling. All these key kinetic parameters confirm the catalyst effect of the single cobalt atoms.

To further confirm the catalytic activity of single Co atoms after cycling, we conducted a visual observation on the suppression for the formation of lithium polyselenides during the cycling process via single Co atom catalysis. The Se@Co_SA_-HC cathode after cycled for 1700 cycles and the cycled bare Se@HC cathode after cycled 0.5 C for 100 cycles were used for the visual observation. As shown in Supplementary Fig. 36a, H-type cell was used in which the cycled Se@Co_SA_-HC cathode and bare Se@HC cathode were used as the cathode. The electrolyte in the cell with cycled bare Se@HC cathode (Supplementary Fig. 36b, c) changed from colourless to yellow after the first discharge process, implying there was a dissolution of polyselenides in electrolyte^60^. It is clear that by using bare Se@HC cathode without single cobalt catalysts, lithium polyselenides were detached from the cathode and dissolved in the electrolyte, indicating that bare Se@HC cathode owns poor immobilization of polyselenides.

In contrast, the colour change of the electrolyte for the cycled Se@Co_SA_-HC cathode was not observed during three cycles. Supplementary Fig. 36d, e show that the cell cycled Se@Co_SA_-HC cathode presents transparent electrolyte without colour change, indicating the atomic cobalt electrocatalyst could effectively alleviate the dissolution of polyselenides, maximize polyselenides immobilization, electro-catalyse the transformation from polyselenides to Li_2_Se. Therefore, this visual experiment verifies that the single cobalt atoms play an important role that the transformation from polyselenides to Li_2_Se have been electro-catalysed, polyselenides immobilization has been maximized and the dissolution of polyselenides has been inhibited. This visual observation using cycled electrodes clearly confirmed that single atom cobalt catalysts remain active after long cycling.

To further understand the enhancement of reaction kinetics of charge/discharge of the Se@Co_SA_-HC cathodes, first-principles calculations were performed to investigate the different possible reactions of lithium polyselenides on nitrogen-doped carbon support (NC) as a reference and atomic Co/nitrogen-doped carbon supports (Co–NC). As shown in Supplementary Fig. 37, two models of nitrogen-doped carbon without and with Co atoms were considered in our simulation. The reversible overall reaction for the formation of Li_2_Se originating from Se_8_ and Li was considered^12^. During discharge, the first step involves the reduction of Se_8_ and the generation of Li_2_Se_8_, followed by further reduction and disproportionation with the formation of three intermediate lithium polyselenides, namely, Li_2_Se_6_, Li_2_Se_4_, and Li_2_Se_2_, achieving the formation of Li_2_Se as the final product^12^. The Gibbs free energies were calculated for the above reactions on both NC and Co–NC supports (Supplementary Table 4). The optimized structures of the intermediates and their Gibbs free energy profiles are displayed in Fig. 6c. It can be observed that the transformations from Se_8_ to Li_2_Se_6_ are exothermic and the following three steps involving the conversion of Li_2_Se_4_, Li_2_Se_2_, and Li_2_Se are endothermic. The largest positive Gibbs free energy can be found in the conversion process from Li_2_Se_2_ to Li_2_Se, revealing its role as the rate-determining step in the whole discharge process. The Gibbs free energy of Co–NC support (0.85 eV) is much lower than that of NC support (0.96 eV), indicating that the reduction of Se is thermodynamically more favourable on Co–NC than on NC support. In the charging process, the transformation of Li_2_Se is the first step^7^. Through the climbing-image nudged elastic band method, the transformation energy and barrier of Li_2_Se were calculated to evaluate the delithiation reaction kinetics from Li_2_Se to selenium on the surfaces of Co–NC and NC supports. Figure 6d, e show the energy profiles for the transformation processes on both Co–NC and NC supports. The calculated energy barriers of Li_2_Se transformation of Co–NC supports (1.82 eV) is smaller than that of NC (2.04 eV), revealing that atomic cobalt nanoparticles are serving as active sites to enhance the phase transformation of Li_2_Se and the Se utilization in Li–Se batteries.

In addition, from the DFT calculation results shown in Supplementary Table 5, the Li–Se bond length on the surface of Co–NC support is elongated, revealing the interaction between Li atom and the rest of the molecule is weakened. This leads to the easier delithiation for lithium polyselenides, confirming the role of single Co atom catalysts. Therefore, we proposed a mechanism that single Co atoms can quickly catalyse the transformation from Li_2_Se_2_ into Li_2_Se during the discharging process and the transformation of Li_2_Se during the charging process. To demonstrate the mechanism, the schematic illustration of electrode reaction mechanisms for the Se@Co_SA_-HC cathodes is shown in Supplementary Fig. 38. The confined polyselenides within the carbon framework could be fully reduced into Li_2_Se by single atom Co catalysts, leading to high Se utilization. Therefore, the atomic cobalt on the Co–NC support could effectively alleviate the dissolution of polyselenides, electro-catalyse the transformation from polyselenides to Li_2_Se and minimize the reaction energy barriers, leading to the full utilization of selenium, superior cycling ability, and reversible capability.

## Discussion

Until now, single-atom catalysts were achieved through various strategies, including wet impregnation and coprecipitation methods^61^, atomic layer deposition^62^, pyrolysis^63^ and photodeposition^64^. Among them, pyrolysis is a facile method to construct single-atom catalysts through thermal decomposition of suitable precursors. Herein, we have developed a simple synthesis method to achieve hollow structured particles with isolated, positively charged and highly dispersed single Co atoms through tuning the cobalt and zinc contents from bimetallic ZnCo-ZIFs precursors. The aberration-corrected HAADF-STEM image and EXAFS data provide strong evidence that single Co atoms are high dispersed within the Co_SA_-HC particles. It is also revealed from XANES results that the single Co atoms are positively charged and the EXAFS data show that isolated single Co atoms can be atomically anchored into the carbon frameworks through the formation of Co–N_3_ and Co–N_4_ coordination moieties within Co_SA_-HC particles. By using Co_SA_-HC particles, more accessible storage sites and larger electrode/electrolyte contact area are provided, and mass/charge transportation lengths are shortened through the formation of the hollow structure. The volume expansion during lithiation can be inhibited by the large internal void spaces.

Based on the density functional theory (DFT) calculations, it indicates that the reaction rate from the reduction of Li_2_Se_2_ into Li_2_Se is noticeably increased during the discharge process by using a single Co atom catalysts. From the charging process data, a smaller value than the calculated energy barriers for Li_2_Se transformation through the single Co atom catalysts can be obtained. It is proposed that the mechanism of single Co atom enhancement of Li–Se batteries is that single Co atoms can quickly catalyse the transformation from Li_2_Se_2_ into Li_2_Se during the discharging process and the transformation of Li_2_Se during the charging process. Therefore, the atomic cobalt plays the key role in the alleviation of polyselenide dissolution, maximation of polyselenides immobilization and activation via strong electrocatalytic behaviour, achieving the best cycling performance in the field of Li–Se batteries. Therefore, the Se@Co_SA_-HC composite is a promising candidate for long cycle life and high-power lithium–selenium batteries.

In summary, we developed a facile approach for synthesizing core–shell structured PS@ZIF materials which can be further converted into atomic Co electrocatalyst/nitrogen-doped hollow porous carbon (Co_SA_-HC) particles through one-step pyrolysis. To highlight the importance of Co_SA_-HC particles for energy applications, Co_SA_-HC particles were used for Li–Se batteries. More specifically, Se@Co_SA_-HC cathodes delivered an excellent discharge capacity of 564 mA h g^−1^ after 100 cycles at a current density of 0.1 C and a superior rate capability of 385 mA h g^−1^ and 311 mA h g^−1^ at a current density of 20 C and 50 C. In addition, they show a superior reversible capacity of 457 mA h g^−1^ at a current density of 0.5 C after 1700 cycles with only 0.011% capacity decline per cycle and 267 mA h g^−1^ after 5000 cycles at 50 C with a 0.0067% capacity decay per cycle with Coulombic efficiency nearly 100%. These distinctive features of the HC particles are concluded as follows: (i) the atomic cobalt electrocatalyst could effectively alleviate the dissolution of polyselenides, electro-catalyse the transformation from polyselenides to Li_2_Se and minimize the adsorption energy barriers; (ii) the hollow structures provide more accessible selenium storage sites, larger electrode/electrolyte contact area, shortened mass/charge transport lengths; (iii) the large internal void spaces accommodate volume expansion during lithiation; (iv) the conductive carbon materials enhance the electrode conductivity and effectively confine the soluble polyselenides. This work provides an efficient route for the preparation of single-atom materials and paves a new strategy for developing high-power electrochemical energy storage devices.

## Methods

### Materials

The following provides information on chemicals used in this work: methanol (99%), styrene, potassium persulfate (KPS), polyvinylpyrrolidone (PVP, MW~50,000), ethanol (95–100%), zinc nitrate hexahydrate (Zn(NO_3_)_2_·6H_2_O), cobalt nitrate hexahydrate (Co(NO_3_)_2_·6H_2_O), 2-methylimidazole were purchased from Sigma-Aldrich and used as received without any further purification. Washing was achieved with ultrapure water and reagent grade ethanol where required. Ultrapure water was used for solution preparations.

### Synthesis of polystyrene spheres (PS)

The styrene was firstly treated by passing through a column containing neutral alumina to remove polymerization inhibitor and then subjected to vacuum distillation to obtain a purified styrene monomer. 1.1 g of PVP was dissolved in 100 mL deionized water, and then 13 mL of the above treated styrene was added to the solution. After stirring for 30 mins, an aqueous 20 mL solution containing 0.3 g of potassium persulfate (KPS) was then added to the above mixture. Then the mixture was heated to 70 °C and remained at this temperature for 24 h.

### Synthesis of PS@ZIF

In a typical procedure, 0.15 g of as-prepared PS spheres were fully ground and then dispersed into 90 mL of methanol containing 1 g of PVP. After ultrasonic dispersion and vigorous agitation for 3 h, 2.125 g of Zn(NO_3_)_2_·6H_2_O and 0.104 g of Co(NO_3_)_2_·6H_2_O (the molar ratio of Zn^2+^/Co^2+^ was 20 were subsequently dissolved into the mixed solution and stirred for another 0.5 h. Then, 4.926 g of 2-methylimidazole dissolved in 90 mL of methanol was quickly added into the above solution followed by vigorous stirring for 4 h. Finally, the PS@ZIF-1 precursor were collected by centrifugation, washed with methanol for several times, and dried at 60 °C overnight. Similarly, for the synthesis of PS@ZIF-2 and PS@ZIF-3, the procedures were carried out except that the Zn/Co molar ratio was 1:0 and 17:4, respectively.

### Synthesis of Co_SA_-HC, HC, Co_NP_-HC particles

PS@ZIF particles (PS@ZIF-1, PS@ZIF-2 and PS@ZIF-3) were carbonized in flowing N_2_ in a tube furnace using a heating rate of 5 °C min^−1^ up to 700 °C and dwelling for 5 h. After naturally cooling down to room temperature, the products were collected and marked as Co_SA_-HC, HC and Co_NP_-HC particles according to the Zn/Co molar ratio in the precursors, respectively.

### Synthesis of selenium carbon composites

Se powder (99.99%, Sigma-Aldrich) and the as-prepared Co_SA_-HC, HC, Co_NP_-HC particles with a weight ratio of 1:1 were mixed. Subsequently, the mixture was heated at 300 °C for 12 h with heating rate of 5 °C min^−1^ in a tubular furnace under argon atmosphere to achieve selenium carbon composites. The as-prepared materials were named Se@Co_SA_-HC, Se@HC and Se@Co_NP_-HC based on the different carbon precursors.

To achieve high selenium mass ratio in selenium carbon composite, Se powder and the as-prepared Co_SA_-HC particles with a weight ratio of 3:1 were mixed, followed by the same heat treatment as described above.

### Synthesis procedures of rGO@ZIF and MnO_2_@ZIFs

Synthesis of MnO_2_ nanowires: in a typical synthesis, 50 mg of polyvinylpyrrolidone (PVP) and 40 mL of 0.015 M KMnO_4_ aqueous solution were mixed with magnetic stirring, and then the mixture was transferred into a 50 mL Teflon-lined stainless autoclave. The autoclave was sealed and put in an electronic oven at 160 °C for 9 h and then naturally cooled down to room temperature. The precipitates were collected by filtration, washed with deionized water and absolute ethanol several times before drying at 60 °C overnight.

*Synthesis of reduced graphene oxide (rGO)*: Graphene oxide (3 mg/ml) solution were dried via lyophilization and then was ground into powder. Reduced graphene oxide can be obtained through hydrothermal treatment of graphene oxide (0.4 g) with 400 µL hydrazine hydrate at 95 °C for 24 h.

*Synthesis of the MnO*_*2*_*@ZIF*: in a typical procedure, 0.07 g of as-prepared MnO_2_ nanowires were fully ground and then dispersed into 90 mL of methanol containing 1 g of PVP (K-30). After ultrasonic dispersion and vigorous agitation for 3 h, 2.125 g of Zn(NO_3_)_2_·6H_2_O and 0.104 g of Co(NO_3_)_2_·6H_2_O (the molar ratio of Zn^2+^/Co^2+^ was 20 were subsequently dissolved into the mixed solution and stirred for another 0.5 h. Then, 4.926 g of 2-methylimidazole dissolved in 90 mL of methanol was quickly added into the above solution followed by vigorous stirring for 4 h. Finally, the MnO_2_@ZIF precursors were collected by centrifugation, washed with methanol for several times, and dried at 60 °C overnight.

*Synthesis of rGO@ZIF*: in a typical procedure, 0.07 g of as-prepared rGO were fully ground and then dispersed into 90 mL of methanol containing 1 g of PVP (K-30). After ultrasonic dispersion and vigorous agitation for 3 h, 2.125 g of Zn(NO_3_)_2_·6H_2_O and 0.104 g of Co(NO_3_)_2_·6H_2_O (the molar ratio of Zn^2+^/Co^2+^ was 20 were subsequently dissolved into the mixed solution and stirred for another 0.5 h. Then, 4.926 g of 2-methylimidazole dissolved in 90 mL of methanol was quickly added into the above solution followed by vigorous stirring for 4 h. Finally, the rGO@ZIF precursors were collected by centrifugation, washed with methanol for several times, and dried at 60 °C overnight.

### Electrochemical measurements

Electrodes were prepared by mixing the selenium carbon composite, carbon black and polyvinylidene fluoride at a weight ratio of 8:1:1 in N-methyl-2-pyrrolidone solvent, The slurry was pasted onto aluminium foil and dried in a vacuum oven at 60 °C for 12 h. CR2032 coin cells were assembled in an argon-filled glove box (Mbraun, Unilab, Germany) and then used for electrochemical evaluation. The electrolyte contained 1 wt% lithium nitrate (LiNO_3_) in 1,3-dioxolane and 1,2-dimethoxyethane (volume ratio 1:1). Porous polypropylene (Celgard 2300^TM^) was used for the separator membranes. About 20 μL electrolyte was added for each coin cell. The areal loading of selenium in the cathode is 0.8 mg cm^−2^. The cells were discharged and charged galvanostatically in the fixed voltage range 1.00–3.00 V with current rates of 0.1 –50 C rate using a NEWARE battery tester. Cyclic voltammetry (CV) and electrochemical impedance spectroscopy (EIS) were performed on a Biologic VMP3 electrochemical station. CV responses were also tested in voltage range 1.00 V to 3.00 V (vs. Li^+^/Li) at scan rate from 0.1 to 0.5 mV s^−1^. EIS (100 kHz to 10 mHz with an amplitude of 5 mV). Analysis was then applied to evaluate electrochemical behaviors of the electrode.

### Materials characterization

The morphology and chemical composition of the as-prepared samples were observed by field emission scanning electron microscopy (FESEM, Zeiss Supra 55VP), transmission electron microscopy (TEM, Tecnai G2 F30 S-TWIN). X-ray diffraction (XRD) measurements were carried out by using a scanning step of 0.04° per second in the 2θ range from 10° to 80° (Bruker D8 Discovery XRD). X-ray photoelectron spectroscopy (XPS) measurements were performed on an ESCALAB250Xi (Thermo Scientific, UK) equipped with mono-chromated Al K alpha (energy 1486.68 eV). The BET specific surface area and single-point pore volume were obtained from nitrogen adsorption isotherms measured at −196 °C using a nitrogen sorption instrument (ASAP 2460 Micropore Physisorption Analyzer). Prior to nitrogen adsorption measurements, the samples were degassed at 250 °C overnight. Raman spectra were obtained from a Renishaw inVia Raman spectrometer system (Gloucestershire, UK) equipped with a Leica DMLB microscope (Wetzlar, Germany) and a Renishaw He-Ne laser source producing 17 mW at 633 nm. Thermogravimetric analysis (TGA) was performed with a 2960 SDT system. X-ray absorption fine structure (XAFS) measurements of the Co K-edge were performed at the 1W1B beamline of the Beijing Synchrotron Radiation Facility (BSRF) in transmission mode. The X-ray was monochromatized using a double-crystal Si (111) monochromator and the energy was calibrated by a cobalt metal foil for Co K-edge. Cobalt foil and CoPc were used as the reference substance. The XAFS data was analysed using the WinXAS3.1 program. Cobalt content in HC particles was determined by Inductively Coupled Plasma Optical Emission Spectrometry (ICP-OES).

### Computational method and models

All calculations were carried out by using the projector augmented wave method in the framework of DFT^65^, as implemented in the Vienna *ab*-initio Simulation Package (VASP). The generalized gradient approximation (GGA) and Perdew–Burke–Ernzerhof (PBE) exchange functional^65^ were used. Structural relaxation calculations were performed by using the spin-polarized GGA method^66^. A supercell of graphene containing 4 × 2$\sqrt 3$ unit cells was used to model N-G and Co–N-G systems. After convergence tests, the plane-wave energy cutoff was set to 500 eV, and the Monkhorst–Pack method^67^ with 2 × 3 × 1 supercells was employed for the Brillouin zone sampling of N-G and Co−N-G systems. The convergence criterions of energy and force calculations were set to 10^−5^ eV/atom and 0.01 eV Å^−1^, respectively. The Gibbs free energies for electrochemical reduction of molecular Se_8_ and polyselenide Li_2_Se_n_ on the N-G and Co–N-G systems were calculated by the DFT energy difference between two different reduction steps^68,69^. The DFT-D2 empirical correction method was employed to describe van der Waals interactions. The barriers for Li_2_Se transformation on N/G and Co–N/G were calculated with the nudged elastic band (NEB) method to evaluate its delithiation reaction kinetics.

## Supplementary information

Supplementary Information

Peer Review File

## Data Availability

The data that support the findings of this work are available from the corresponding author upon reasonable request.
