# Author Correction: High-power lithium–selenium batteries enabled by atomic cobalt electrocatalyst in hollow carbon cathode

**DOI:** 10.1038/s41467-020-19525-y

**Published:** 2020-10-28

**Authors:** Hao Tian, Huajun Tian, Shijian Wang, Shuangming Chen, Fan Zhang, Li Song, Hao Liu, Jian Liu, Guoxiu Wang

**Affiliations:** 1 Faculty of Science, Centre for Clean Energy Technology, School of Mathematical and Physical Sciences, University of Technology Sydney, Broadway, NSW 2007 Australia; 2 State Key Laboratory of Catalysis, Dalian Institute of Chemical Physics, Chinese Academy of Sciences, 457 Zhongshan Road, 116023 Dalian, China; 3 National Synchrotron Radiation Laboratory, CAS Centre for Excellence in Nanoscience, University of Science and Technology of China, 230029 Hefei, Anhui China; 4 DICP-Surrey Joint Centre for Future Materials, Department of Chemical and Process Engineering, and Advanced Technology Institute, University of Surrey, Guilford, Surrey GU2 7XH UK

**Keywords:** Energy, Materials for energy and catalysis, Batteries, Nanoscale materials

Correction to: *Nature Communications* 10.1038/s41467-020-18820-y, published online 6 October 2020.

The original version of this Article omitted Hao Tian and Huajun Tian as equally contributing authors.

This has now been corrected in both the PDF and HTML versions of the Article.

