# Supplementary material for: High-power lithium–selenium batteries enabled by atomic cobalt electrocatalyst in hollow carbon cathode
Source: Nat Commun. 2020 Oct 6;11:5025. doi: 10.1038/s41467-020-18820-y (PMC7538427; doi:10.1038/s41467-020-18820-y)
Supplement: Supplementary file 1 — Supplementary Information [file 41467_2020_18820_MOESM1_ESM.pdf]

Supplementary Information

High-power lithium-selenium batteries enabled by atomic cobalt electrocatalyst in hollow carbon cathode

Tian et al.

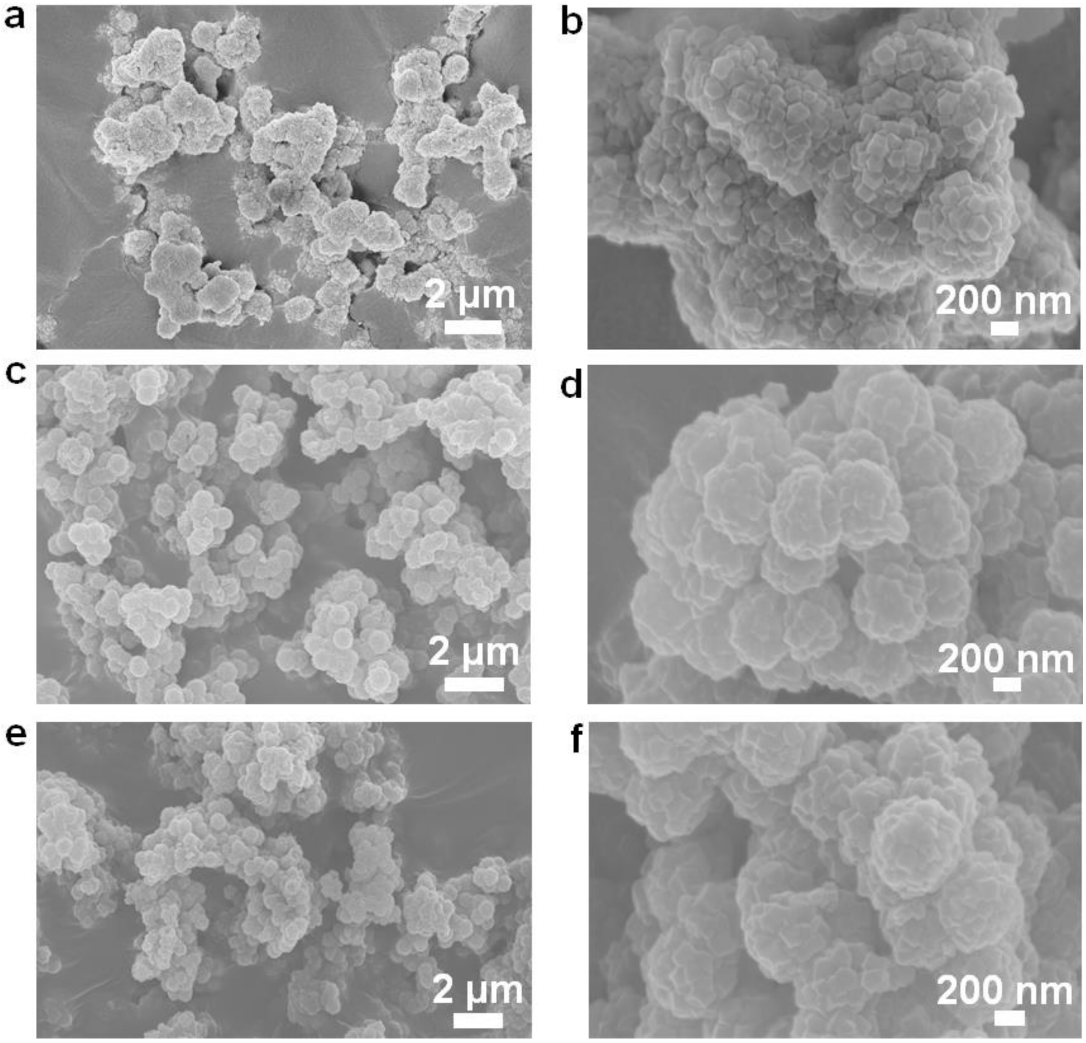

Supplementary Figure 1. (a) (c) (e) Low-magnification SEM image and (b) (d) (f) high-magnification SEM image of PS@ZIF-1, PS@ZIF-2 and PS@ZIF-3.

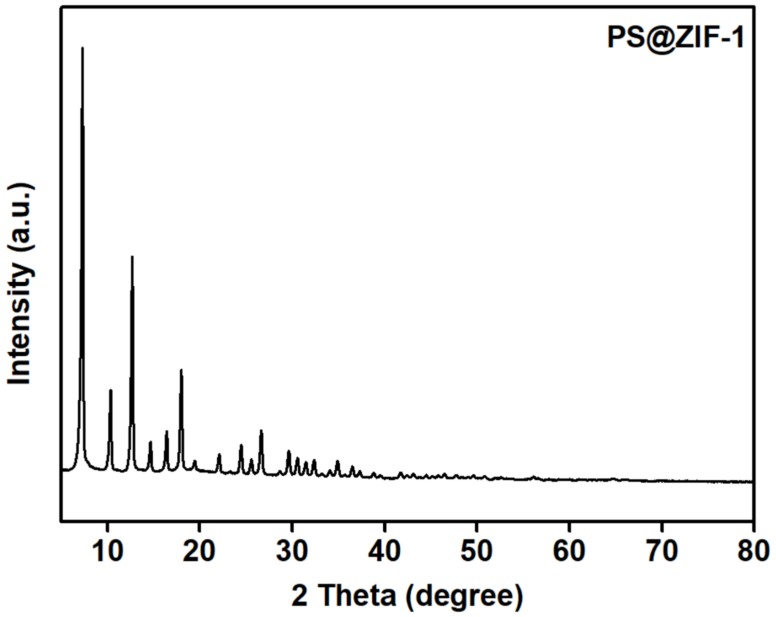

Supplementary Figure 2. XRD pattern of PS@ZIF-1.

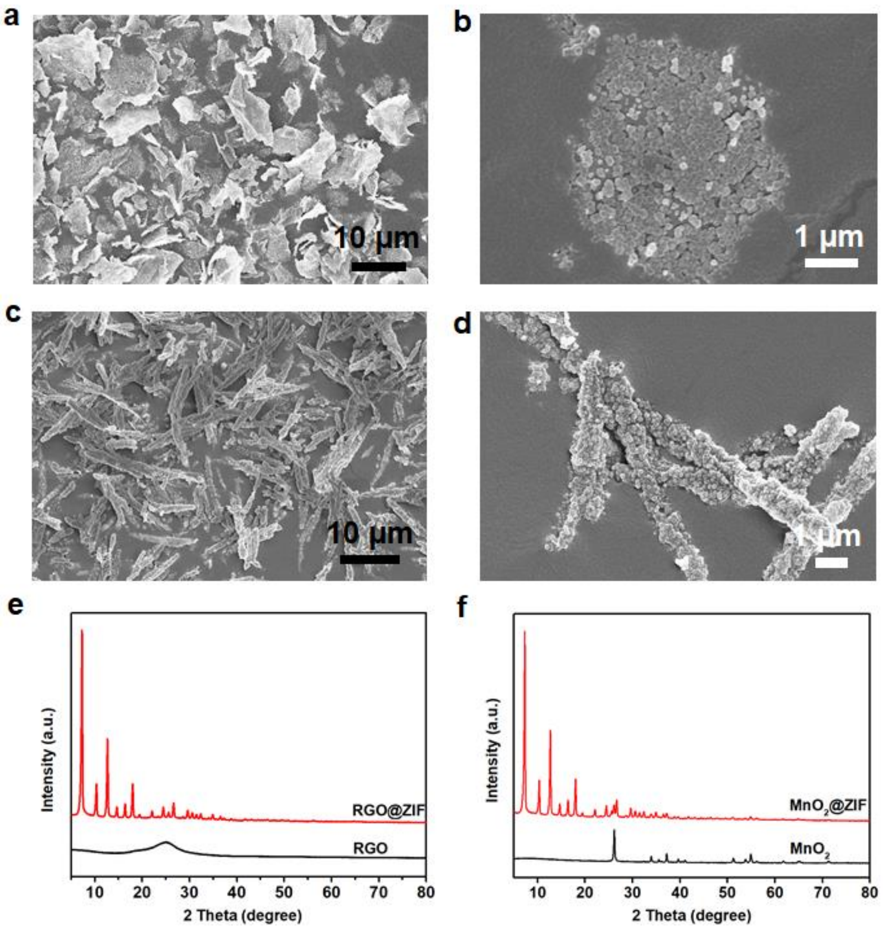

Supplementary Figure 3. Low-magnification (a) and high-magnification (b) SEM image of rGO@ZIF; Low-magnification (c) and high-magnification (d) SEM image of MnO_2@ZIF; (e) XRD pattern of rGO and rGO@ZIF; (f) XRD pattern of MnO_2 and MnO_2@ZIF.

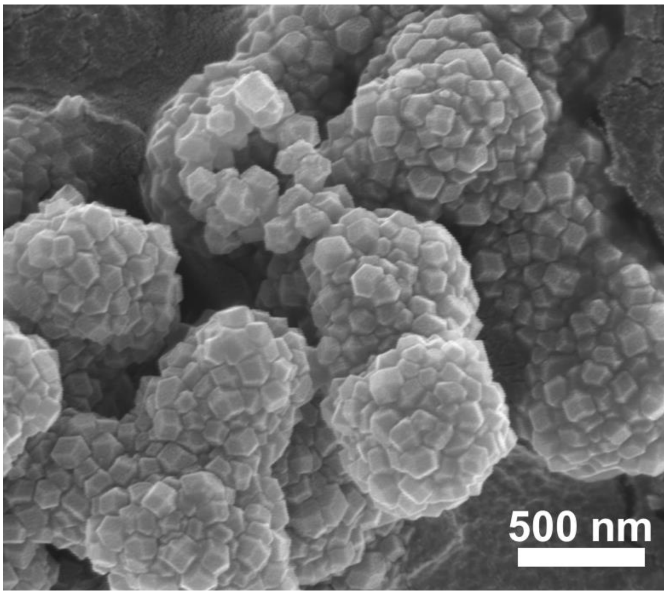

Supplementary Figure 4. SEM image of the Co_{SA}-HC.

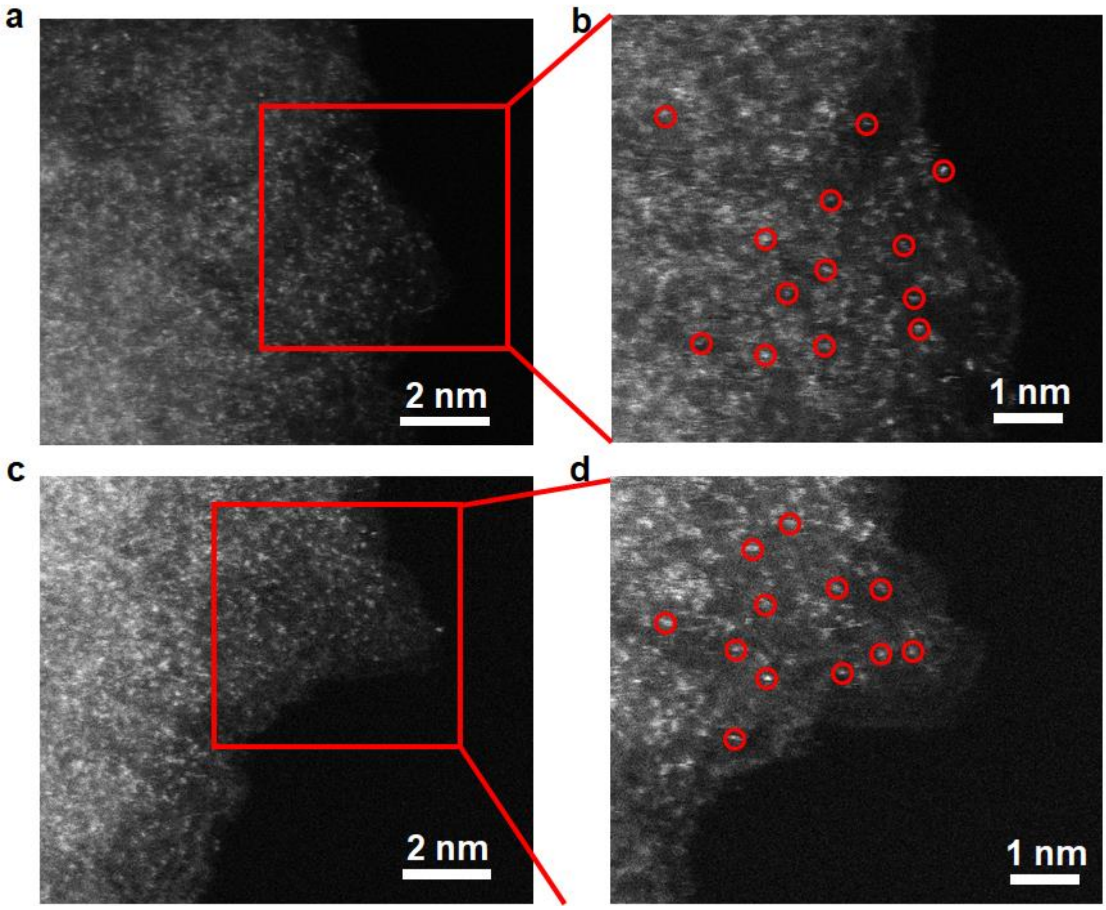

Supplementary Figure 5. (a, c) aberration-corrected HAADF-STEM images and (b, d) enlarged images of the Co_{SA}-HC.

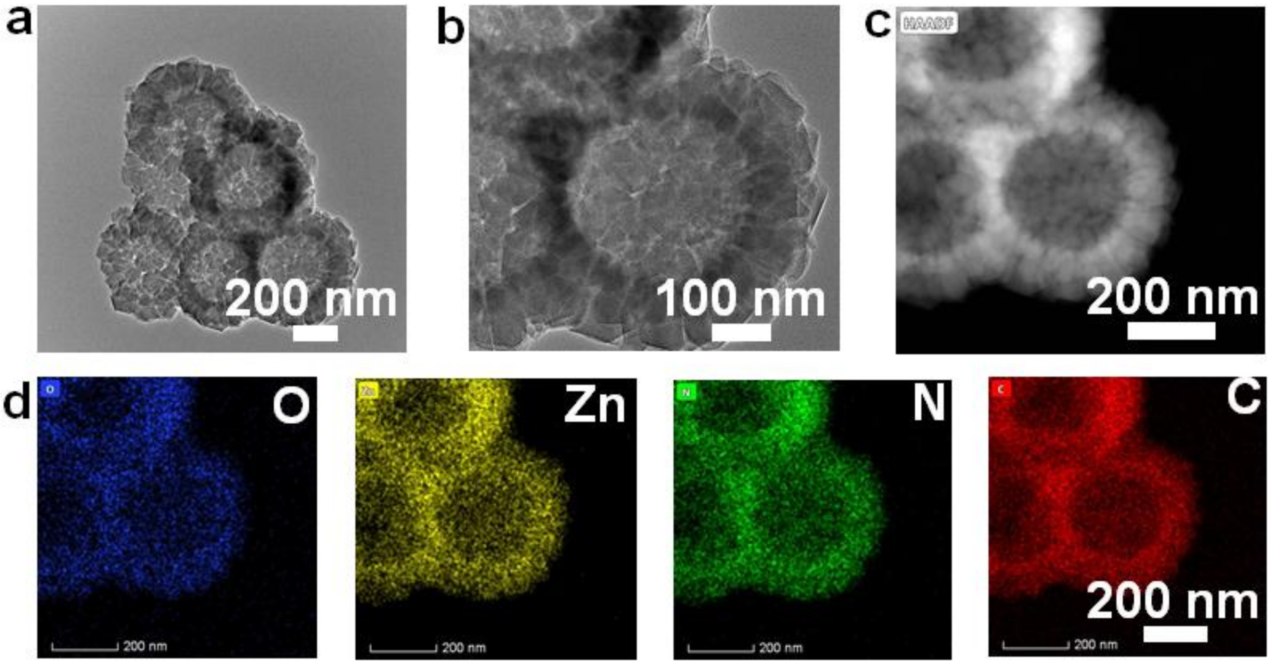

Supplementary Figure 6. TEM and EDS mapping images of nitrogen-doped hollow porous carbon (HC).

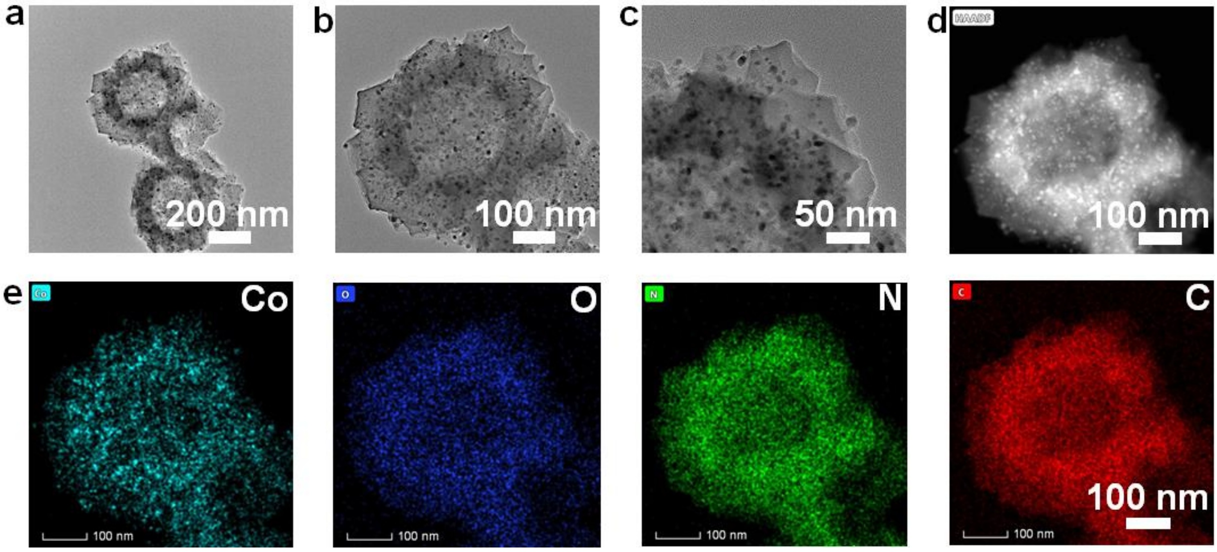

Supplementary Figure 7. TEM and EDS mapping images of cobalt nanoparticles/nitrogen-doped hollow porous carbon (Co_{NP}-HC).

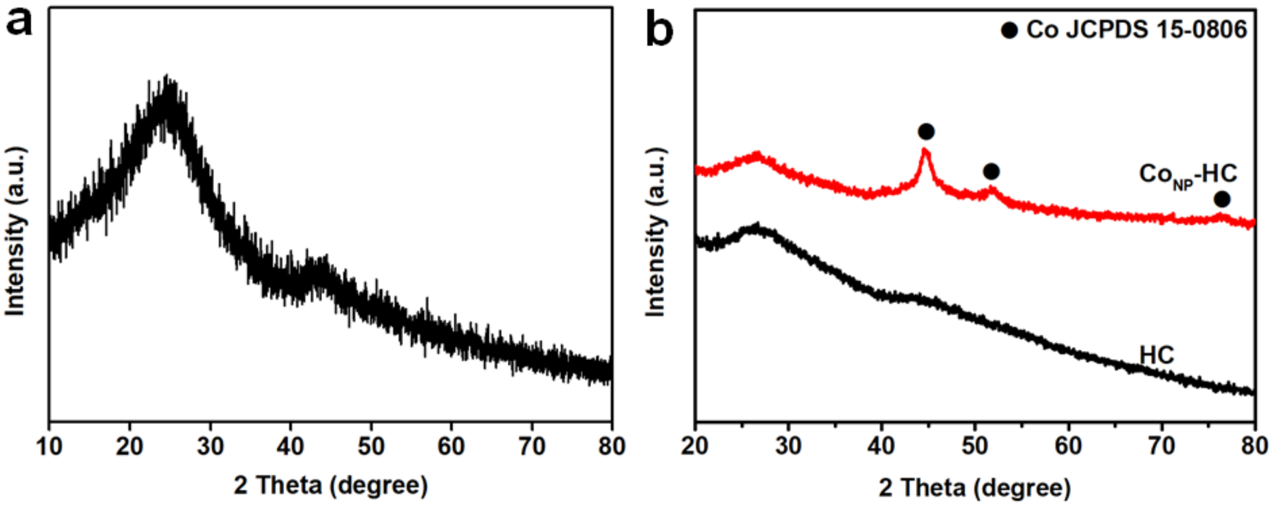

Supplementary Figure 8. XRD patterns of Co_{SA}-HC (a), HC and Co_{NP}-HC (b).

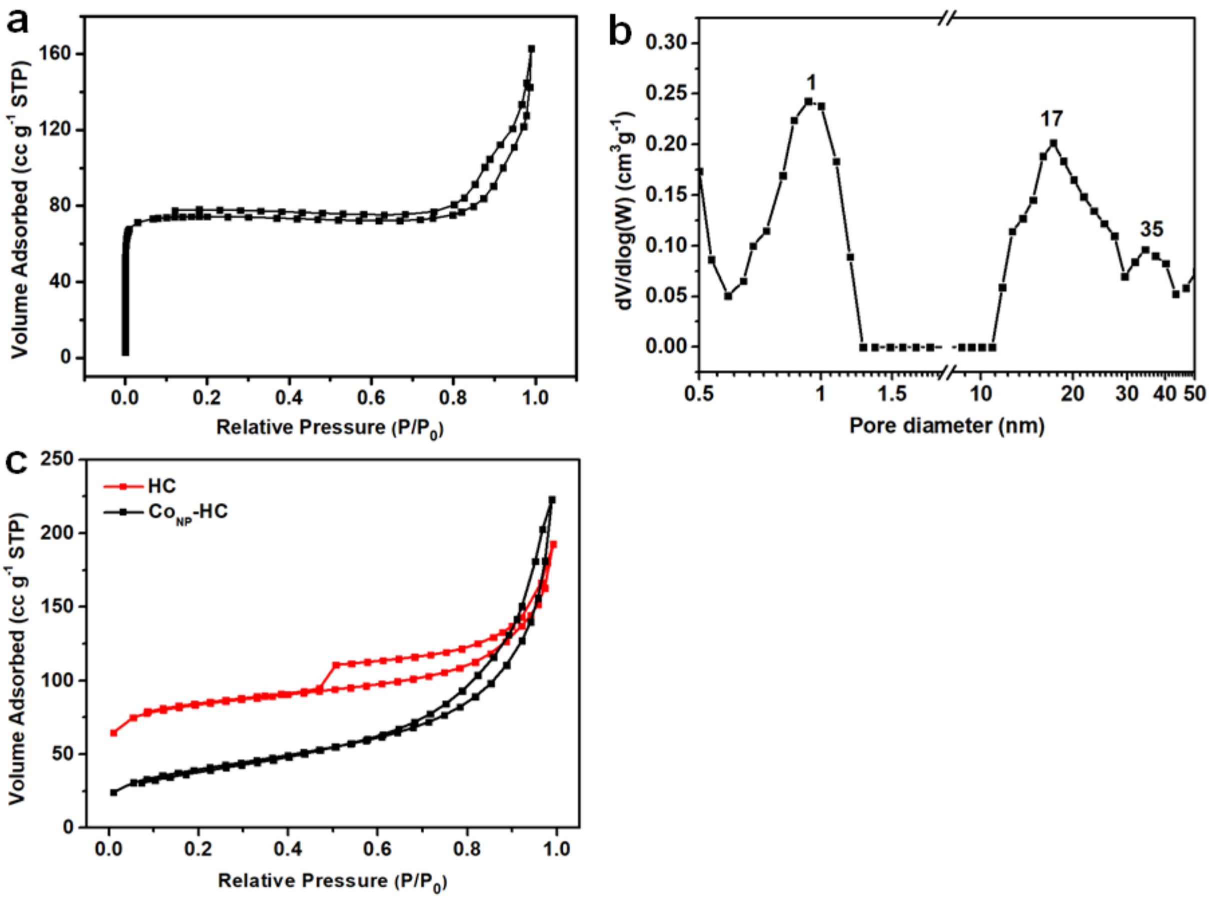

Supplementary Figure 9. N_2 adsorption–desorption isotherms (a) and the corresponding pore size distribution (b) of Co_{SA}-HC particles; (c) N_2 adsorption–desorption isotherms of HC and Co_{NP}-HC particles.

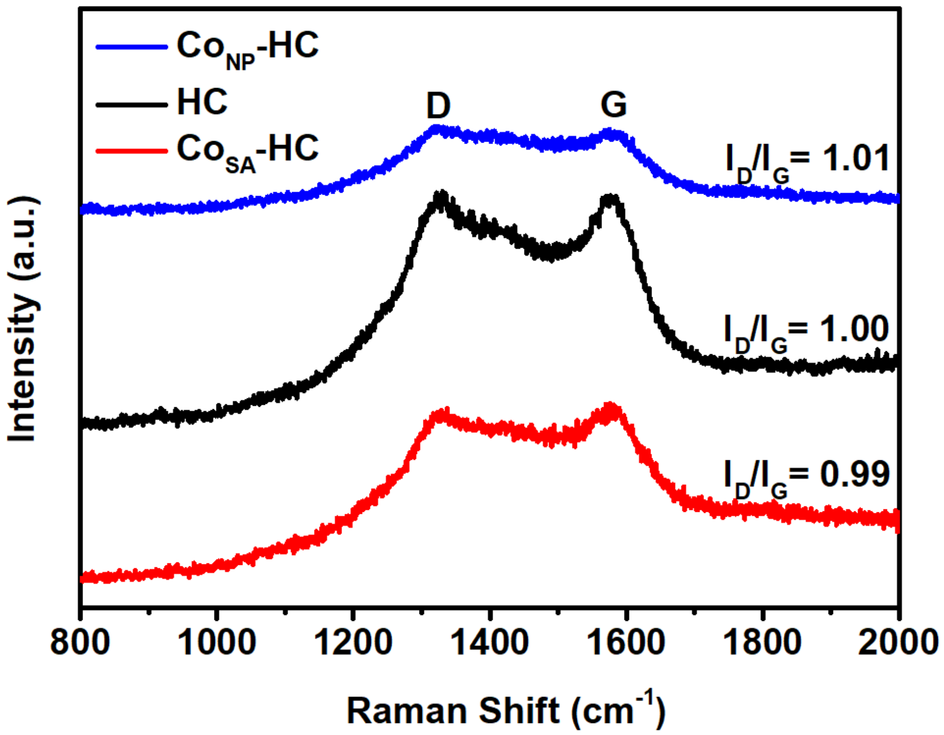

Supplementary Figure 10. Raman spectra of Co$_{SA}$-HC, HC and Co$_{NP}$-HC.

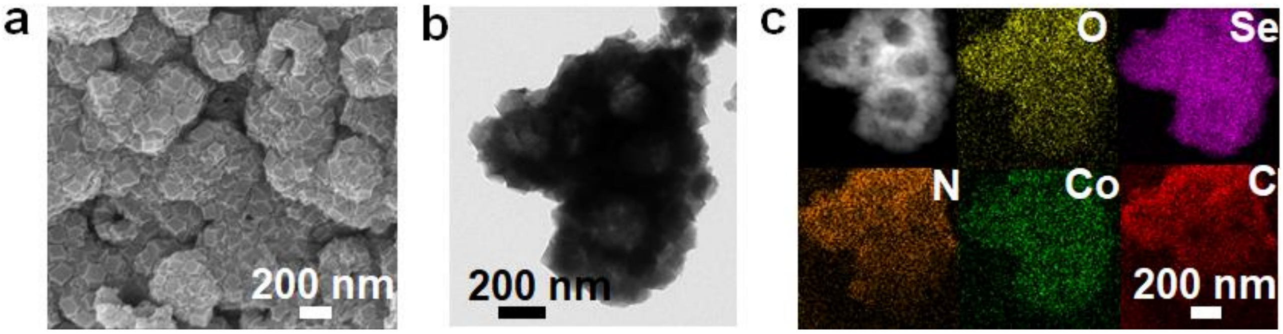

Supplementary Figure 11. (a) SEM image, (b) TEM image and (c) HAADF image and STEM element mapping images of Se@Co_{SA}-HC.

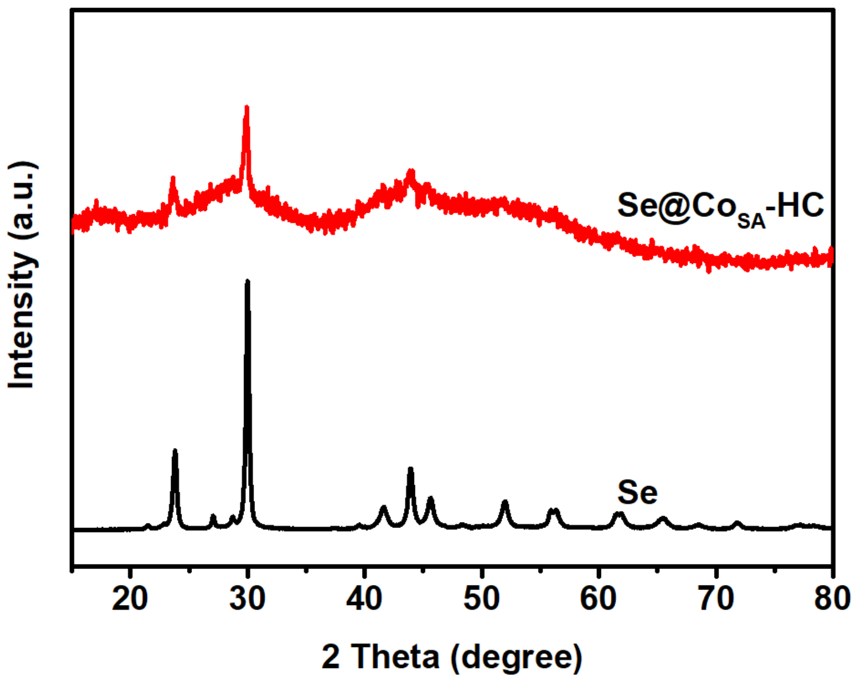

Supplementary Figure 12. XRD patterns of Se and Se@Co$_{SA}$-HC.

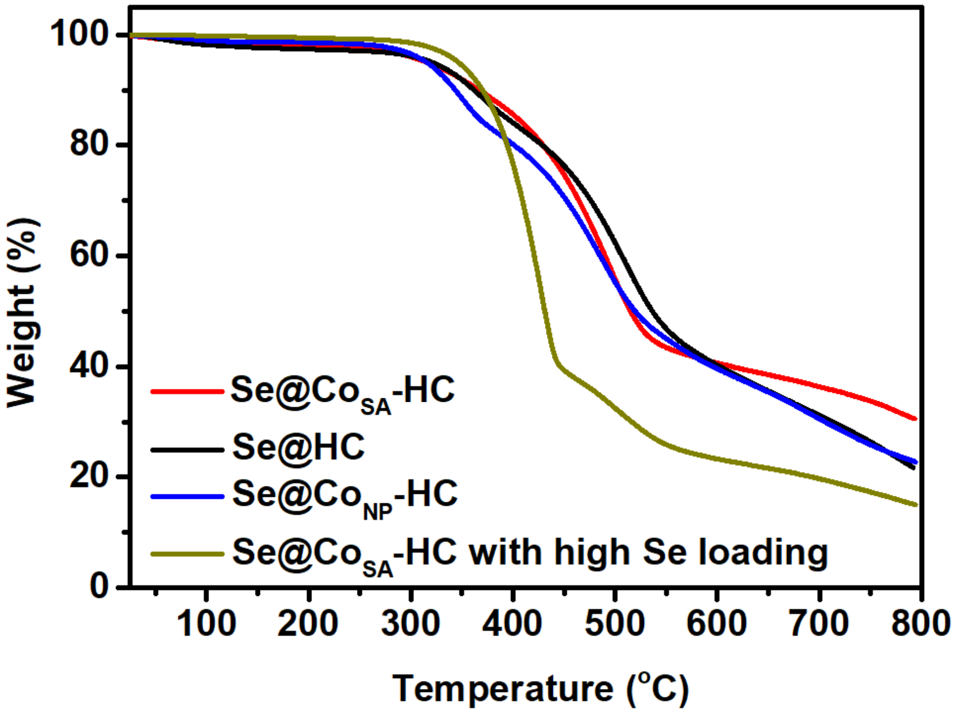

Supplementary Figure 13. TGA curves of Se@Co_{SA}-HC, Se@HC, Se@Co_{NP}-HC and Se@Co_{SA}-HC with high Se loading.

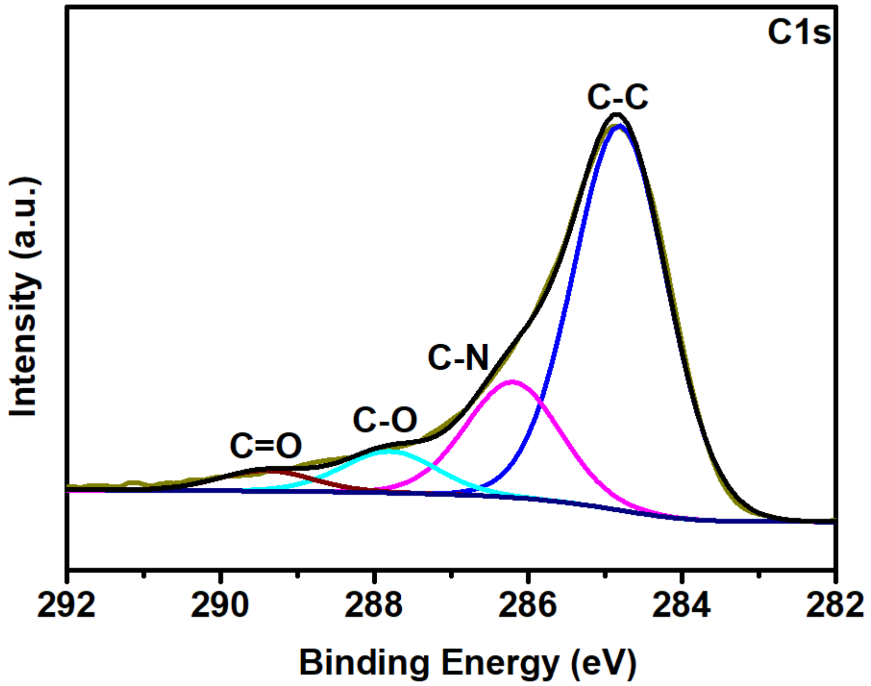

Supplementary Figure 14. C 1s XPS spectra of Co_{SA}-HC.

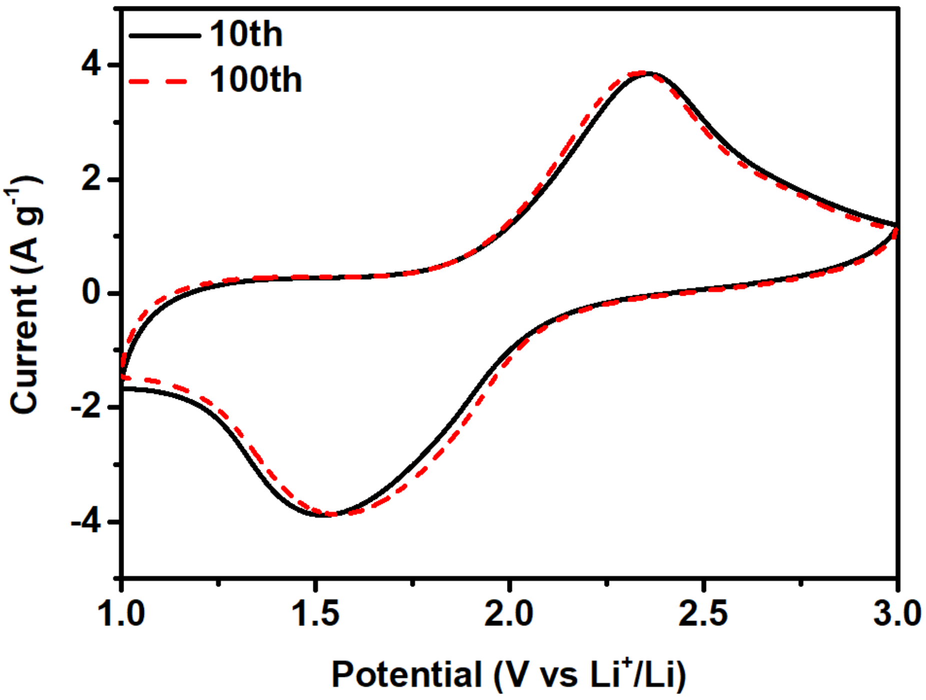

Supplementary Figure 15. CV curves of Se@Co_{SA}-HC at a scan rate of 2.0 mV/s after 10th and 100th cycle.

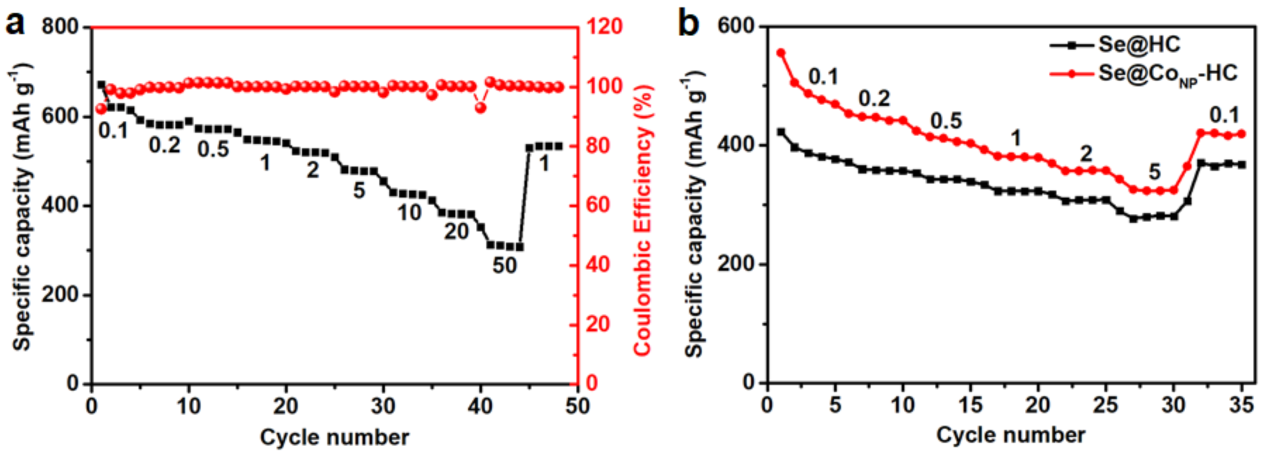

Supplementary Figure 16. (a) Rate performance of Se@Co_{SA}-HC at various current densities from 0.1 to 50 C. (b) Rate performance of Se@HC and Se@Co_{NP}-HC at various current densities from 0.1 to 5 C.

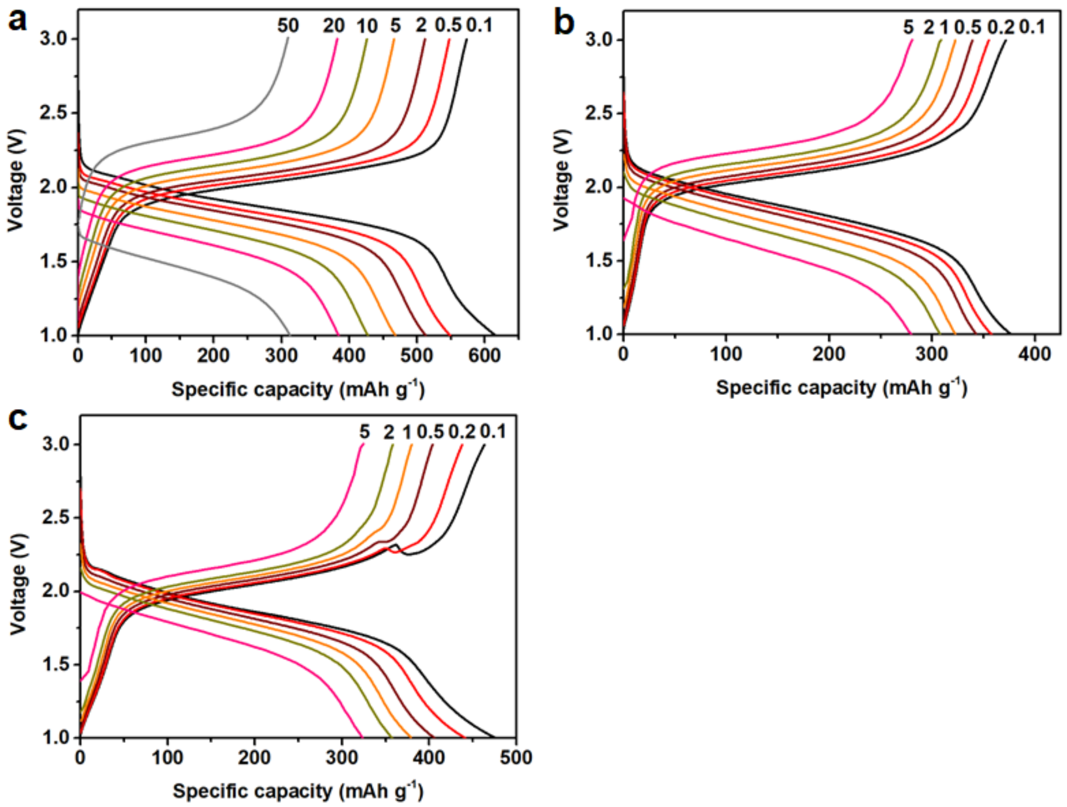

Supplementary Figure 17. (a) The charge–discharge voltage profiles of Se@Co_{SA}-HC at various current densities from 0.1 to 50 C. (b) The charge–discharge voltage profiles of Se@HC at various current densities from 0.1 to 5 C. (c) The charge–discharge voltage profiles of Se@Co_{NP}-HC at various current densities from 0.1 to 5 C.

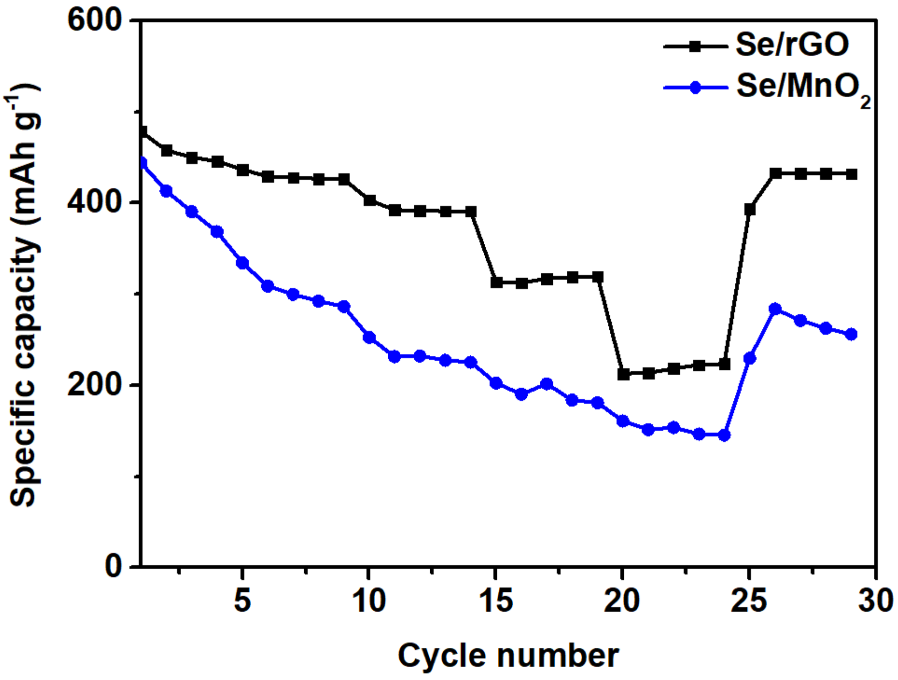

Supplementary Figure 18. Rate performance of Se/rGO and Se/MnO_2 at various current densities from 0.1 to 2 C.

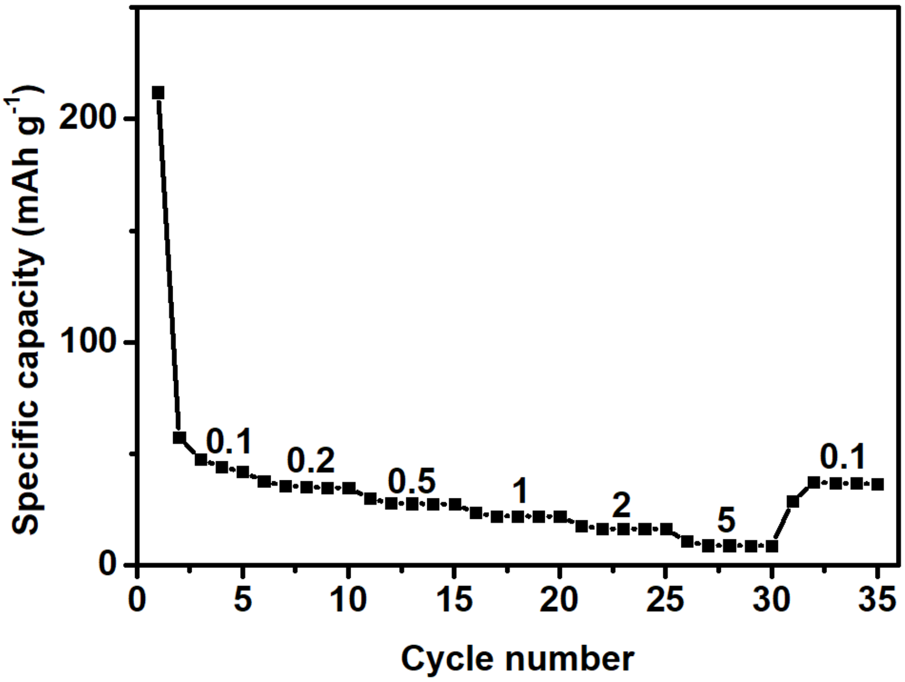

Supplementary Figure 19. Rate performances of Co_{SA}-HC at various current densities from 0.1 to 5 C.

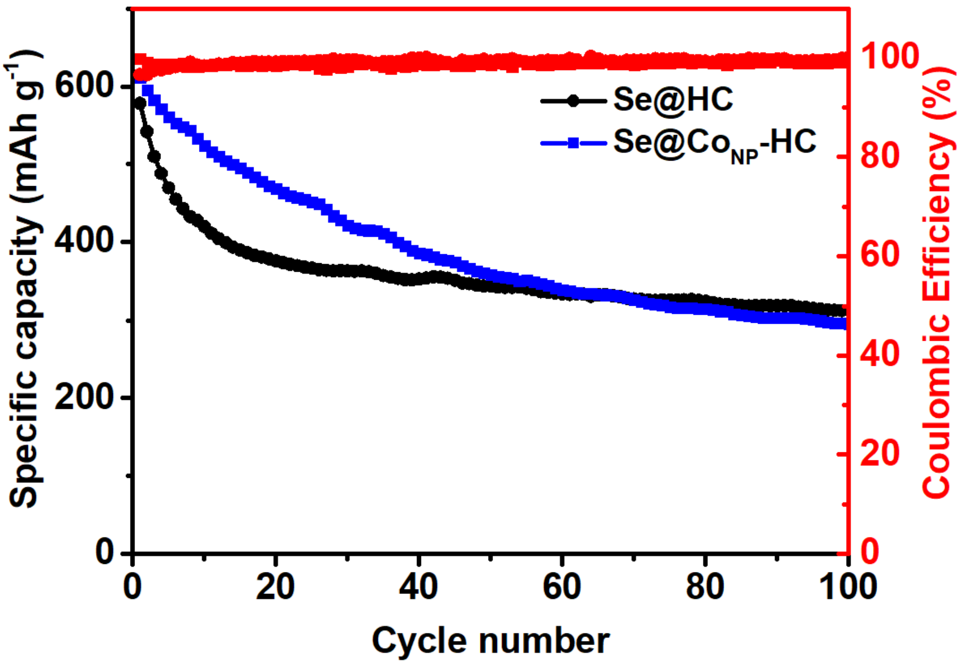

Supplementary Figure 20. Cycling performance of Se@HC and Se@Co$_{NP}$-HC at 0.5 C.

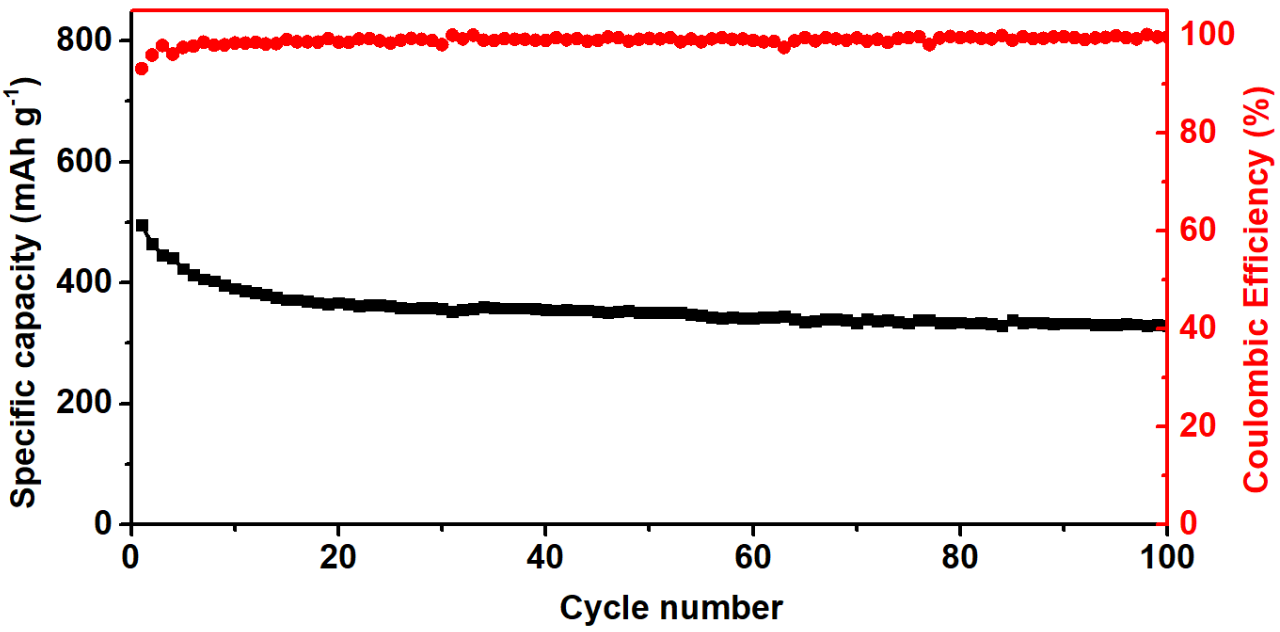

Supplementary Figure S21. Cycling performance and Coulombic efficiency for Se@Co_{SA}-HC with areal loading of selenium about 5 mg/cm^2 at 0.2 C for 100 cycles.

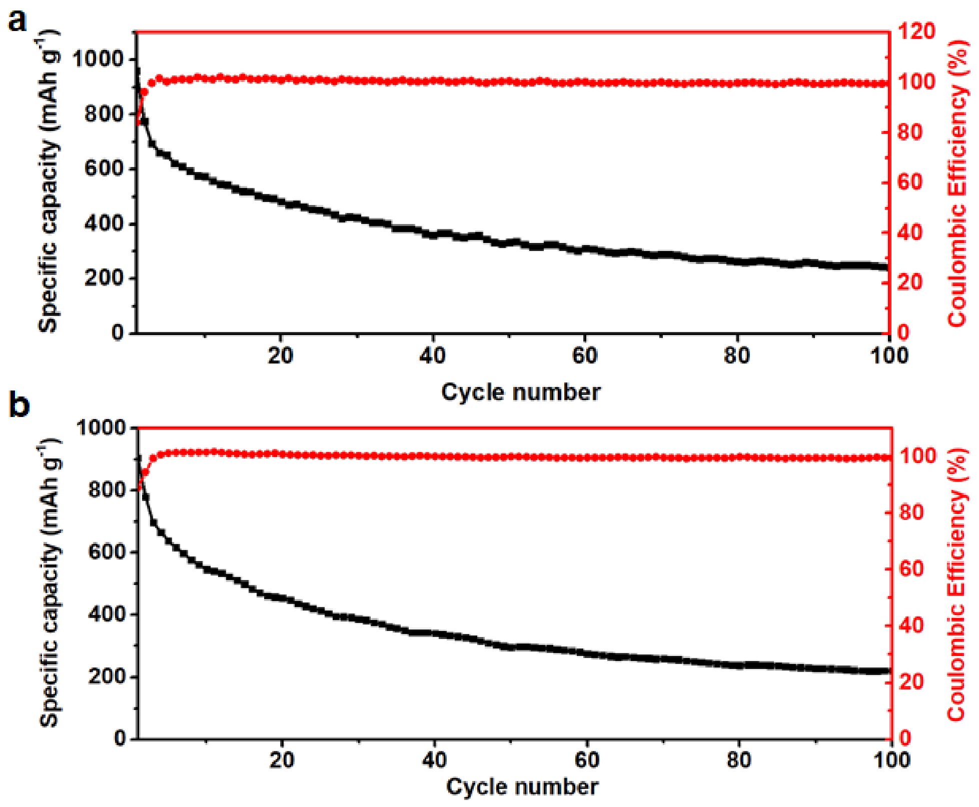

Supplementary Figure 22. Cycling performance of Se@Co_{SA}-HC with high Se loading at 0.2 C (a) and 0.5 C (b).

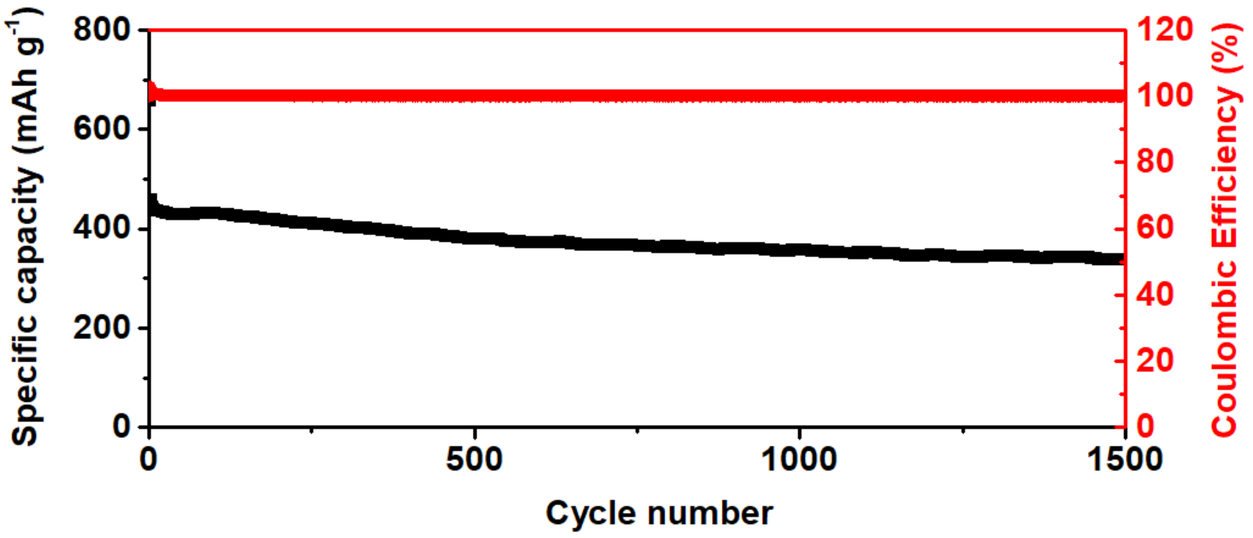

Supplementary Figure 23. Long cycle performance and coulombic efficiency of Se@Co_{SA}-HC at 5 C.

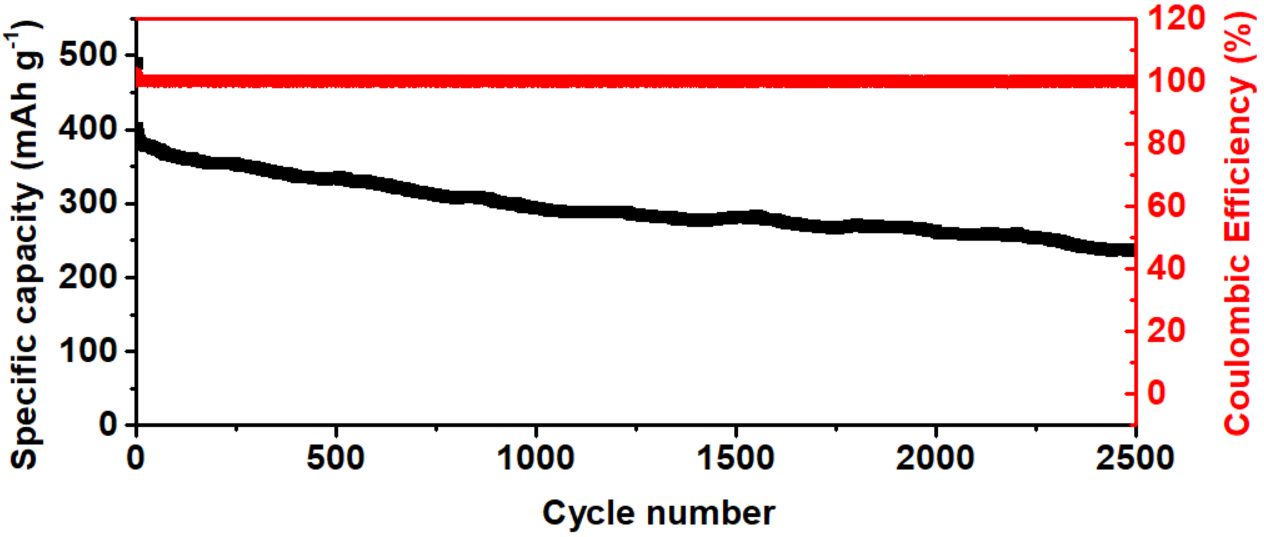

Supplementary Figure 24. Long cycle performance and coulombic efficiency of Se@Co_{SA}-HC at 20 C.

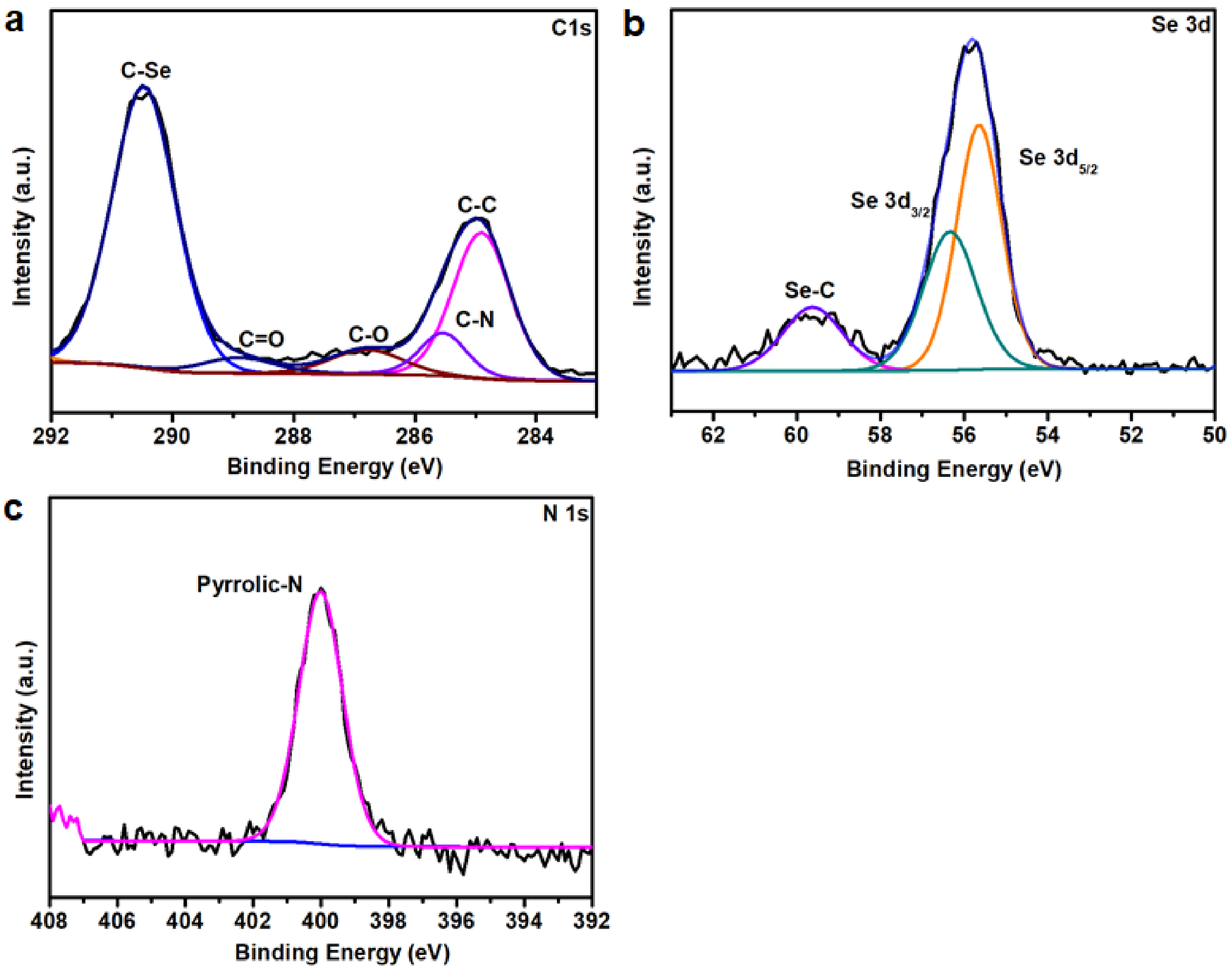

Supplementary Figure 25. (a) C 1s, (b) Se 3d and (c) N 1s XPS spectra of Se@Co$_{SA}$-HC electrode after the 1st discharge-charge cycle at 3.0 V.

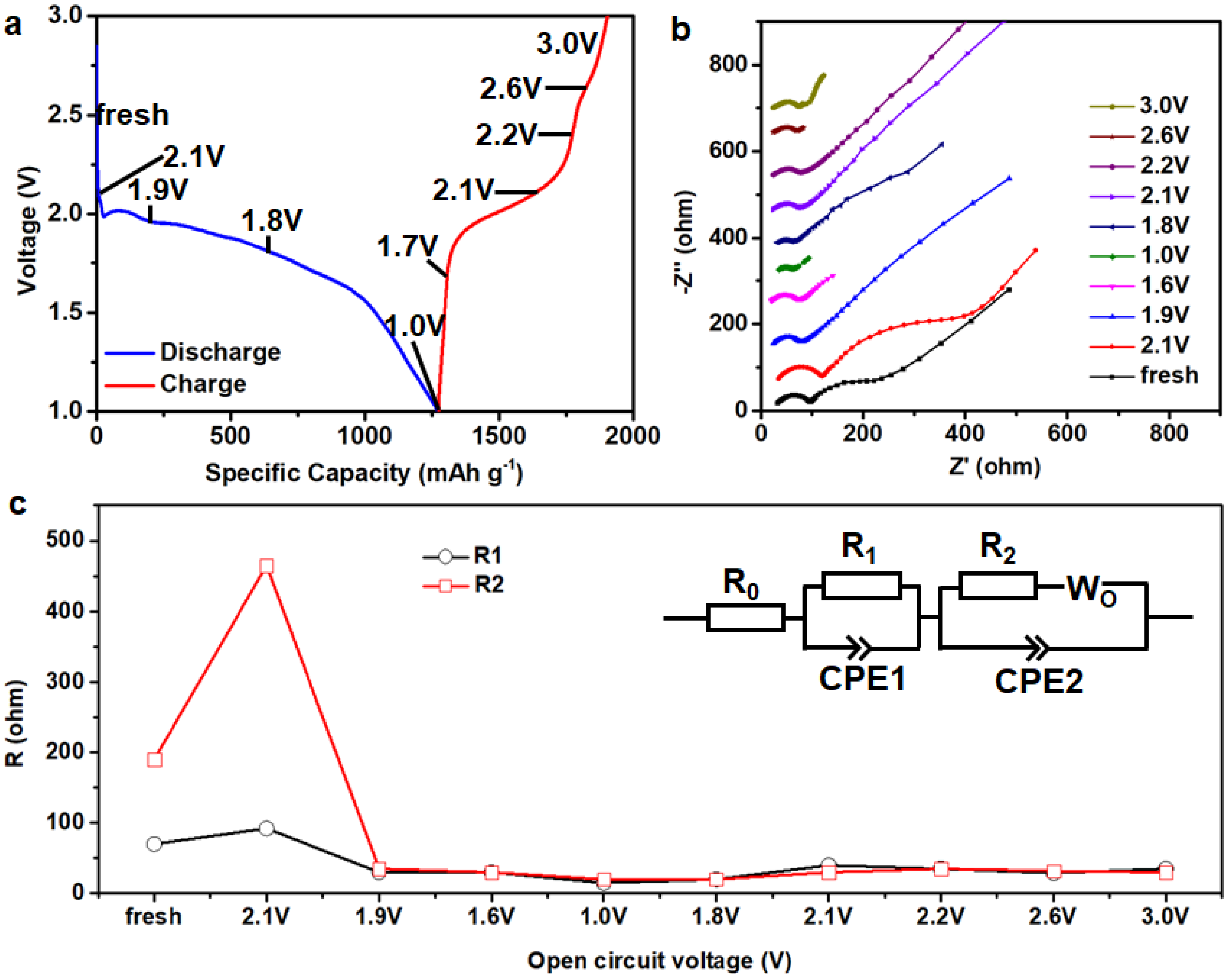

Supplementary Figure 26. EIS measurements of Se@Co_{SA}-HC composite for the Li-Se battery. (a) a typical discharge and charge profiles at 0.1 C (1st cycle, each point represents an EIS measurement). (b) EIS data at various discharging/charging stages. (c) The ohmic (R_0), charge transfer resistance (R_1), interfacial layer (R_2) resistances plotted versus various discharging/charging stages and inset is the equivalent circuit for fitting.

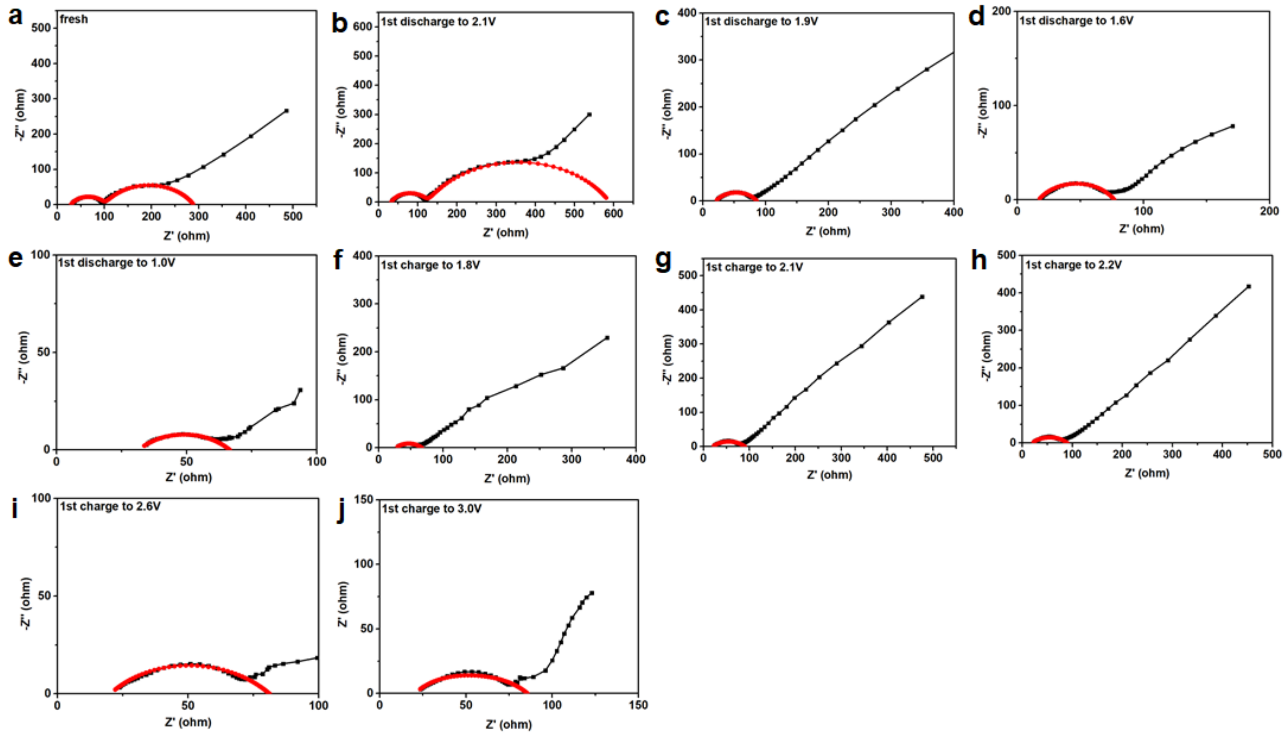

Supplementary Figure 27. Typical Nyquist plots collected at (a) fresh, (b) 1st discharge to 2.1 V, (c) 1st discharge to 1.9 V, (d) 1st discharge to 1.6 V, (e) 1st discharge to 1.0 V, (f) 1st charge to 1.8 V, (g) 1st charge to 2.1 V, (h) 1st charge to 2.2 V, (i) 1st charge to 2.6 V and (j) 1st charge to 3.0 V.

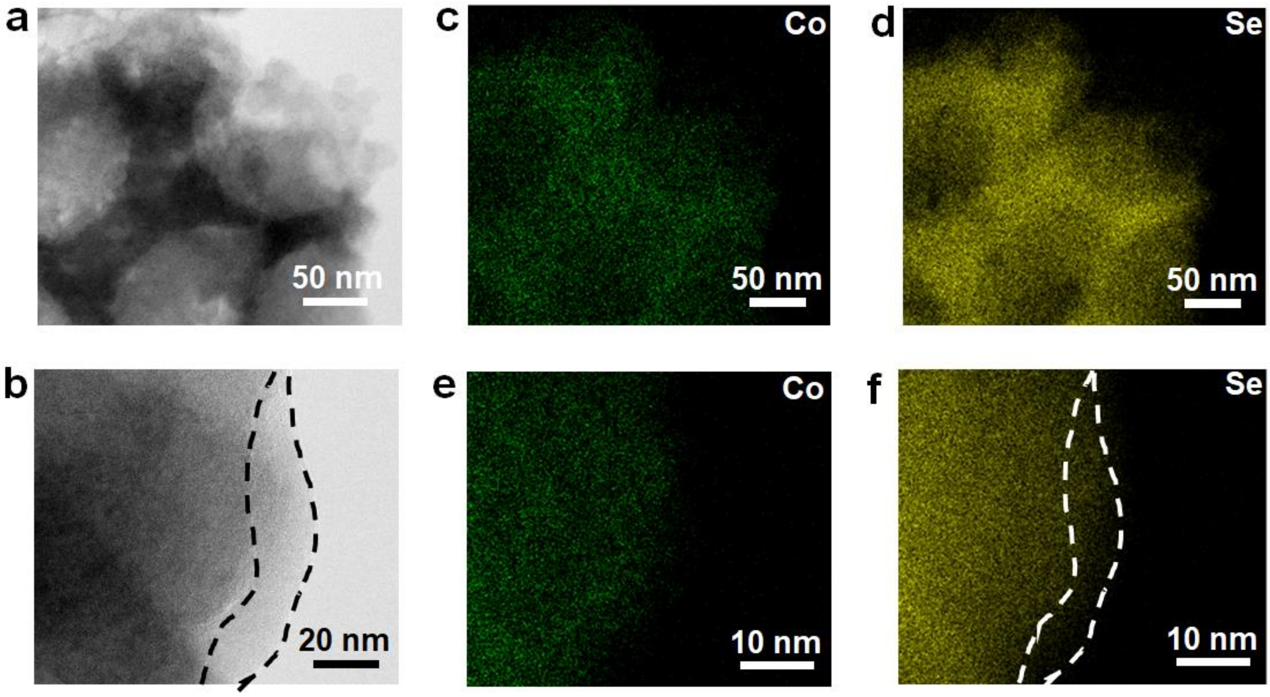

Supplementary Figure 28. (a) (b) TEM images and (c) (d) (e) (f) STEM element mapping images of the Se@Co_{SA}-HC cathode after cycling performance at 0.5 C for 1700 cycles.

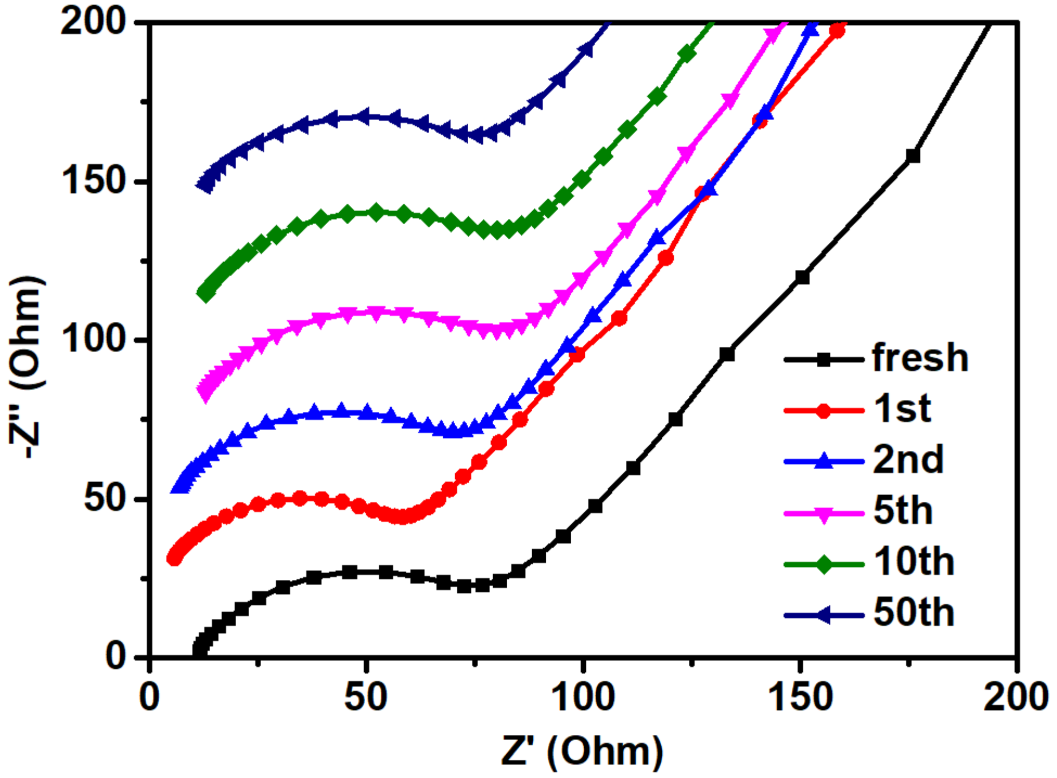

Supplementary Figure 29. EIS spectra of Se@Co$_{SA}$-HC at open-circuit potential before cycling, after 1st cycle, 2nd cycle, 5th cycle, 10th cycle and 50th cycle at 0.1 C.

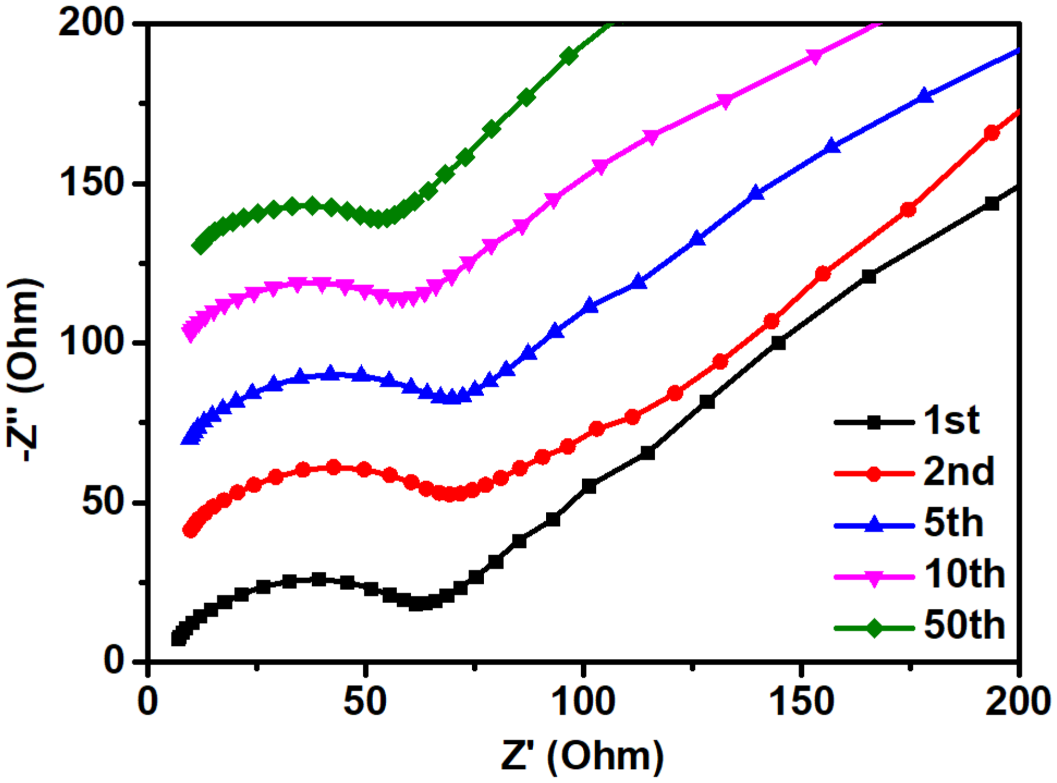

Supplementary Figure 30. EIS spectra of Se@Co_{SA}-HC at open-circuit potential after 1st cycle, 2nd cycle, 5th cycle, 10th cycle and 50th cycle at 2 C.

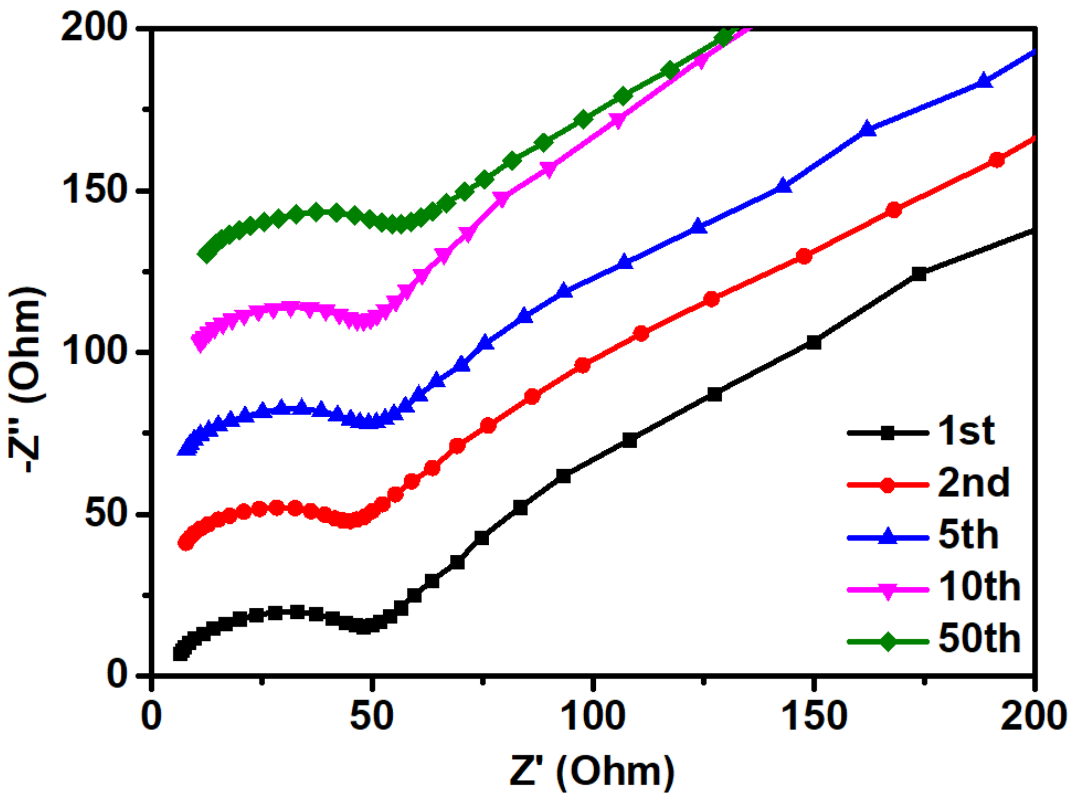

Supplementary Figure 31. EIS spectra of Se@Co_{SA}-HC at open-circuit potential after 1st cycle, 2nd cycle, 5th cycle, 10th cycle and 50th cycle at 5 C.

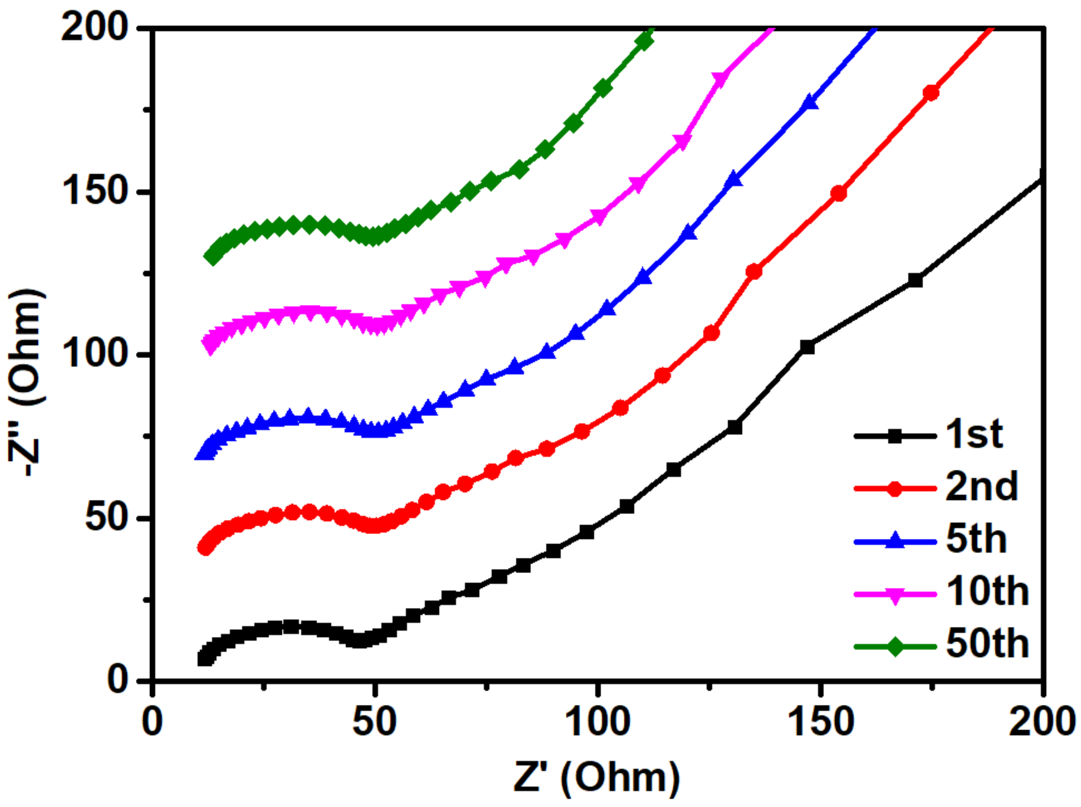

Supplementary Figure 32. EIS spectra of Se@Co_{SA}-HC at open-circuit potential after 1st cycle, 2nd cycle, 5th cycle, 10th cycle and 50th cycle at 20 C.

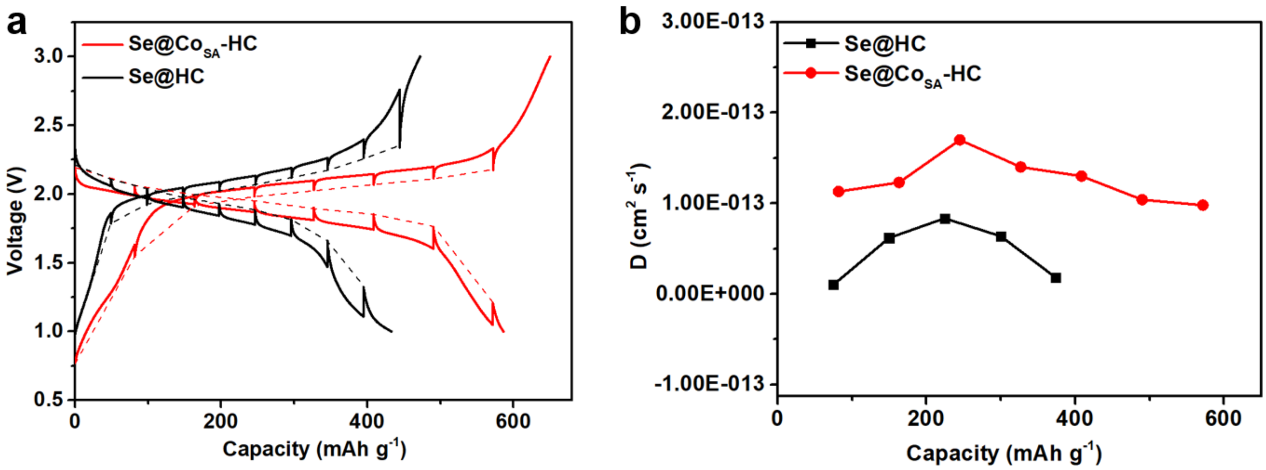

Supplementary Figure 33. (a) Galvanostatic intermittent titration technique (GITT) testing during the charge/discharge process. (b) The diffusion coefficients D_{Li}^{+} of Se@CoSA-HC and Se@HC cathode calculated from GITT plots.

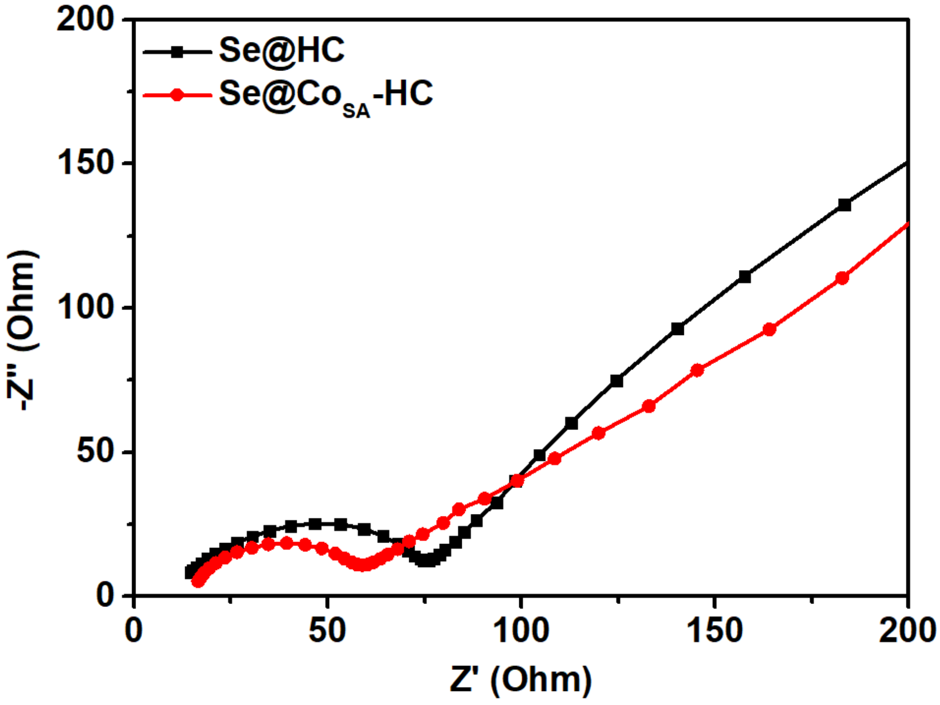

Supplementary Figure 34. EIS spectra of Se@Co_{SA}-HC and Se@HC at open-circuit potential.

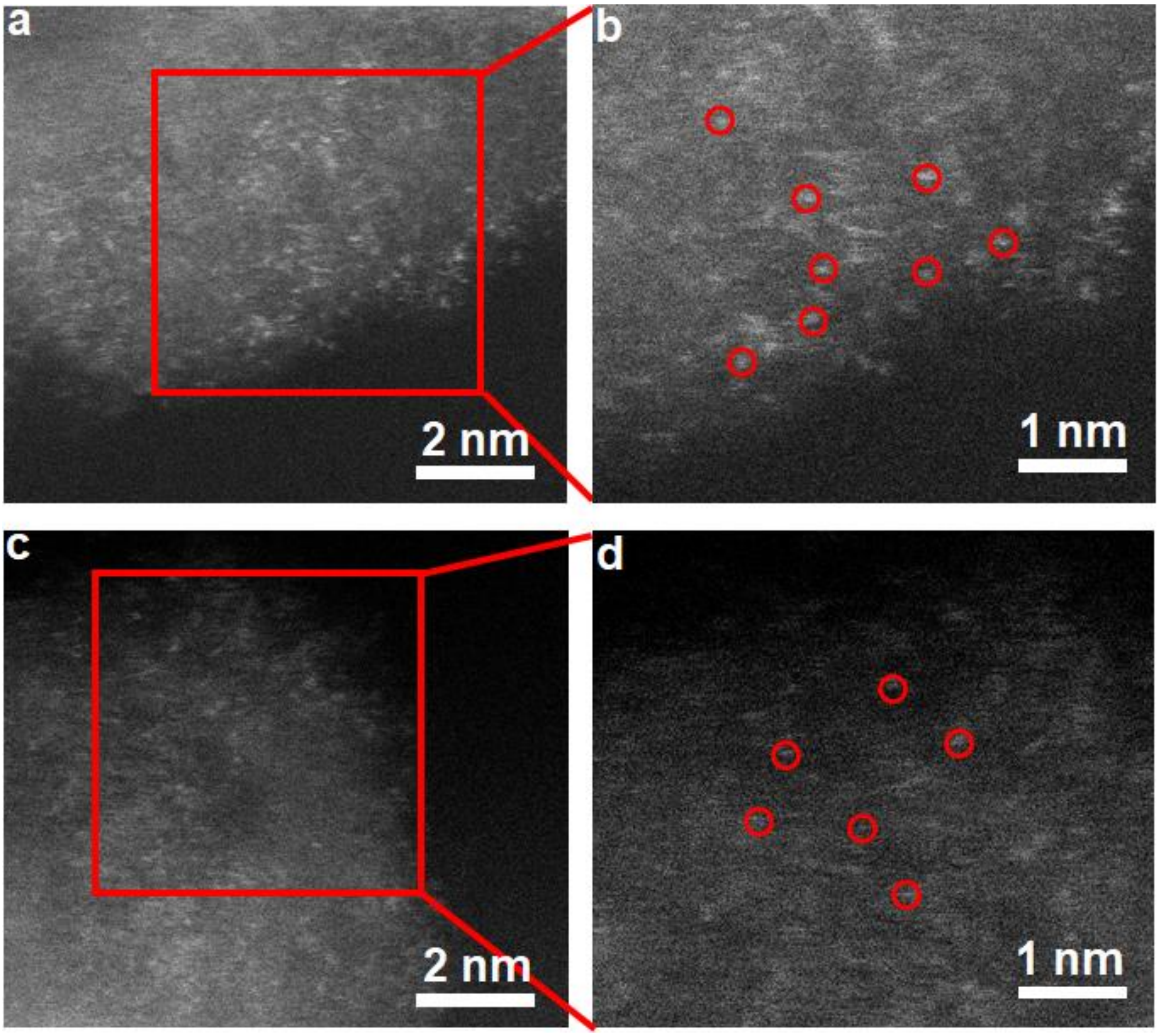

Supplementary Figure 35. (a, c) aberration-corrected HAADF-STEM images and (b, d) enlarged images of the Se@Co_{SA}-HC cathode after 1700 cycles.

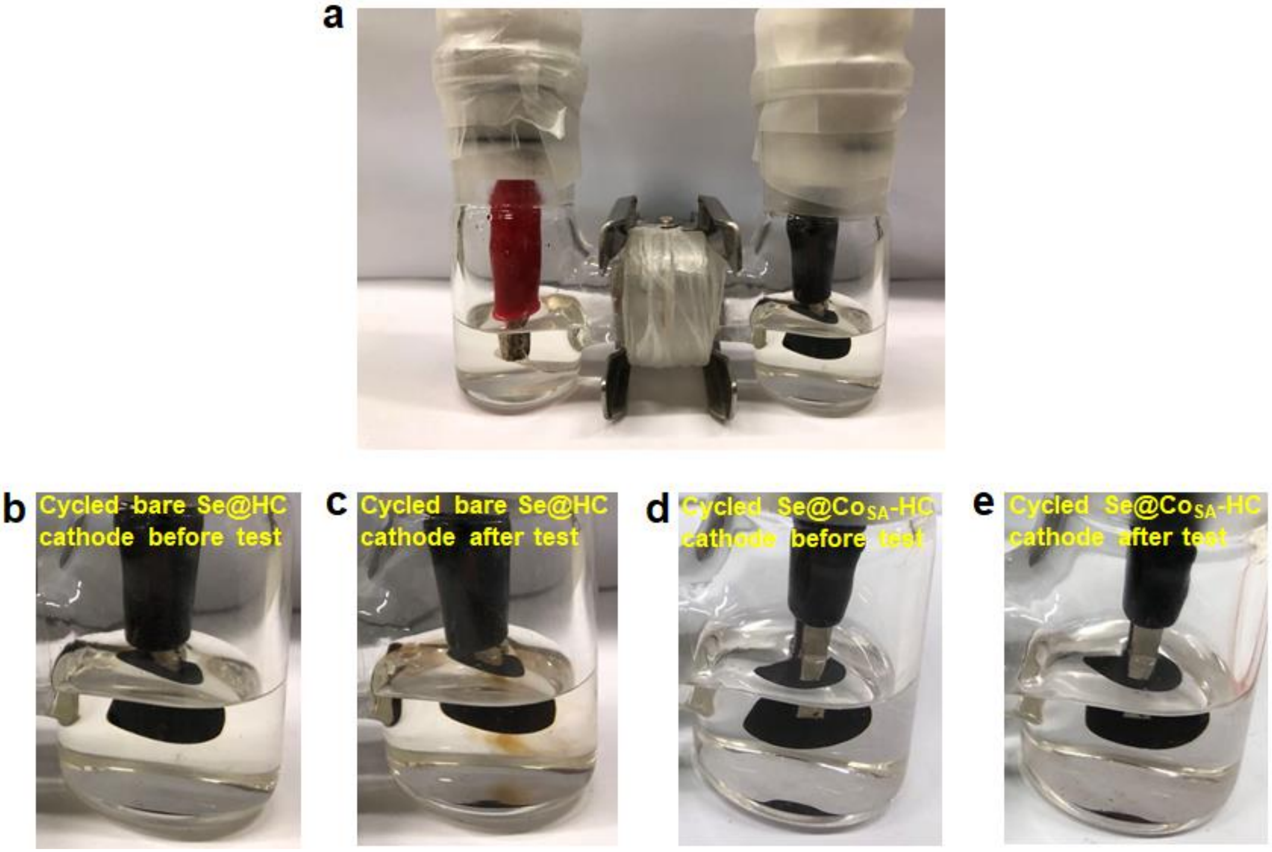

Supplementary Figure 36. Visual observation of lithium polyselenide dissolution in the electrolyte before and after cycling (3 cycles) using two types of cycled cathodes for a comparison. (a) H-type cell for Li–Se batteries, (b) cycled bare Se@HC cathode before test, (c) cycled bare Se@HC cathode after test, (d) cycled Se@Co$_{SA}$-HC cathode (after 1700 cycles) before test, (e) cycled Se@Co$_{SA}$-HC cathode after test.

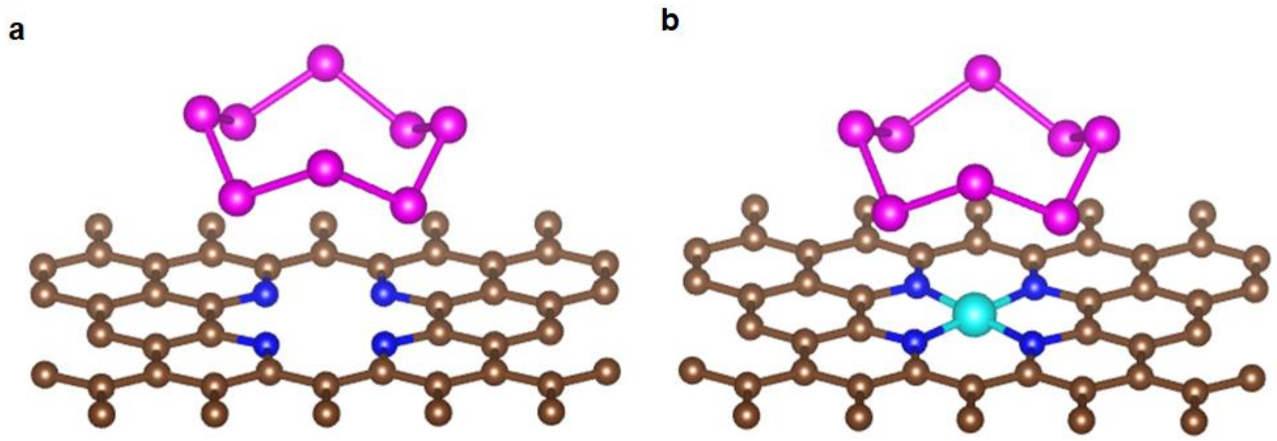

Supplementary Figure 37. Structures of NC (a) and Co-NC (b) used in first-principles calculations.

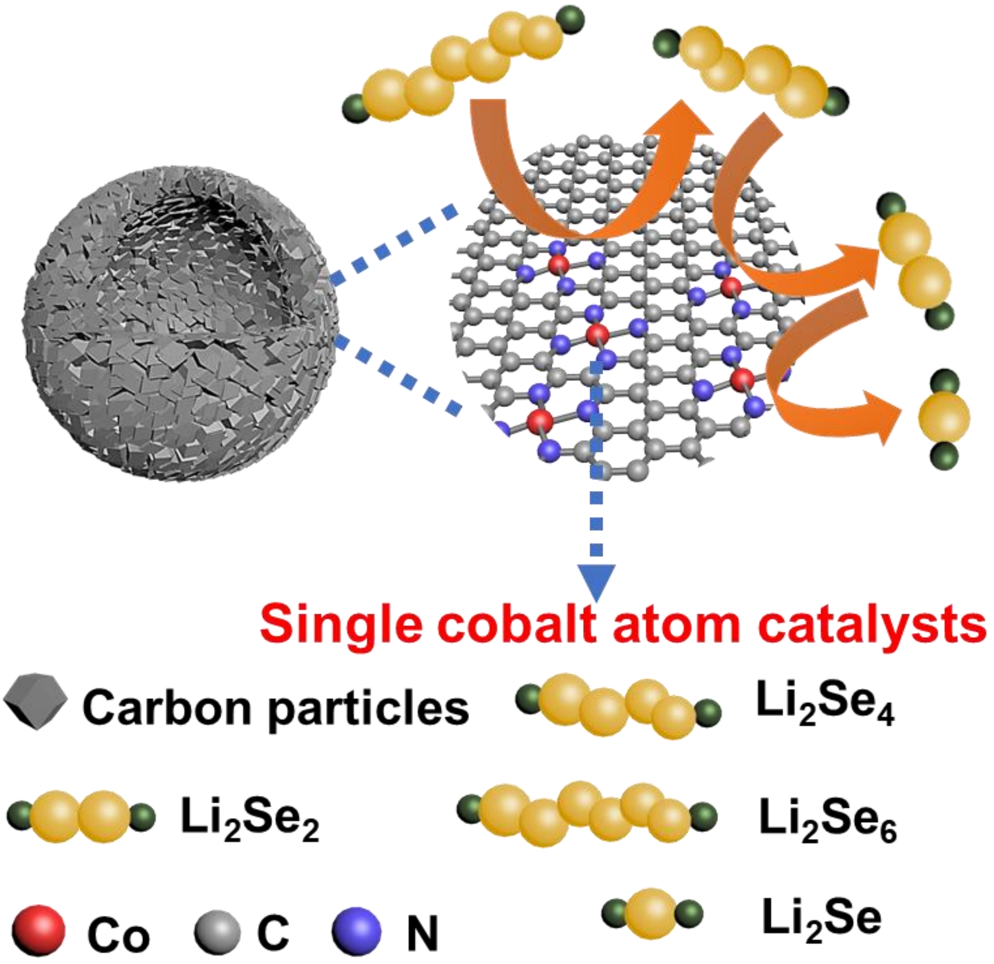

Supplementary Figure 38. Schematic illustrations of the electrode reaction mechanism of Se@Co_{SA}-HC.

Supplementary Table 1. EXAFS data fitting results of Co_{SA}-HC and CoPc.

Sample	Path	N	R (Å)	$\sigma^2(10^{-3}$ Å$^2)$
Co_{SA}-HC	Co-N	3.3	1.89	3.7
CoPc	Co-N	4	1.91	2.5

N: coordination number;

R: distance between absorber and backscatter atoms;

σ^2: the Debye-Waller factor value.

Supplementary Table 2. Comparison of electrochemical performance of various carbon based Se cathodes reported for Li-Se batteries with the results of the present study.

Sample	Se Content (Wt.%)	Reversible capacity (mAh g^{-1})/Rate)/Cycle number (^{a}1C=675 mAh g^{-1})	Rate capability (mAh g^{-1}) / Rate	Reference
Se/mesoporous carbon	30	480/0.25C/1000th	490/0.1C, 225/5C,	[1]
Se/CMK-3	49	600/0.1C/50th<br>304/1C/500th	486/1C, 311/5C	[2]
Se/carbide derived carbon	62	480/0.2C/150th	460/0.1C, 310/2C	[3]
Se/porous carbon nanofibers	52.3	643/0.075C/100th<br>516/0.75C/900th	637/0.15C, 306/6C	[4]
Se/mesoporous carbon with graphene	62	385/1C/1300th	650/0.1C, 274/3C	[5]
Se/metal−organic framework derived hollow carbon spheres	49	261/0.5C/500th	315/0.2C, 175/5C	[6]
Se/ porous carbon microcubes	50	308/0.2C/460th,<br>231/0.5C/460th,<br>166/2C/460th	420/0.2C, 225/5C	[7]
Se/dual-doped hierarchical porous carbon	48	545/0.5C/1500th	575/0.5C, 358/20C	[8]
Se/microporous carbon	56	249/1C/3000th	500/0.1C, 241/5C	[9]
Se/C composites	54	430/0.15C/250th	600/0.06C, 280/1.8C	[10]
Se/metal−organic framework derived carbon	60	588/0.5C/300th	696/0.5C, 568/10C	[11]
Se/ porous carbon polyhedrons	60	462/0.5C/1000th	564/0.5C, 409/15C	[12]
Se@Co_{SA}-HC	57	564/0.1C/100th,<br>340/5C/1500th,<br>237/20C/2500th<br>267/50C/5000th	613/0.1C,385/20C,<br>311/50C	**This work**
Se@Co_{SA}-HC	73	242/0.2C/100th<br>220/0.5C/100th		**This work**

Supplementary Table 3. The resistance values obtained from the typical Nyquist plots collected at different discharge-charge state.

Number	State	R_0 (ohm)	R_1(ohm)	R_2(ohm)
1	fresh	29	70	190
2	1st discharge to 2.1 V	32	92.15	465
3	1st discharge to 1.9 V	21	30	35
4	1st discharge to 1.6 V	18	30	30
5	1st discharge to 1.0 V	30	15	20
6	1st charge to 1.8 V	27	20	20
7	1st charge to 2.1 V	20	40	30
8	1st charge to 2.2 V	20	35	35
9	1st charge to 2.6 V	20	29	32
10	1st charge to 3.0 V	21	35	30

Supplementary Table 4. Reaction energies (in eV) of each step for the Se_8 and polyselenide Li_2Se_n electrochemical reduction on NC and Co-NC systems.

	Step	Reduction reactions	Gibbs free energy ΔG(eV)
NC	1	Se_8/NC+2Li=Li_2Se_8/NC	-4.94
	2	Li_2Se_8/NC=Li_2Se_6/NC+0.25Se_8	-0.06
	3	Li_2Se_6/NC=Li_2Se_4/NC+0.25Se_8	0.11
	4	Li_2Se_4/NC=Li_2Se_2/NC+0.25Se_8	0.45
	5	Li_2Se_2/NC=Li_2Se/NC+0.125Se_8	0.96
Co-NC	1	Se_8/HC+2Li=Li_2Se_8/HC	-2.36
	2	Li_2Se_8/HC=Li_2Se_6/HC+0.25Se_8	-0.20
	3	Li_2Se_6/HC=Li_2Se_4/HC+0.25Se_8	0.22
	4	Li_2Se_4/HC=Li_2Se_2/HC+0.25Se_8	0.77
	5	Li_2Se_2/HC=Li_2Se/HC+0.125Se_8	0.85

Supplementary Table 5. DFT calculations of the configurations and Li-Se bond length (Å) of lithium polyselenides on NC and Co-NC supports. The brown, pink, green, blue, and cyan balls represent C, Se, Li, N, and Co atoms, respectively.

NC	Configurations and Li-Se bond length (Å)	Co-NC	Configurations and Li-Se bond length (Å)
	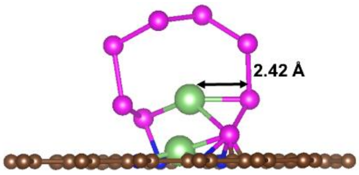 Li_2Se_8		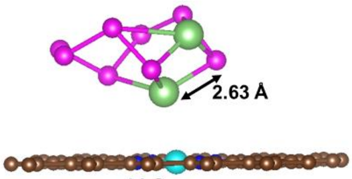 Li_2Se_8
	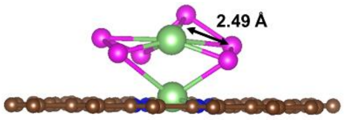 Li_2Se_6		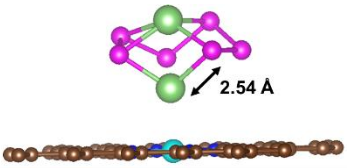 Li_2Se_6
	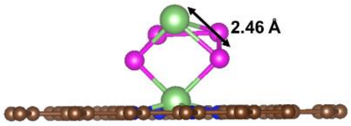 Li_2Se_4		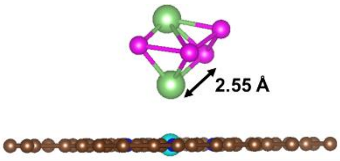 Li_2Se_4
	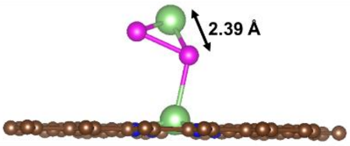 Li_2Se_2		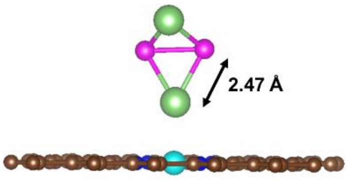 Li_2Se_2
	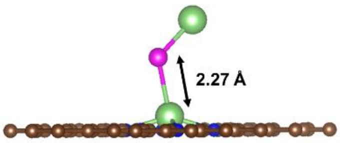 Li_2Se		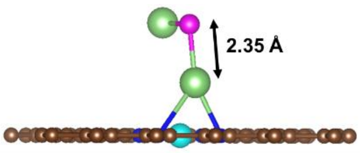 Li_2Se

Supplementary Note 1

High-power lithium-selenium batteries enabled by atomic cobalt electrocatalyst in hollow carbon cathode

Tian et al.

Galvanostatic intermittent titration technique (GITT) testing was employed to determine the diffusion coefficient in Li-Se batteries. In this characterization, GITT was carried out with a current pulse of 50 mA g^{-1} for 1h followed by a 3 h relaxation process to achieve a balance state during the charge/discharge processes. The diffusion coefficient (D_{Li^+}) was calculated based on the equation (1) as follows:

$$D_{Li+} = \frac{4}{\pi\tau}(\frac{m_B V_M}{M_B A})^2(\frac{\Delta E_s}{\Delta E_\tau})^2(\tau << L^2 / D_{Li+})$$

Supplementary Equation 1

Where τ is the duration time of the current pulse, m_B is the mass of the active material, V_M is the molar volume (16.45 cm^3 mol^{-1}), M_B is the molecular weight (78.97 g mol^{-1}) and A is the total contacting area of electrode with electrolyte (1.13 cm^2). ΔE_S is the difference between two consecutive stable voltages after relaxation, ΔE_τ is the transient voltage-change during a single titration step and the L is the thickness of the cathode.

Supplementary Note 2

High-power lithium-selenium batteries enabled by atomic cobalt electrocatalyst in hollow carbon cathode

Tian et al.

The exchange current density i_0 was calculated by the Butler–Volmer equation (2) as follows:

$$i_0 = RT / nFAR_{ct}$$ Supplementary Equation 2

Where R is the gas constant (8.3145 J/mol/K) and T is the absolute temperature. The n is the number of transferred electrons; F is the Faraday constant (96485 C/mol). A is the surface areas and R_{ct} is charge transfer resistance.

Supplementary References:

1. Luo, C.; Xu, Y.; Zhu, Y.; Liu, Y.; Zheng, S.; Liu, Y.; Langrock, A.; Wang, C., Selenium@Mesoporous Carbon Composite with Superior Lithium and Sodium Storage Capacity. *ACS Nano* **2013,** *7*, 8003-8010.

2. Yang, C.-P.; Xin, S.; Yin, Y.-X.; Ye, H.; Zhang, J.; Guo, Y.-G., An Advanced Selenium–Carbon Cathode for Rechargeable Lithium–Selenium Batteries. *Angew. Chem. Int. Ed.* **2013,** *52*, 8363-8367.

3. Lee, J. T.; Kim, H.; Oschatz, M.; Lee, D.-C.; Wu, F.; Lin, H.-T.; Zdyrko, B.; Cho, W. I.; Kaskel, S.; Yushin, G., Micro- and Mesoporous Carbide-Derived Carbon–Selenium Cathodes for High-Performance Lithium Selenium Batteries. *Adv. Energy Mater.* **2015,** *5*, 1400981.

4. Zeng, L.; Zeng, W.; Jiang, Y.; Wei, X.; Li, W.; Yang, C.; Zhu, Y.; Yu, Y., A Flexible Porous Carbon Nanofibers-Selenium Cathode with Superior Electrochemical Performance for Both Li-Se and Na-Se Batteries. *Adv. Energy Mater.* **2015,** *5*, 1401377.

5. Han, K.; Liu, Z.; Shen, J.; Lin, Y.; Dai, F.; Ye, H., A Free-Standing and Ultralong-Life Lithium-Selenium Battery Cathode Enabled by 3D Mesoporous Carbon/Graphene Hierarchical Architecture. *Adv. Funct. Mater.* **2015,** *25*, 455-463.

6. Liu, T.; Dai, C.; Jia, M.; Liu, D.; Bao, S.; Jiang, J.; Xu, M.; Li, C. M., Selenium Embedded in Metal–Organic Framework Derived Hollow Hierarchical Porous Carbon Spheres for Advanced Lithium–Selenium Batteries. *ACS Appl. Mater. Interfaces* **2016,** *8*, 16063-16070.

7. Liu, T.; Jia, M.; Zhang, Y.; Han, J.; Li, Y.; Bao, S.; Liu, D.; Jiang, J.; Xu, M., Confined selenium within metal-organic frameworks derived porous carbon microcubes as cathode for rechargeable lithium–selenium batteries. *J. Power Sources* **2017,** *341*, 53-59.

8. Zhao, X.; Yin, L.; Zhang, T.; Zhang, M.; Fang, Z.; Wang, C.; Wei, Y.; Chen, G.; Zhang, D.; Sun, Z.; Li, F., Heteroatoms dual-doped hierarchical porous carbon-selenium composite for durable Li–Se and Na–Se batteries. *Nano Energy* **2018,** *49*, 137-146.

9. Liu, Y.; Si, L.; Zhou, X.; Liu, X.; Xu, Y.; Bao, J.; Dai, Z., A selenium-confined microporous carbon cathode for ultrastable lithium–selenium batteries. *J. Mater. Chem. A* **2014,** *2*, 17735-17739.

10. Luo, C.; Wang, J.; Suo, L.; Mao, J.; Fan, X.; Wang, C., In situ formed carbon bonded and encapsulated selenium composites for Li–Se and Na–Se batteries. *J. Mater. Chem. A* **2015,** *3*, 555-561.

11. Park, S.-K.; Park, J.-S.; Kang, Y. C., Selenium-infiltrated metal–organic framework-derived porous carbon nanofibers comprising interconnected bimodal pores for Li–Se batteries with high capacity and rate performance. *J. Mater. Chem. A* **2018,** *6*, 1028-1036.

12. Park, S.-K.; Park, J.-S.; Kang, Y. C., Metal-Organic-Framework-Derived N-Doped Hierarchically Porous Carbon Polyhedrons Anchored on Crumpled Graphene Balls as Efficient Selenium Hosts for High-Performance Lithium–Selenium Batteries. *ACS Appl. Mater. Interfaces* **2018,** *10*, 16531-16540.
