# Supplementary material for: High-power lithium–selenium batteries enabled by atomic cobalt electrocatalyst in hollow carbon cathode
Source: Nat Commun. 2020 Oct 6;11:5025. doi: 10.1038/s41467-020-18820-y (PMC7538427; doi:10.1038/s41467-020-18820-y)
Supplement: Supplementary file 2 — Peer Review File [file 41467_2020_18820_MOESM2_ESM.pdf]

Reviewer #1 (Remarks to the Author):

This work reports the construction of cobalt single atoms/nitrogen-doped hollow porous carbon (CoSA-HC) with their outstanding electrochemical behaviour for lithium selenium batteries. The issue of the shuttle effect of polyselenides has been well addressed by CoSA-HC particles designed by the authors, the prepared selenium carbon composites exhibited excellent rate and cycling performance for Li-Se batteries. Most importantly, it provides the concept of using MOFs to construct cobalt single atoms to activate selenium reactivity and immobilize selenium and polyselenides. It is a breakthrough in the area of lithium selenium batteries, would generate a new research direction in single atom catalysis for long cycle life and high-power Li-Se batteries. The manuscript is well organized and written, the discussion is insights. Based on these considerations, I suggest it be acceptable for publication in Nat. Commun. after minor revision by addressing the following questions.

1. The author prepared a series of hollow structured materials with cobalt single atoms and large cobalt particles. How the cobalt single atoms are generated in the hollow carbon structures?
2. It is very interesting to observe the ZIF particles can be grown on the surface of PS spheres. It is better for the authors to clarify the reason of the growth ZIF particles on the surface of PS spheres.
3. As PS spheres can be decorated with ZIFs particles, can the ZIF particles be grown on the surface of other template materials?
4. Followed the question 3, if yes, how about the electrochemical performances for Li-Se batteries by using those materials?
5. From Figure S10 in the supporting information, it can be observed that elemental selenium is uniformly distributed with the whole carbon structures. How the selenium incorporates into hollow carbon structures?

Reviewer #2 (Remarks to the Author):

This manuscript reports a selenium cathode supported by cobalt single atoms, which shows largely enhanced rate capability. The Co–N-C composites have been used as sulfur hosts for years. The manuscript does not present enough marked scientific novelty, and nor insights on the fundamental mechanism. So, it is not suitable for potential publication in Nature Communications.
1. In addition to the DFT calculation, the catalyst effect of the cobalt atoms needs to be demonstrated via direct electrochemical experiments. The key kinetic parameters, such as the exchange current density, the diffusion coefficient, should be provided.
2. The voltage profiles at various rates and cycles should be provided and compared.
3. To explain the EIS curve, it is proposed that a stable passivation layer forms at the surface of the Se@CoSA-HC particles during the first lithiation process. In the previous reports, all the passivation layers formed in the carbonate electrolyte, while 1,3-dioxolane and 1,2-dimethoxyethane are used as solvents in this work. How the passivation layer form?
4. With the increase of the rates, the relative ratio of capacitive contribution to the total capacity increases. Is it related with the cobalt catalyst?
5. The fitted curve should be provided in the EIS analysis.
6. In figure 5, theΔV should be pointed.
7. The areal loading of selenium in the cathode should be provided in the experimental part.

Reviewer #3 (Remarks to the Author):

The MS is suitable for publication in Nat Comm, I have the following concerns.
In this study, authors have prepared a ZnCo-ZIF, which basically is a MOF, and then pyrolyzed it to obtain porous spheres which have been labeled as Co-HC. The disappearance of Zn from the structure is not clear to me- Zn2+ ions cannot get pyrolyzed and vanish, as Scheme 1 seems to suggest- if there were any steps, after the PS-ZIF formation and before the Co-HC formation, please include them. Otherwise, it looks scientifically incorrect.

The title of this manuscript is also extremely difficult to come to terms with. Please change it to ZnCo or Co-hollow porous carbon composite with Se (which is what it is!). In general, everything is made of atoms, so just magnifying the hollow ZnCo-HC using a microscope, and labelling the atoms, does not imply that Selenium's charge storage capacity is enhanced by the Cobalt atoms, this would have been the case if they were elemental Co stand-alone atoms, but as it stands, in this MS, they are not stand-alone entities, but are very much a part of a large porous matrix, and are covalently linked to other elements like carbon and nitrogen. Further to strengthen my line of reasoning, I would like to direct your attention to the XPS core level spectrum of Co2p. Co does not exist in the fully reduced state as elemental Co, it does in Co^{2+} and Co^{3+} states, as shown therein. Whats' the third peak due to?
Figure 3: Can you show the CV curves after 10th and 100th cycle?
Why does Se becomes less crystalline once the composite is formed?

Responses to Reviewer' Comments

Responses to Reviewer #1

Reviewer Letter:

This work reports the construction of cobalt single atoms/nitrogen-doped hollow porous carbon (CoSA-HC) with their outstanding electrochemical behaviour for lithium selenium batteries. The issue of the shuttle effect of polyselenides has been well addressed by CoSA-HC particles designed by the authors, the prepared selenium carbon composites exhibited excellent rate and cycling performance for Li-Se batteries. Most importantly, it provides the concept of using MOFs to construct cobalt single atoms to activate selenium reactivity and immobilize selenium and polyselenides. It is a breakthrough in the area of lithium selenium batteries, would generate a new research direction in single atom catalysis for long cycle life and high-power Li-Se batteries. The manuscript is well organized and written, the discussion is insights. Based on these considerations, I suggest it be acceptable for publication in Nat. Commun. after minor revision by addressing the following questions.

Response:

We sincerely thank the Reviewer #1 for these very positive comments on our work. We have revised the manuscript according to the reviewer's comments. The revised manuscript and the electronic supporting information have been corrected and highlighted in yellow colour. The point-to-point responses are listed below.

1. The author prepared a series of hollow structured materials with cobalt single atoms and large cobalt particles. How the cobalt single atoms are generated in the hollow carbon structures?

Response:

The generation of atomically dispersed Co catalysts was attributed to the Co ions being reduced by carbonized organic linkers with the evaporation of elemental Zn during the calcination process[1, 2]. A brief description has been added on Page 7, line 13 to line 15 in the revised manuscript.

References:

1. X. Wang, Z. Chen, X. Zhao, T. Yao, W. Chen, R. You, C. Zhao, G. Wu, J. Wang, W. Huang, J. Yang, X. Hong, S. Wei, Y. Wu, Y. Li, *Angew. Chem. Int. Ed.* **2018**, *57*, 1944.
2. P. Yin, T. Yao, Y. Wu, L. Zheng, Y. Lin, W. Liu, H. Ju, J. Zhu, X. Hong, Z. Deng, G. Zhou, S. Wei, Y. Li, *Angew. Chem. Int. Ed.* **2016**, *55*, 10800.

2. It is very interesting to observe the ZIF particles can be grown on the surface of PS spheres. It is better for the authors to clarify the reason of the growth ZIF particles on the surface of PS spheres.

Response:

The growth of ZIF particles on the surface of PS spheres is determined by the interfacial reaction between the substrate and surfactant. Polyvinylpyrrolidone (PVP) plays an important role in the homogeneous growth of ZIFs particles on the surface of PS spheres. To improve electrostatic forces and provide enough coordination sites to uniformly adsorb metal ions, the negatively charged PS spheres are firstly enriched with PVP molecules. The amide carbonyl groups of PVP

can fully coordinate with metal ions through chemical bonds and make it possible to achieve the coating of ZIFs on the surfaces of the PS spheres[1]. This description has been added on Page 6, line 8 to line 16 in the revised manuscript.

References:
1. H. X. Zhong, J. Wang, Y. W. Zhang, W. Xu, W. Xing, D. Xu, Y. F. Zhang, and X. B. Zhang, *Angew. Chem. Int. Ed.*, **2014**, *53*, 14235-14239.

3. As PS spheres can be decorated with ZIFs particles, can the ZIF particles be grown on the surface of other template materials?

Response:

Following reviewer's comments, we have attempted to coating ZIF particles onto other templates. Besides coating on the surface of 0D (*e.g.* PS spheres) materials, ZIFs particles can be successfully grown on the surface of 1 D and 2 D materials, namely, MnO_2 nanowires (MnO_2 NWs) and reduced graphene oxide (rGO), respectively. The detailed synthesis procedure of rGO@ZIF and MnO_2@ZIFs are described as below:

Synthesis of MnO_2 nanowires: In a typical synthesis, 50 mg of polyvinylpyrrolidone (PVP) and 40 mL of 0.015M $KMnO_4$ aqueous solution were mixed with magnetic stirring, and then the mixture was transferred into a 50 mL Teflon-lined stainless autoclave. The autoclave was sealed and put in an electronic oven at 160 °C for 9 h and then naturally cooled down to room temperature. The precipitates were collected by filtration, washed with deionized water and absolute ethanol for several times before drying at 60 °C overnight.

Synthesis of reduced graphene oxide (rGO): Graphene oxide (3mg/ml) solution were dried via lyophilization and then was ground into powder. Reduced graphene oxide can be obtained through hydrothermal treatment of graphene oxide (0.4 g) with 400 µL hydrazine hydrate at 95 °C for 24 h.

Synthesis of the MnO_2@ZIF: In a typical procedure, 0.07 g of as-prepared MnO_2 nanowires were fully ground and then dispersed into 90 mL of methanol containing 1g of PVP (K-30). After ultrasonic dispersion and vigorous agitation for 3 h, 2.125 g of $Zn(NO_3)_2·6H_2O$ and 0.104 g of $Co(NO_3)_2·6H_2O$ (the molar ratio of Zn^{2+}/Co^{2+} was 20) were subsequently dissolved into the mixed solution and stirred for another 0.5 h. Then, 4.926 g of 2-methylimidazole dissolved in 90 mL of methanol was quickly added into the above solution followed by vigorous stirring for 4 h. Finally, the MnO_2@ZIF precursors were collected by centrifugation, washed with methanol for several times, and dried at 60 °C overnight.

Synthesis of rGO@ZIF: In a typical procedure, 0.07 g of as-prepared rGO were fully ground and then dispersed into 90 mL of methanol containing 1g of PVP (K-30). After ultrasonic dispersion and vigorous agitation for 3 h, 2.125 g of $Zn(NO_3)_2·6H_2O$ and 0.104 g of $Co(NO_3)_2·6H_2O$ (the molar ratio of Zn^{2+}/Co^{2+} was 20) were subsequently dissolved into the mixed solution and stirred for another 0.5 h. Then, 4.926 g of 2-methylimidazole dissolved in 90 mL of methanol was quickly added into the above solution followed by vigorous stirring for 4 h. Finally, the rGO@ZIF precursors were collected by centrifugation, washed with methanol for several times, and dried at 60 °C overnight.

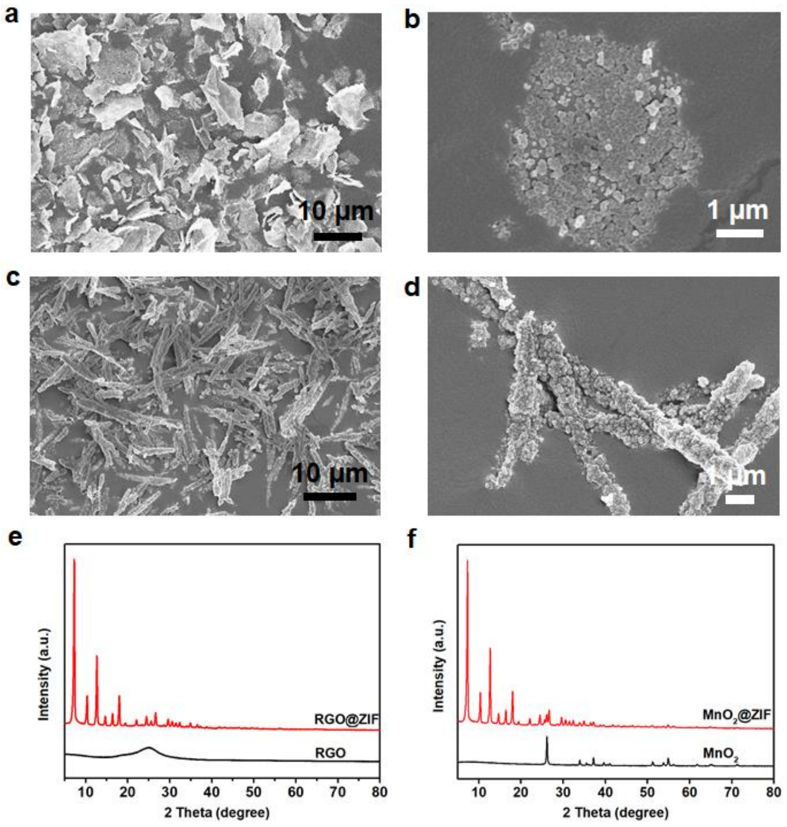

Figure R1. Low-magnification (a) and high-magnification (b) SEM image of rGO@ZIF; Low-magnification (c) and high-magnification (d) SEM image of MnO_2@ZIF; (e) XRD pattern of rGO and rGO@ZIF; (f) XRD pattern of MnO_2 and MnO_2@ZIF.

This growth strategy can be extended to the fabrication of other types of ZIFs with different dimensions and components. The SEM images in Figure **R1a-1d** show the successful preparation of rGO@ZIF and MnO_2@ZIF. The XRD results for rGO@ZIF and MnO_2@ZIF particles presented in Figure **R1e** and **R1f** agree well with that of as-prepared bimetallic ZnCo-ZIFs[1], confirming the formation of bimetallic ZIF structures.

Based on these considerations, we have further modified our manuscript as follows. The synthesis procedures of rGO@ZIF and MnO_2@ZIFs have been added in the supporting information on Page 4. The **Figure R1** has been added in the supporting information as **Figure S3**. The following description has been added in the revised manuscript on Page 6.

This growth strategy can be extended to the fabrication of other types of ZIFs with different dimensions and components. The SEM images and XRD patterns in **Figure S3** show the successful formation of core-shell structured rGO@ZIF and MnO_2@ZIF.

References:

1. Y. Z. Chen, C. Wang, Z. Y. Wu, Y. Xiong, Q. Xu, S. H. Yu and H. L. Jiang, *Adv. Mater.* **2015**, *27*, 5010-5016.

4. Followed the question 3, if yes, how about the electrochemical performances for Li-Se batteries by using those materials?

Response:

These materials can be used as hosts as the Li-Se cathodes. We have tested the electrochemical performance of Se based cathodes using rGO@ZIF and MnO_2@ZIF derived substrates for Li-Se batteries. The prepared rGO@ZIF and MnO_2@ZIFs particles were calcined at 700 °C under a N_2 atmosphere, followed by the selenium impregnation to achieve Se/rGO and Se/MnO composites. Compared with Se/rGO and Se/MnO electrodes (**Figure R2**), Se@Co_{SA}-HC cathodes exhibit superior rate electrochemical performances mainly due to the enhanced conductive network, which is also reported rGO-based electrodes for Li-Se batteries.[1] Overall, this growth strategy can be extended to the fabrication of other types of ZIFs with different dimensions and components for the applications in Li-Se batteries.

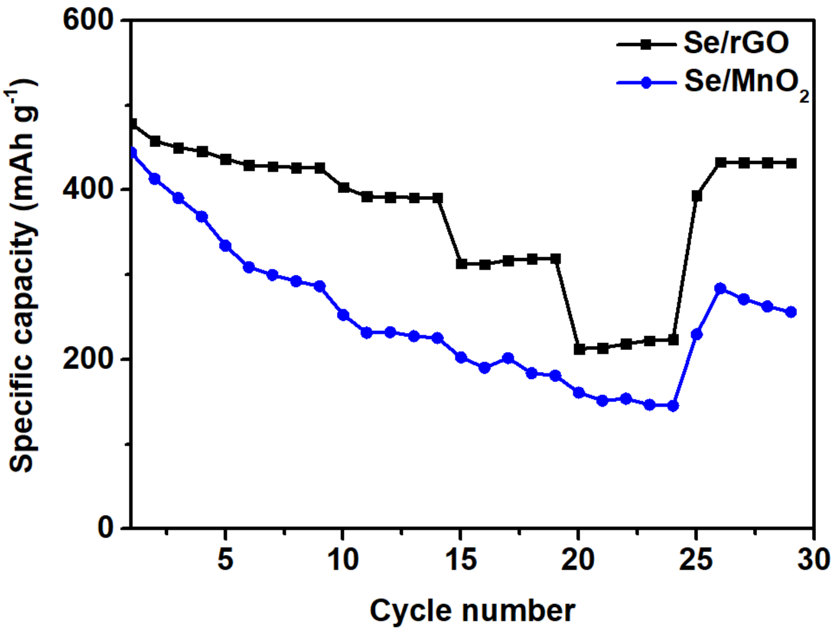

Figure R2. Rate performance of Se/rGO and Se/MnO_2 at various current densities from 0.1 to 2 C.

Based on these considerations, we have further modified our manuscript as follows. The **Figure R2** has been added in the supporting information as **Figure S18**. The following description has been added in the revised manuscript on Page 12.

Compared with Se/rGO and Se/MnO_2 electrodes (**Figure S18**), Se@Co_{SA}-HC cathodes exhibit superior rate electrochemical performances mainly due to the enhanced conductive network, which was previously reported using rGO-based electrodes for Li-Se batteries.[1]

Reference:

1. L. Zeng, X. Chen, R. Liu, L. Lin, C. Zheng, L. Xu, F. Luo, Q. Qian, Q. Chen and M. Wei, *J. Mater. Chem. A*, **2017**,*5*, 22997-23005

5. From Figure S10 in the supporting information, it can be observed that elemental selenium is uniformly distributed with the whole carbon structures. How the selenium incorporates into hollow carbon structures?

Response:

Selenium can be diffused into pores of hollow carbon via an impregnation method, which is similar to the preparation of sulphur/carbon composites. The synthesis of selenium carbon composites is described as below:

Se powder (99.99%, Sigma-Aldrich) and the as-prepared Co_{SA}-HC, HC, Co_{NP}-HC particles with a weight ratio of 1:1 were mixed. Subsequently, the mixture was heated at 300 °C for 12 h with heating rate of 5 °C min^{-1} in a tubular furnace under argon atmosphere to achieve selenium carbon composites. The as-prepared materials were named Se@Co_{SA}-HC, Se@HC and Se@Co_{NP}-HC based on the different carbon precursors.

To achieve high selenium mass ratio in selenium carbon composite, Se powder and the as-prepared Co_{SA}-HC particles with a weight ratio of 3:1 were mixed, followed by the same heat treatment as described above.

By using this method, selenium can be uniformly distributed to the substrate, leading to small particle size and high surface area, thereby achieving superior electrochemical performance.

“Selenium encapsulation into carbon particles was carried out at 300 °C under Ar atmosphere to promote the infusion of Se into the microporous carbon clusters.” This description has been added on Page 9, line 10 to line 11 in the revised manuscript.

Responses to Reviewer #2

This manuscript reports a selenium cathode supported by cobalt single atoms, which shows largely enhanced rate capability. The Co–N-C composites have been used as sulfur hosts for years. The manuscript does not present enough marked scientific novelty, and nor insights on the fundamental mechanism. So, it is not suitable for potential publication in Nature Communications.

Response:

We would like to thank the reviewer for his/her time and valuable comments to improve our manuscript. All reviewer's concerns have been carefully addressed. Following his/her suggestions, we have conducted further experimental investigations and corrected our manuscript accordingly.

We agree with the reviewer's comment on "The Co-N-C composites have been used as sulfur hosts for years." According to recent literature review, Co-N-C nanoarchitectures including cobalt-embedded carbon nanosheets, cobalt and N-doped graphitic carbon, honeycomb-like Co@N−C composite and atomic cobalt-decorated hollow carbon nanospheres have been demonstrated to effectively suppress shuttle effect of polysulfides and to enhance sulfur utilization, specific capacities, areal capacities, and cyclic stability for lithium and sodium sulfur batteries.[1-6] For example, Li *et al.* reported a novel sulfur host material based on honeycomb-like Co@N−C composite for lithium sulfur batteries with high sulfur loading, cycle stability and rate performance.[2] However, this Co-N-C composite derived from CoAl-LDH consists of nanosized Co nanocrystals, which cannot distribute uniformly in N-doped carbon matrix. Zhang et al. prepared a sulfur cathode comprised of atomic cobalt-decorated hollow carbon nanospheres to enhance sulfur reactivity and to electrocatalytically reduce polysulfide for sodium-sulfur batteries with a high reversible capacity and an excellent rate capability.[3] This cathode presented dramatic capacity fading due to the low porosity on the wall of hollow carbon, which cannot effectively supress the shuttle effect of polysulfides. Therefore, it is crucial to develop efficient hosts with favorable features, including highly effective and dispersed single atomic catalysts via stable formation of Co-N coordination and hierarchical micro/mesoporous structure to achieve outstanding performance for sulfur based batteries. This principle is also applied to selenium-based storage systems, due to the similar chemistry between Se and S based batteries systems. Typically, selenium owns similar chemical properties to sulphur and has been considered as an alternative cathode material for lithium-selenium battery because of its high theoretical volumetric capacity.[7-8] In additional, the conductivity of Se (1×10^{-3} S/m) is much higher than that of S (5×10^{-30} S/m), which enables higher active material utilization and better rate capability.[9] However, the Se cathodes also have a dissolution issue associated with high-order lithium selenides (Li_2Se_x, x > 4) and large volume expansion during the charge/discharge process, resulting in a low Se utilization, inferior capacity and short cycle life.[9-11] To the best of our knowledge, there has been no reports of single atom catalyst in Li-Se batteries, where atomic catalysts can maximize the functionalization of selenium cathode hosts to achieve high rate and cycling performance in Li-Se batteries. Therefore, in this work, for the first time, we report an effective Se host with atomically dispersed Co-N-C catalysts in MOF-derived in hierarchical porous carbon matrix for Li-Se batteries. The improvement of electrochemical performance was investigated experimentally and theoretically, which systemically revealed the fundamental of atomic Co-N-C in activating selenium reactivity and immobilizing polyselenides for Li-Se batteries.

Owing to the highly novel design of Co-N-C based host, there are many significant results can be achieved in this work. We demonstrated that single atom catalysts can enable highly effective cathodes for Li-Se batteries with superior rate capability and outstanding long-term cycling

performance. We also achieved an extraordinary high-power Li-Se batteries, delivering a high discharge capacity, a superior rate capability (50 C), and an excellent cycling stability with a Coulombic efficiency of ~100%. This work could open a new avenue for achieving long cycle life and high-power lithium-selenium batteries and provide a universal strategy to synthesize novel cobalt single atoms/nitrogen-doped porous carbon for many other rechargeable batteries.

The understanding of novelty and significance of this work was performed by many advanced instrumental measurement and analysis. For instance, the aberration-corrected high-angle annular dark-field scanning transmission electron microscopy (HAADF-STEM) image and extended X-ray absorption fine structure (EXAFS) data have been provided strong evidence that single Co atoms are homogeneously dispersed within the Co_{SA}-HC particles. The X-Ray absorption near edge structure (XANES) results also revealed that the single Co atoms are positively charged and the EXAFS data shows that isolated single Co atoms can be atomically anchored on the carbon frameworks through the formation of Co-N_3 and Co-N_4 coordination moieties within Co_{SA}-HC particles. To further understand the enhancement of reaction kinetics of charge/discharge of the Se@Co_{SA}-HC cathodes, first-principles calculations were performed to investigate the different possible reactions of lithium polyselenides on nitrogen-doped carbon (NC) as a reference and Co single atoms/nitrogen-doped carbon (Co-NC). Based on the density functional theory (DFT) calculations, it indicates that the reaction rate from the reduction of Li_2Se_2 into Li_2Se is noticeably increased during the discharge process by using single Co atom catalysts. From the charging process data, a smaller value than the calculated energy barriers for Li_2Se decomposition through the single Co atom catalysts can be obtained. Therefore, we proposed a mechanism that single Co atoms can quickly catalyse the transformation from Li_2Se_2 into Li_2Se during the discharging process and the transformation of Li_2Se during the charging process.

In summary, we demonstrated a novel design of highly efficient atomic Co-N-C based porous carbon as a host for high performance Li-Se batteries. This composite exhibits sophisticated features including hierarchical micro-mesopores, positively charged and highly dispersed single Co atoms anchored into the porous carbon frameworks through the formation of Co-N coordination, thereby facilitating the formation kinetics of polyselenides and achieving outstanding performance as a Se host for Li-Se batteries. This novel work could inspire a rational design of atomic catalysts for many other energy storage systems.

References:

1. Y. Li, J. Fan, M. Zheng and Q. F. Dong, *Energy Environ. Sci.*, **2016**, *9*, 1998-2004.
2. Y. Li, J. Fan, J. Zhang, J. Yang, R. Yuan, J. Chang, M. Zheng and Q. Dong, *ACS Nano*, **2017**, *11*, 11417–11424.
3. B. Zhang, T. Sheng, Y. Liu, Y. Wang, L. Zhang, W. Lai, L. Wang, J. Yang, Q. Gu, S. Chou, H. Liu, S. Dou, *Nat. Commun.*, **2018**, *9*, 4082.
4. J. Xu, W. Zhang, Y. Chen, H. Fan, D. Su, and G. Wang, *J. Mater. Chem. A*, **2018**, *6*, 2797-2807
5. Z. B. Cheng, H. Pan, J. Q. Chen, X. P. Meng and R. H. Wang, *Adv. Energy Mater.*, **2019**, *9*, 1901609.
6. Y. Li, G. Chen, J. Mou, Y. Liu, S. Xue, T. Tan, W. Zhong, Q. Deng, T. Li, J. Hu, C. Yang, K. Huang, M. Liu, *Energy Storage Mater.*, **2020**, *28*, 196-204.
7. X. Yang, H. Wang, D. Y. W. Yu, A. L. Rogach, *Adv. Funct. Mater.*, **2018**, *28*, 1706609.
8. Y. Yao, M. Chen, R. Xu, S. Zeng, H. Yang, S. Ye, F. Liu, X. Wu, Y. Yu, *Adv. Mater.*, **2018**, *30*, 1805234.
9. Z. Li, L.Yuan, Z. Yi, Y. Liu, Y. Huang, *Nano Energy*, **2014**, *9*, 229-236.
10. C. P. Yang, S. Xin, Y. X. Yin, H. Ye, J. Zhang and Y. G Guo, *Angew. Chem. Int. Ed.* **2013**, 52, 8363-8367.
11. J. T. Lee, H. Kim, M. Oschatz, D. C. Lee, F. Wu, H. T. Lin, B. Zdyrko, W. Cho S. Kaskel and G. Yushin, *Adv. Energy Mater.*, **2015**, *5*, 1400981.

Original comment 1:

1. In addition to the DFT calculation, the catalyst effect of the cobalt atoms needs to be demonstrated via direct electrochemical experiments. The key kinetic parameters, such as the exchange current density, the diffusion coefficient, should be provided.

Response:

We really appreciate this valuable suggestion. We conducted the direct electrochemical experiments for demonstrating the catalytic effect. The results have been added and discussed in the revised manuscript.

Galvanostatic intermittent titration technique (GITT) testing and EIS spectra were employed to determine key parameters (e.g. exchange current density and the diffusion coefficient) for Li-Se batteries. In this measurement, GITT was carried out with a current pulse of 50 mA g^{-1} for 1h followed by a 3 h relaxation process to achieve a balance state during the charge/discharge processes. The diffusion coefficient (D_{Li}^{+}) was calculated based on the equation:

$$D_{Li+} = \frac{4}{\pi\tau}(\frac{m_B V_M}{M_B A})^2(\frac{\Delta E_s}{\Delta E_\tau})^2(\tau << L^2 / D_{Li+}) \quad (1)$$

Where τ is the duration time of the current pulse, m_B is the mass of the active material, V_M is the molar volume (16.45 cm^3 mol^{-1}), M_B is the molecular weight (78.97 g mol^{-1}) and A is the total contacting area of electrode with electrolyte (1.13 cm^2). ΔE_S is the difference between two consecutive stable voltages after relaxation, ΔE_τ is the transient voltage-change during a single titration step and the L is the thickness of the cathode. The GITT plots of Se@Co_{SA}-HC and Se@HC (**Figure R3a**) and the diffusion coefficients D_{Li}^{+} (**Figure R3b**) of the Se@Co_{SA}-HC calculated from GITT plots were estimated to be 1.02×10^{-13}～1.7×10^{-13}, which is almost ten times that of the Se@HC cathode (1.03×10^{-14}～8.35×10^{-14}) material. This result indicates the better electrochemical kinetics Se@Co_{SA}-HC cathode materials in Li-Se batteries.

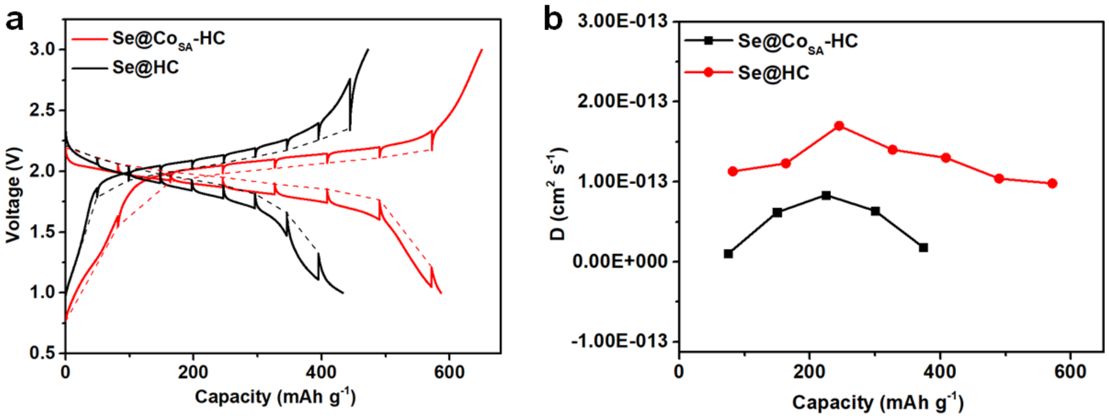

Figure R3. (a) Galvanostatic intermittent titration technique (GITT) testing during the charge/discharge process. (b) The diffusion coefficients D_{Li}^{+} of Se@Co_{SA}-HC and Se@HC cathode calculated from GITT plots.

The exchange current density i_0 was calculated by the Butler–Volmer equation (2) as follows:

$$i_0 = RT / nFAR_{ct} \quad (2)$$

Where R is the gas constant (8.3145 J/mol/K) and T is the absolute temperature. The n is the number of transferred electrons; F is the Faraday constant (96485 C/mol). A is the surface areas

and R_{ct} is charge transfer resistance. According to the Butler–Volmer equation and EIS spectra (**Figure R4**), the exchange current density i_0 of Se@Co_{SA}-HC and Se@HC cathode is 1.14 mA cm^{-2} and 0.76 mA cm^{-2}, respectively. All these key kinetic parameters confirm the catalytic effect of the cobalt single atoms can boost the electrochemical performance for Li-Se batteries.

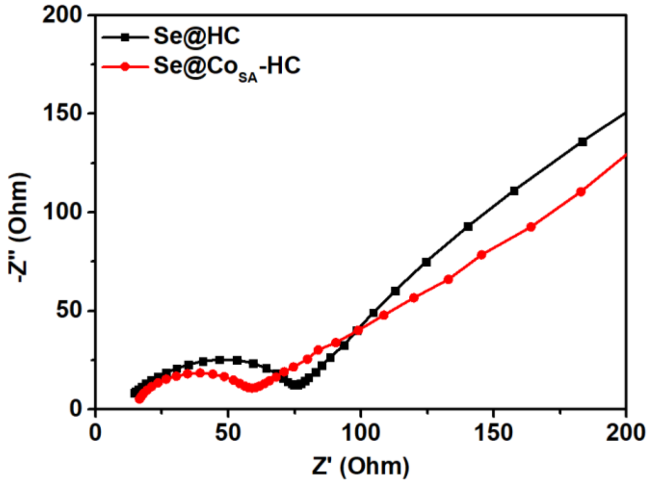

Figure R4. EIS spectra of Se@Co_{SA}-HC and Se@HC at open-circuit potential.

Based on these results, we have further modified our manuscript and added corresponding content in the manuscript on Page 18 and 19. The **Figure R3** and **Figure R4** have been added in the revised supporting information as **Figure S31** and **Figure S32**.

2. The voltage profiles at various rates and cycles should be provided and compared.

Response:

We appreciate this valuable suggestion. Following this suggestion, we have plotted the voltage profiles at various rates and cycles for Se@Co_{SA}-HC, Se@HC and Se@Co_{NP}-HC as shown in **Figure R5**. It indicates that both discharge and charge processes exhibit stable voltage plateaus at around 2.0 V. In addition, the Se@Co_{SA}-HC cathode exhibits superior specific capacities at different rates, which are much higher than those of Se@HC and Se@Co_{NP}-HC cathodes.

The **Figure R5** has been added in the revised supporting information as **Figure S17** on Page 21. The following description has been added in the manuscript on Page 12.

Figure S17 shows the discharge and charge profiles of Se@Co_{SA}-HC, Se@HC and Se@Co_{NP}-HC at various rates and cycles. It indicates that both discharge and charge processes exhibit stable voltage plateaus at around 2.0 V.

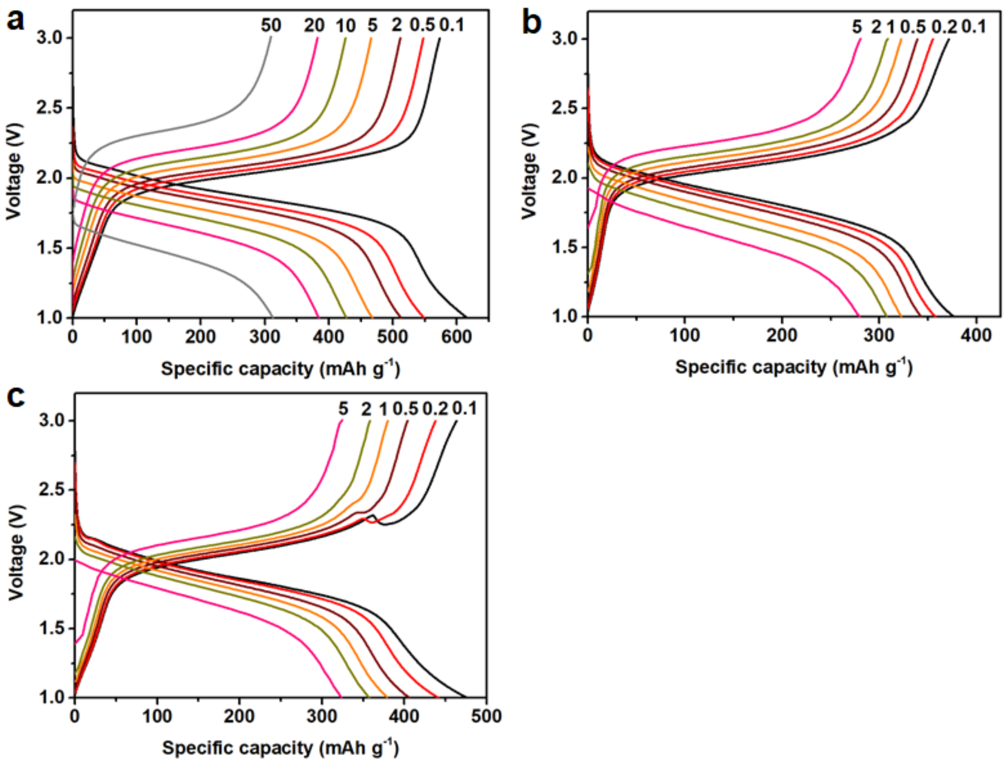

Figure R5. (a) The charge–discharge voltage profiles of Se@Co$_{SA}$-HC at various current densities from 0.1 to 50 C. (b) The charge–discharge voltage profiles of Se@HC at various current densities from 0.1 to 5 C. (c) The charge–discharge voltage profiles of Se@Co$_{NP}$-HC at various current densities from 0.1 to 5 C.

3. To explain the EIS curve, it is proposed that a stable passivation layer forms at the surface of the Se@CoSA-HC particles during the first lithiation process. In the previous reports, all the passivation layers formed in the carbonate electrolyte, while 1,3-dioxolane and 1,2-dimethoxyethane are used as solvents in this work. How the passivation layer form?

Response:

We appreciate the reviewer's valuable suggestions for improving our manuscript. According to these useful comments, the EIS curves, fitted data and explanations have been carefully checked and clarified.

We have conducted electrochemical impedance spectra (EIS) to investigate the fundamental electrochemistry using the equivalent circuit. In Figure S25b, two distinctive depressed semicircles in the EIS spectra are clearly observed at the beginning states of discharge (fresh state and 2.1V discharged state) process for Li-Se batteries. The similar observations in EIS spectra study for Li-Se and Li-S batteries using ether-based electrolytes have also been reported.[1-3] For Li-Se batteries based on ether-based electrolyte, the first semicircle and second semicircle in EIS spectra for Li-Se batteries could be ascribed to the charge transfer and accumulation of electronic/ionic insulating Li_2Se_2/Li_2Se layer on cathode surface, respectively.

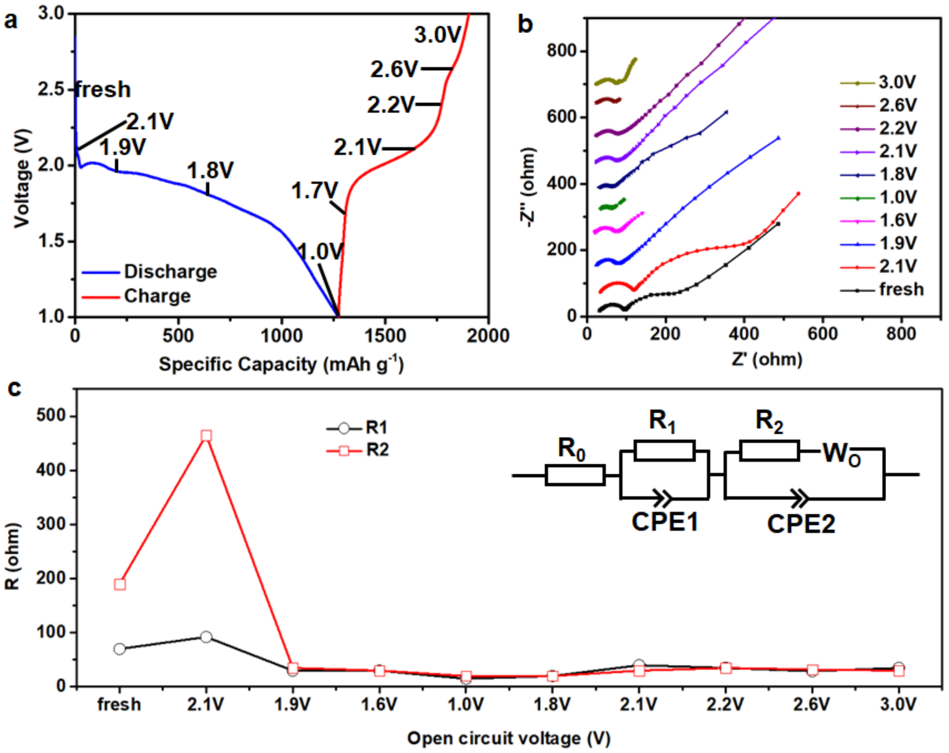

Figure S25. EIS measurements of Se@CoSA-HC composite for the Li-Se battery. (a) a typical discharge and charge profiles at 0.1 C (1st cycle, each point represents an EIS measurement). (b) EIS data at various discharging/charging stages. (c) The ohmic (R_0), charge transfer resistance (R_1), resistance of electronic/ionic insulating layer (R_2) resistances plotted versus various discharging/charging stages and inset is the equivalent circuit for fitting.

We have revised the manuscript and the discussion for EIS spectra on Page 17 and 18 as below:

All the results exhibit two depressed or overlapping semicircles followed by a sloping line. For Li-Se batteries based on ether-based electrolyte, the first semicircle and second semicircle in EIS spectra for Li-Se batteries could be ascribed to the charge transfer and accumulation of electronic/ionic insulating Li_2Se_2/Li_2Se layer on cathode surface, respectively.[56] Combined with the XPS results in **Figure S24**, it is proposed that a stable insulating layer formed at the surface of the Se@Co_{SA}-HC particles during the first lithiation process. Despite the solubility of Se, lithium polyselenides and Li_2Se in ether-based electrolytes[11], side reactions have been restricted when using Se@Co_{SA}-HC composites and the formed Li_xSe may be protected from further reaction by the stable insulating layer. The equivalent circuit for fitting is shown in the inset of **Figure S25c**. In the equivalent circuits, R_0 represents the impedance that is mainly derived from the resistance of the electrolyte, R_1 is the charge transfer resistance at the conductive agent interface, and R_2 is the resistance of electronic/ionic insulating Li_2Se_2/Li_2Se layer.[56] CPE1 represents double-layer capacitance (C_{dl}), while CPE2 (Constant phase element) describes the space charge capacitance of the insulating layer. W_0 is the Warburg impedance corresponding to the polyselenide diffusion processes. The resistance values (R_1 and R_2) obtained from **Figure S25b** are summarized in **Figure S25c**. **Figure S26** shows the typical Nyquist plots collected at different discharge-charge state and their fitted curves with two depressed or overlapping semicircles. The obtained resistance values are presented in **Table S3**. The R_1 value is relatively stable throughout the whole cycle process, indicating the excellent charge transfer capability.

References:

1. X. Wang, Y. Tan, Z. Liu, Y. Fan, M. Li, H. A. Younus, J. Duan, H. Deng, S. Zhang, *Small*, **2020**, *16*, 2000266.
2. L. Yuan, X. Qiu, L. Chen, W. Zhu, *J. Power Sources*, **2009**, *189*, 127-132.

3. N. A. Cañas, K. Hirose, B. Pascucci, N. Wagner, K. A. Friedrich, R. Hiesgen, *Electrochim. Acta*, **2013**, *97*, 42-51.
11. A. Abouimrane, D. Dambournet, K. W. Chapman, P. J. Chupas, W. Weng, K. Amine. *J. Am. Chem. Soc.* **2012**, *134*, 4505-4508.
56. X. Wang, Y. Tan, Z. Liu, Y. Fan, M. Li, H. A. Younus, J. Duan, H. Deng, S. Zhang, *Small*, **2020**, 16, 2000266.

4. With the increase of the rates, the relative ratio of capacitive contribution to the total capacity increases. Is it related with the cobalt catalyst?

Response:

The relative ratio capacitive contribution of can be quantitatively differentiated by separating current response *i* at a fixed potential *V* into a surface dependent process (proportional to ν) and a diffusion-controlled process (proportional to $\nu^{1/2}$) by the follow equation (3) [1,2]

$$i = k_1\nu + k_1\nu^{1/2} \qquad (3)$$

Where ν is the scan rate, $k_1\nu$ corresponds to capacitive effects and $k_1\nu^{1/2}$ corresponds to diffusion-controlled processes. Through determining k_1 and k_2, we can separate the fraction of the two processes. The capacitive contribution is related to the current response at different scan rates.

It is well-known that the ratio of capacitive contribution benefits the high-power capability for rechargeable batteries.[3] In this work, cobalt single atoms/nitrogen-doped hollow porous carbon (Co_{SA}-HC) not only can be a host for Se, but also can supply active surface sites to store Li^+, especially at a high rate. In particular, compare with the Se@HC and Se@Co_{NP}-HC, the Se@Co_{SA}-HC cathode demonstrates a higher rate performance (50C) due to the addition of single Co atoms. Through DFT calculation as shown in **Figure 5** in the manuscript (see the figure below), we found that single Co atoms can quickly catalyse the transformation from Li_2Se_2 into Li_2Se during the discharging process and the decomposition of Li_2Se during the charging process. The single cobalt nanoparticles are serving as active sites to enhance kinetics for Li^+ ions storage in Li-Se batteries. At the same time, the atomic cobalt on the Co-NC support could effectively alleviate the dissolution of polyselenides, electro-catalyse the transformation from polyselenides to Li_2Se and minimize the reaction energy barriers, leading to the high-power Li-Se batteries.

Therefore, the single cobalt catalyst is related with the capacitive contribution and also optimizes the features of porous carbon materials towards activation of selenium reactivity and immobilization of selenium and polyselenides.

References:

1. V. Augustyn, J. Come, M. A. Lowe, J. W. Kim, P. Taberna, S. H. Tolbert, H. D. Abruña, P. Simon and B. Dunn, *Nat. Mater.* **2013**, *12*, 518.
2. T. Brezesinski, J. Wang, S. H. Tolbert and B. Dunn. *Nat. Mater.* **2010**, *9*, 146-151.
3. P. Lu, Y. Sun, H. Xiang, X. Liang, Y. Yu, *Adv. Energy Mater.* **2018**, *8*, 1702434.

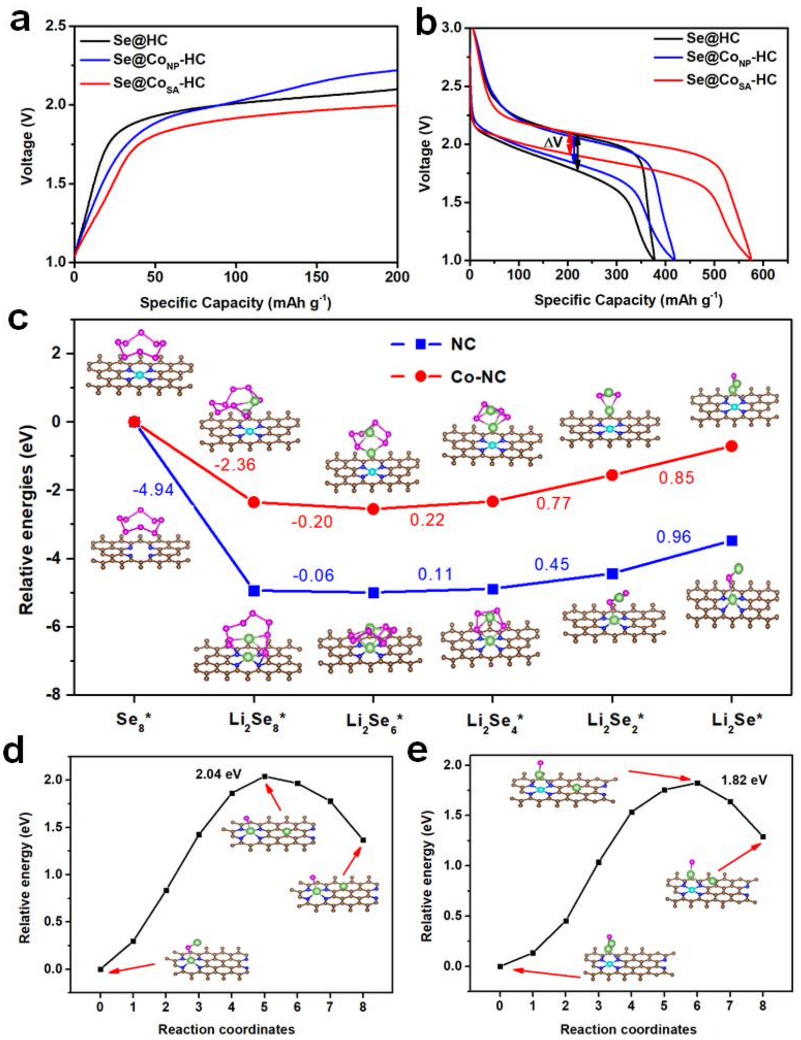

Figure 5. Catalytic effects of Co_{SA}-HC particles for Li-Se batteries. (a) The first cycle charge profiles at 0.1C and (b) discharge-charge profile at 0.1C of Se@Co_{SA}-HC, Se@HC, and Se@Co_{NP}-HC. (c) Energy profiles for the reduction of lithium polyselenides on NC and Co-NC supports (insets: the optimized adsorption conformations of intermediate species on NC and Co-NC substrate). Energy profiles of the decomposition of Li_2Se clusters on NC (d) and Co-NC (e). (insets: the initial, transition, and final structures.) The grey, pink, green, blue, and cyan balls represent C, S, Li, N, and Co atoms, respectively.

5. *The fitted curve should be provided in the EIS analysis.*

Response:

According to review's comment, we have fitted the curves of EIS to clearly present the electrochemical analysis. **Figure R6** shows typical Nyquist plots collected at different discharge-charge state and their fitted curves. All the results exhibit two depressed or overlapping semicircles followed by a sloping line. For Li-Se batteries based on ether-based electrolyte, the first semicircle and second semicircle in EIS spectra for Li-Se batteries could be ascribed to the charge transfer and accumulation of electronic/ionic insulating Li_2Se_2/Li_2Se layer on cathode surface, respectively, which was also reported in previous work for Li-Se batteries using ether-based electrolytes.[1] Despite the solubility of Se, lithium polyselenides and Li_2Se in ether-based electrolytes[2], side reactions seem restricted when using Se@Co_{SA}-HC composites and the formed Li_xSe may be protected from further reaction by the stable insulating layer.

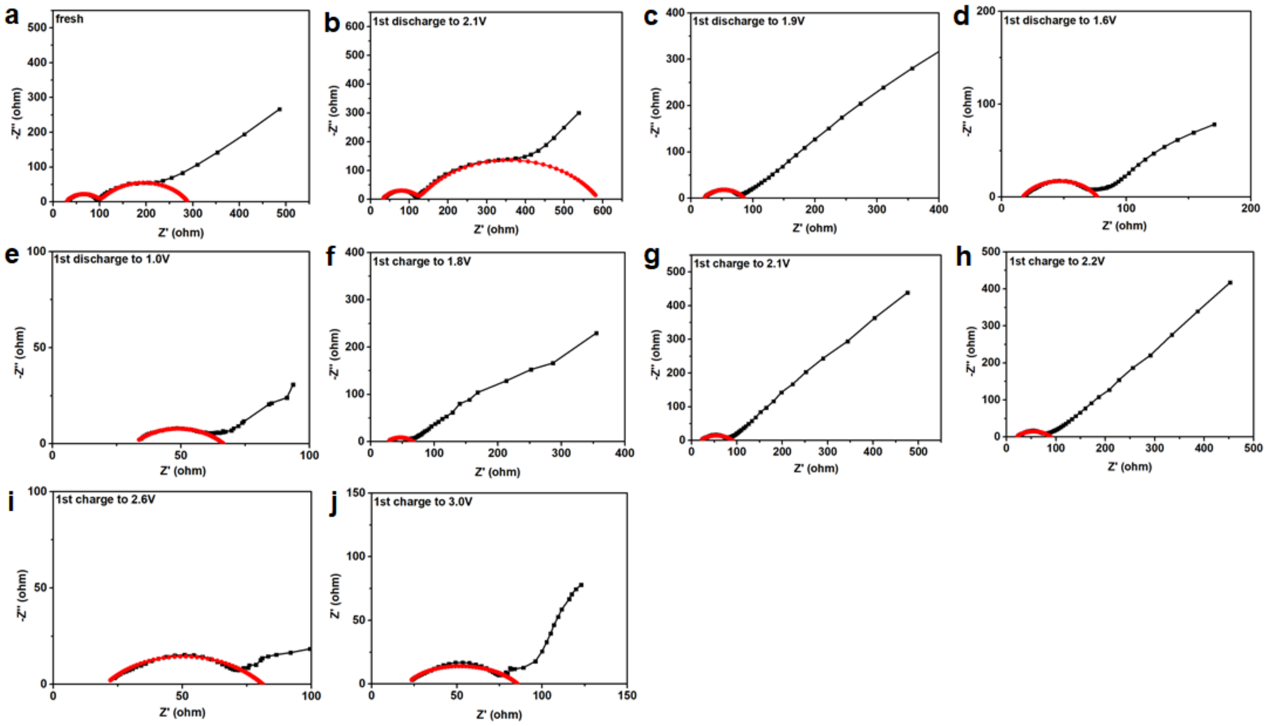

Figure R6. Typical Nyquist plots collected at (a) fresh, (b) 1st discharge to 2.1 V, (c) 1st discharge to 1.9 V, (d) 1st discharge to 1.6 V, (e) 1st discharge to 1.0 V, (f) 1st charge to 1.8 V, (g) 1st charge to 2.1 V, (h) 1st charge to 2.2 V, (i) 1st charge to 2.6 V and (j) 1st charge to 3.0 V.

Table R1. The resistance values obtained from the typical Nyquist plots collected at different discharge-charge state.

Number	State	R_0 (ohm)	R_1(ohm)	R_2(ohm)
1	fresh	29	70	190
2	1st discharge to 2.1 V	32	92.15	465
3	1st discharge to 1.9 V	21	30	35
4	1st discharge to 1.6 V	18	30	30
5	1st discharge to 1.0 V	30	15	20
6	1st charge to 1.8 V	27	20	20
7	1st charge to 2.1 V	20	40	30
8	1st charge to 2.2 V	20	35	35
9	1st charge to 2.6 V	20	29	32
10	1st charge to 3.0 V	21	35	30

Based on the above description, we have revised the manuscript accordingly. The **Figure R6** has been added in the revised supporting information as **Figure S26**. The **Table R1** has been added in the revised supporting information as **Table S3**. The description below has been added in the revised manuscript on Page 17.

Figure S26 shows the typical Nyquist plots collected at different discharge-charge state and their fitted curves with two depressed or overlapping semicircles. The obtained resistance values are presented in **Table S3**.

References:

1. X. Wang, Y. Tan, Z. Liu, Y. Fan, M. Li, H. A. Younus, J. Duan, H. Deng, S. Zhang, *Small*, **2020**, *16*, 2000266.
2. A. Abouimrane, D. Dambournet, K. W. Chapman, P. J. Chupas, W. Weng, K. Amine. *J. Am. Chem. Soc.* **2012**, *134*, 4505-4508.

6. *In figure 5, the ΔV should be pointed.*

Response:

We would like to thank the reviewer's valuable suggestion. We have labelled ΔV (lowest voltage hysteresis) of three samples **Figure R7**. Se@Co_{SA}-HC cathodes exhibit smaller voltage hysteresis (188 mV) than Se@HC (295 mV) and Se@Co_{NP}-HC cathodes (234 mV). Accordingly, the **Figure 5b** in the manuscript has been revised.

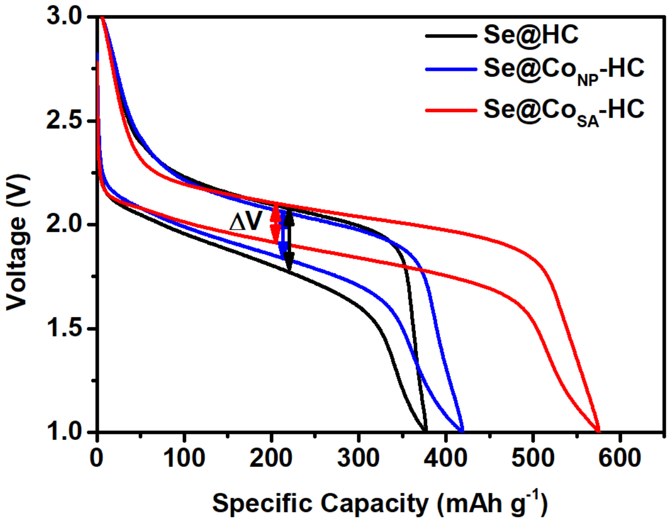

Figure R7. discharge-charge profile at 0.1C of Se@Co_{SA}-HC, Se@HC, and Se@Co_{NP}-HC.

7. *The areal loading of selenium in the cathode should be provided in the experimental part.*

Response:

The areal loading of selenium in the cathode is 0.8 mg/cm^2. In the revised manuscript, we have added the description sentence in the experimental section of manuscript on Page 26.

Responses to Reviewer #3

Reviewer Letter:

The MS is suitable for publication in Nat Comm, I have the following concerns.

1. In this study, authors have prepared a ZnCo-ZIF, which basically is a MOF, and then pyrolyzed it to obtain porous spheres which have been labeled as Co-HC. The disappearance of Zn from the structure is not clear to me- Zn^{2+} ions cannot get pyrolyzed and vanish, as Scheme 1 seems to suggest- if there were any steps, after the PS-ZIF formation and before the Co-HC formation, please include them. Otherwise, it looks scientifically incorrect.

Response:

We are grateful to reviewer #3 for his/her time and valuable suggestions to improve our manuscript. The comments are positive and encouraging. All concerns of the reviewer have been addressed carefully. Following his/her suggestions, we have further revised our manuscript.

According to our previous work [1], it was observed that zinc can be reduced and evaporated during the calcination process. To identify the evaporation of zinc, thermogravimetric analysis–mass spectrometry (TGA-MS) was used to monitor the gas products during the calcination. MS measurement was only used to qualitatively trace the volatile species. As shown in **Figure R8** below, it is clearly to see that zinc evaporates and CO_2 were detected from TG-MS profile, indicating the zinc evaporation and the release of CO_2. It was also reported that the evaporation of the zinc during the pyrolysis process. [2-4]

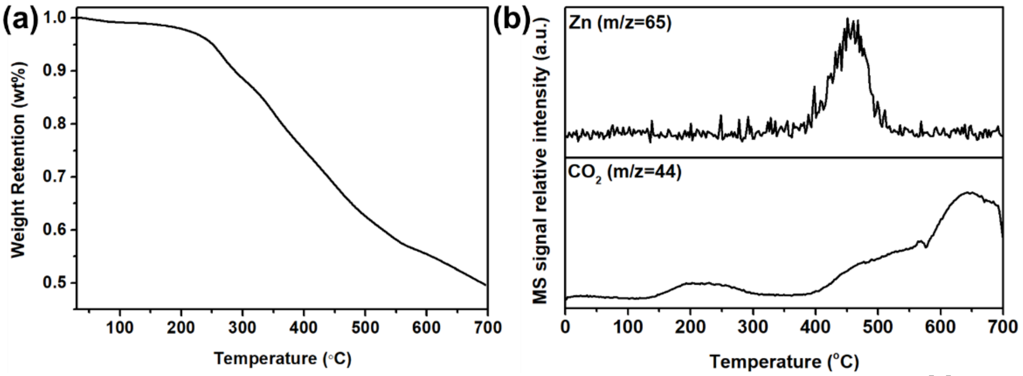

Figure R8. Thermal behaviour of ZnO@polymer: (a) TG curves; (b) MS profiles.[1]

The Scheme 1 has been revised as shown below. The following statement has been added in the revised manuscript on Page 4.

"More importantly, the core-shell ZIF hybrid structure can be further converted into hollow structured carbon materials via a pyrolysis process with zinc evaporation."

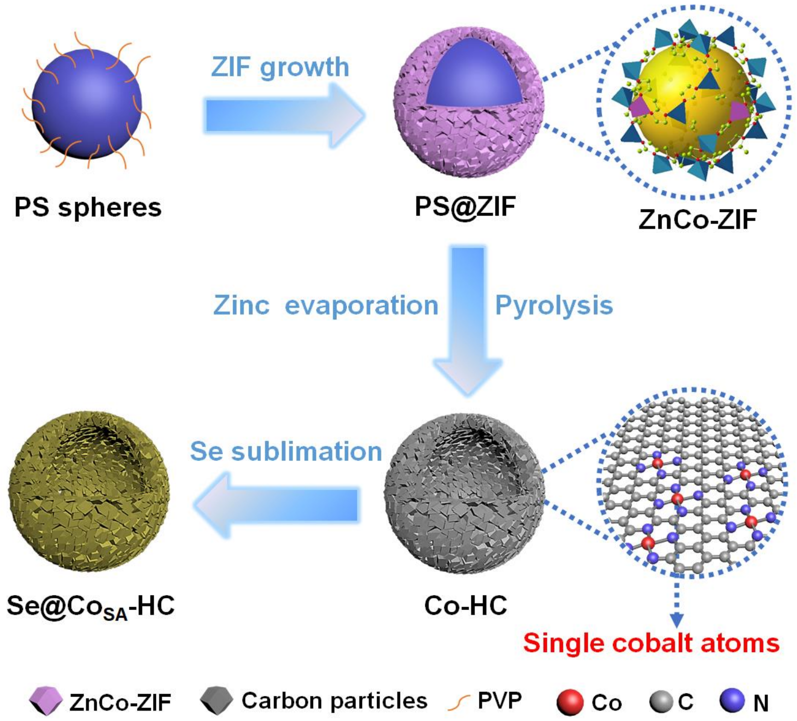

Scheme 1. Schematic illustration of the procedures for synthesising cobalt single atoms/nitrogen-doped hollow porous carbon (Co$_{SA}$-HC) particles.

References:

1. H. Tian, F. Huang, Y. H. Zhu, S. M. Liu, Y. Han, M. Jaroniec, Q. H. Yang, H. Y. Liu, G. Q. Max Lu, and J. Liu, *Adv. Funct. Mater*. **2018**, *28*,1801737.
2. H. Yang, S. J. Bradley, A. Chan, G. I. N. Waterhouse, T. Nann, P. E. Kruger, S. G. Telfer, *J. Am. Chem. Soc.* **2016**, *138*, 11872.
3. X. Wang, Z. Chen, X. Zhao, T. Yao, W. Chen, R. You, C. Zhao, G. Wu, J. Wang, W. Huang, J. Yang, X. Hong, S. Wei, Y. Wu, Y. Li, *Angew. Chem. Int. Ed.* **2018**, *57*, 1944.
4. P. Yin, T. Yao, Y. Wu, L. Zheng, Y. Lin, W. Liu, H. Ju, J. Zhu, X. Hong, Z. Deng, G. Zhou, S. Wei, Y. Li, *Angew. Chem. Int. Ed.* **2016**, *55*, 10800.

2. The title of this manuscript is also extremely difficult to come to terms with. Please change it to ZnCo or Co-hollow porous carbon composite with Se (which is what it is!). In general, everything is made of atoms, so just magnifying the hollow ZnCo-HC using a microscope, and labelling the atoms, does not imply that Selenium's charge storage capacity is enhanced by the Cobalt atoms, this would have been the case if they were elemental Co stand-alone atoms, but as it stands, in this MS, they are not stand-alone entities, but are very much a part of a large porous matrix, and are covalently linked to other elements like carbon and nitrogen. Further to strengthen my line of reasoning, I would like to direct your attention to the XPS core level spectrum of Co2p. Co does not exist in the fully reduced state as elemental Co, it does in Co2+ and Co3+ states, as shown therein. Whats' the third peak due to?

Response:

Following the reviewer's comment, we have revised the title as "High-power Lithium-selenium Batteries Enabled by Atomic Cobalt Electrocatalyst Incorporated in Hollow Carbon Cathode."
As shown in the Co 2p XPS spectra of Co$_{SA}$-HC particles (**Figure R9**), the third peak at 784.6 and 802.8 eV are satellite peaks that can be ascribed to the shakeup excitation of the high-spin Co^{2+} ions.[1]

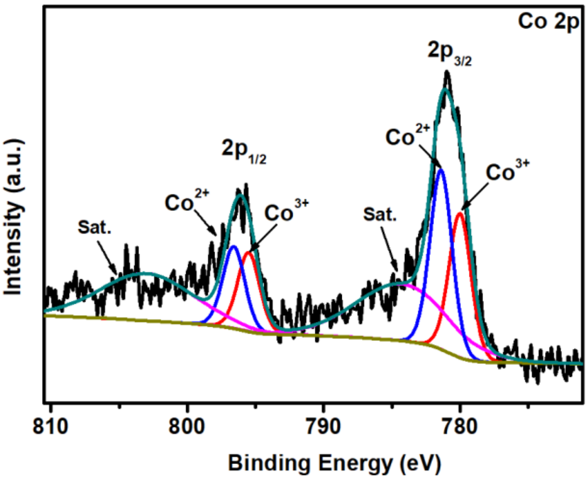

Figure R9. Co 2p XPS spectra of Co_{SA}-HC particles.

Accordingly, the **Figure 2** has been revised. The following sentence has been added in the revised manuscript on Page 10 and Page 11.

"The peaks at 784.6 and 802.8 eV are satellite peaks that can be ascribed to the shakeup excitation of the high-spin Co^{2+} ions.[1]"

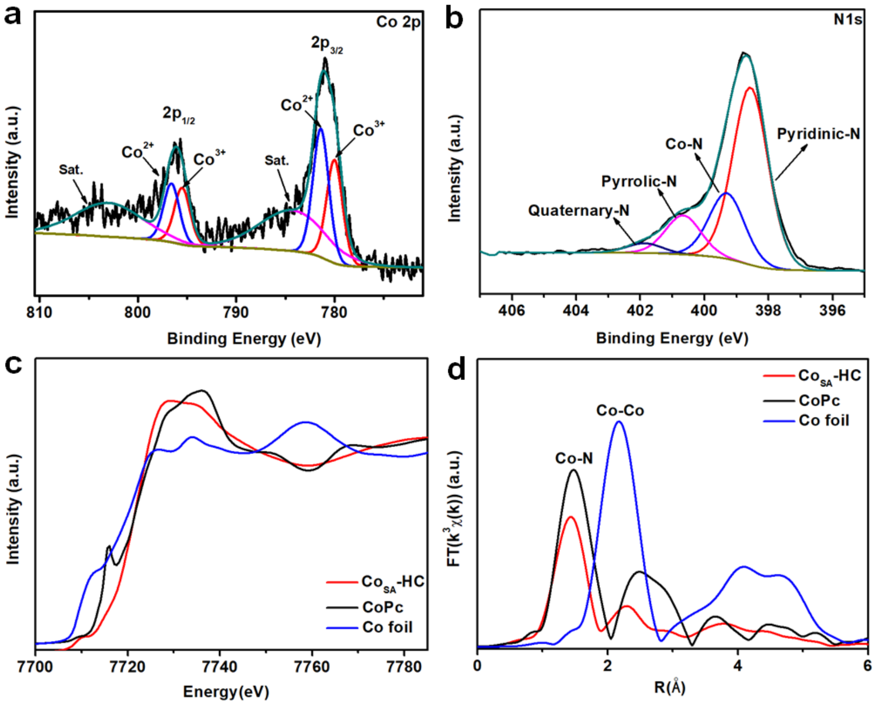

Figure 2. (a) Co 2p and (b) N 1s XPS spectra of Co_{SA}-HC particles, (c) Co XANES spectra and (d) Fourier-transform EXAFS spectra of Co_{SA}-HC, Co foil and Co phthalocyanine (CoPc).

Reference:

1. H. Tian, X. Liu, L. Dong, X. Ren, H. Liu, C. A. H. Price, Y. Li, G. Wang, Q. H. Yang, J. Liu, *Adv. Sci.* **2019**, *6*, 1900807.

3. Figure 3: Can you show the CV curves after 10th and 100th cycle?

Response:

We have followed the reviewer's suggestion and added the CV curves after 10th and 100th cycles in the revised manuscript.

We assembled cells based on Se@Co_{SA}-HC cathodes to measure the CV curves at a scan rate of 2.0 mV/s up to 100 cycles. The CV curves after 10th and 100th cycle are illustrated in **Figure R10**. The CV curves after 10th and 100th cycles at a higher scan rate of 2.0 mV/s overlap very well, which is consistent with the cycling performance of the Se@Co_{SA}-HC cathodes as illustrated in **Figure 3** in the manuscript and **Figure S22** in the supporting information.

Based on these results, we have further modified our manuscript. The **Figure R10** has been added in the revised supporting information as **Figure S15**. The following sentence has been added in the revised manuscript on Page 12.

"As illustrated in **Figure S15**, the CV curves after 10th and 100th cycle overlap very well at a higher scan rate of 2.0 mV/s."

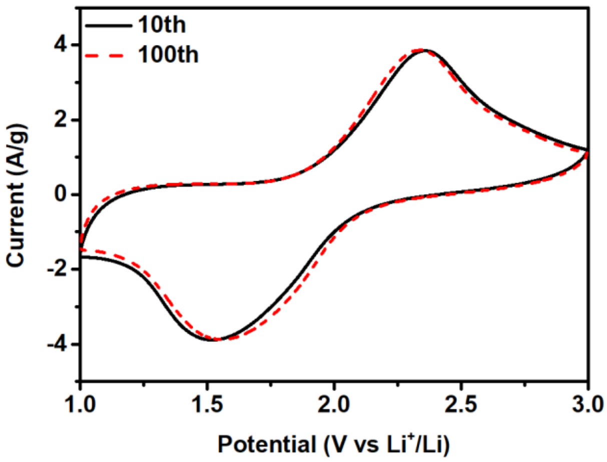

Figure R10. CV curves of Se@Co_{SA}-HC at a scan rate of 2.0 mV/s after 10th and 100th cycle.

4. *Why does Se becomes less crystalline once the composite is formed?*

Response:

The porosity of the Co_{SA}-HC particles was studied by nitrogen adsorption-desorption analysis. The Co_{SA}-HC displays mixed type I and IV isotherms (**Figure S9a**). The sharp adsorption increase in the low-pressure region indicates that the existence of abundant micropores and the hysteresis loop in the medium-pressure region originates from the mesopores with the carbon framework. The pore size distribution of Co_{SA}-HC particles was then analysed based on the isotherms (**Figure S9b**), which shows peaks centred at 1, 17 and 35 nm.

The selenium encapsulation into carbon particles was carried out at 300 °C under Ar atmosphere to promote the infusion of Se into the microporous carbon clusters. After the selenization process, the crystallinity of selenium is low, which can be attributed to the transformation of trigonal Se to amorphous Se with the formation of low ordering of selenium and successful confinement in the carbon matrix.[1-3]

Based on the above description, we have revised the manuscript. The following sentence has been added in the revised manuscript on Page 9.

After the selenization process, the crystallinity of selenium is low, which can be attributed to the transformation of trigonal Se to amorphous Se with the formation of low ordering of selenium and successful confinement in the carbon matrix.[1-3]

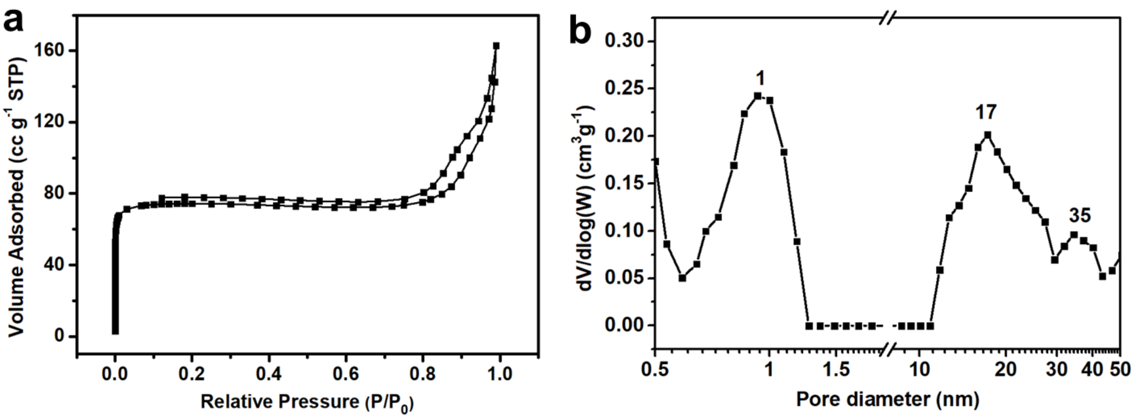

Figure S9. N_2 adsorption–desorption isotherms (a) and the corresponding pore size distribution (b) of Co_{SA}-HC particles.

References:

1. Z. Li, L. Yuan, Z. Yi, Y. Liu and Y. H. Huang, *Nano Energy*, **2014**, *9*, 229-236.
2. C. P. Yang, S. Xin, Y. X. Yin, H. Ye, J. Zhang and Y. G. Guo, *Angew. Chem. Int. Ed.*, **2013**, *52*, 8363-8367.
3. J. T. Lee, H. Kim, M. Oschatz, D. C. Lee, F. Wu, H. T. Lin, B. Zdyrko, W. Cho S. Kaskel and G. Yushin, *Adv. Energy Mater.*, **2015**, *5*, 1400981.

Reviewer #1 (Remarks to the Author):

The authors revised the manuscript carefully, I recommend acceptation of this revised manuscript.

Reviewer #2 (Remarks to the Author):

The revised MS has been largely improved. I agree that the design of the Co-N-C based host is novel, and many advanced instrumental measurement and analysis have been conducted, but I still do not think the novelty can measure up to Nature Communications.

1. In the revised MS, the areal loading of the energy-bearing materials has been added. The areal loading of selenium in the cathode is 0.8 mg/cm2, and the electrolyte dosage is 20 μL for each coin cell. The Se loading is very low and the E/Se retio is quite high, that decreases the Convince strength of the high performance.

2. The mechanism is still not clear. In the original MS, the authors mentioned SEI forms on the surface of the cathode; in the revised version, the second semicircle in EIS spectra for Li-Se batteries was ascribed to the charge transfer and accumulation of electronic/ionic insulating Li2Se2/Li2Se layer on cathode surface. In either case, how do the catalytic sites keep active in the long cycles? Dose the insulating Li2Se2/Li2Se layer have any influence on the catalytic sites?

Response Letter

Responses to Reviewer #1

Reviewer Letter:

The authors revised the manuscript carefully, I recommend acceptation of this revised manuscript.

Response:

We appreciate the reviewer’s positive comments on our work. We sincerely thank the reviewer once again for the time and efforts to improve the quality of our work.

Responses to Reviewer #2

The revised MS has been largely improved. I agree that the design of the Co-N-C based host is novel, and many advanced instrumental measurement and analysis have been conducted, but I still do not think the novelty can measure up to Nature Communications.

Response:

We would like to thank the reviewer for valuable comments to improve our manuscript. Following these suggestions, we have conducted further experimental investigations and added more detail and deep discussions to improve our manuscript as follows.

1. Electrochemical measurement of high areal loading selenium cathode with low dosage of electrolyte, as per the reviewer requested.
2. Further DFT investigation on Li-Se bond length of lithium polyselenides on different supports to better understand the mechanism.
3. TEM observation on Li_2Se_2/ Li_2Se layer and single cobalt catalysts after long cycling.
4. A visual observation to confirm the electrocatalytic reactivity of single atom catalyst after cycling.

In the revised manuscript and supporting information, all reviewers' comments have been addressed by adding updated information. The supplementary contents have been highlighted by yellow and underlined in the revised manuscript and supporting information, as well as presented in the response letter.

We would like to clarify the reviewer's concern regarding novelty and creativity of our work in the following content. Single-atom catalysis is an innovative and leading-edge research topic in the area of energy storage and conversion. This is evidenced that many high impact papers have been published in Nature and Science.[1-5] For example, Zelenay *et al.* synthesized an iron-nitrogen-carbon catalyst with the formation of FeN_4 active sites, which showed comparable electrocatalytic properties compared with state-of-the-art Pt/C.[1] Zheng *et al.* reported the preparation of ultrathin titanium oxide with high dispersion of atomic palladium (Pd_1/TiO_2), which presented great improvement for catalytic activity in hydrogenation and excellent stability compared to commercial Pd catalysts.[2] Ma *et al.* prepared platinum (Pt) atomically dispersed on α-molybdenum carbide (α-MoC) for hydrogen production with exceptional turnover frequency through aqueous-phase reforming of methanol at low temperatures.[5] Based on the literature review of single-atom catalysts and Li-Se batteries, to the best of our knowledge, there has been no report on investigating catalytic effect of single atoms in Li-Se batteries. Therefore, it is crucial to systemically investigate the mechanism of single-atom catalytic effect in Li-Se batteries, both experimentally and theoretically.

In this paper, for the first time, we discovered that **single atoms can significantly boost electrochemical performances of Li-Se batteries**, including **high rate capacity** and **extended cycle life**. Therefore, the content of this paper is cutting-edge. The significant innovations of this manuscript are highlighted below:

1) For the first time, we demonstrate that single-atom catalysts can enable highly effective cathodes for Li-Se batteries with superior rate capability and outstanding long-term cycling performance.
2) An extraordinary high-power Li-Se battery has been achieved through a single-atom catalysis method.
3) A simple synthesis approach has been developed to synthesize hollow structured particles with micro-mesopores loaded with isolated and highly dispersed single Co atoms. Those single Co

atoms are atomically anchored on the carbon frameworks through the coordination chemistry of Co-N_3 and Co-N_4.

4) The cobalt single atoms can enhance selenium reactivity and improve immobilization of selenium and polyselenides.

This work could open a new avenue for achieving long cycle life and high-power lithium-selenium batteries and provide a strategy to synthesize novel cobalt single atoms/nitrogen-doped hollow porous carbon (Co_{SA}-HC) for other rechargeable batteries. Therefore, this research is innovative.

References:

1. Jones, J., *et al.* Thermally stable single-atom platinum-on-ceria catalysts via atom trapping. Science 353, 150-154 (2016).
2. Liu, P., *et al.* Photochemical route for synthesizing atomically dispersed palladium catalysts. Science 352, 797-800 (2016).
3. Malta, G., *et al.* Identification of single-site gold catalysis in acetylene hydrochlorination. Science 355, 1399-1403 (2017).
4. Chung, H. T., *et al.* Direct atomic-level insight into the active sites of a high-performance PGM-free ORR catalyst. Science 357, 479-484 (2017).
5. Lin, L., *et al.* Low-temperature hydrogen production from water and methanol using Pt/α-MoC catalysts. Nature 544, 80-83 (2017).

1. The areal loading of selenium in the cathode is 0.8 mg/cm2, and the electrolyte dosage is 20 μL for each coin cell. The Se loading is very low and the E/Se retio is quite high, that decreases the Convince strength of the high performance.

Response:

We sincerely appreciate this valuable suggestion. Following this comment, we have prepared Li-Se batteries with high area loading of selenium and low dosage of electrolyte. To increase the Se loading and decrease the electrolyte/Se ratio, the areal loading of selenium in the cathode is about 5 mg/cm^2 and only 5 μL electrolyte was added in each coin cell. **Figure R1** below shows the cycling performance of the Se@Co_{SA}-HC with a high areal loading of selenium about 5 mg/cm^2 at 0.2 C for 100 cycles and the Se@Co_{SA}-HC cathodes can deliver 325 mA h g^{-1} after cycling, indicating stable cyclability and high capacities. According to the literature review, this superior cycling performance with a high areal loading of selenium is among the best of those previously reported Li-Se batteries.[1-4]

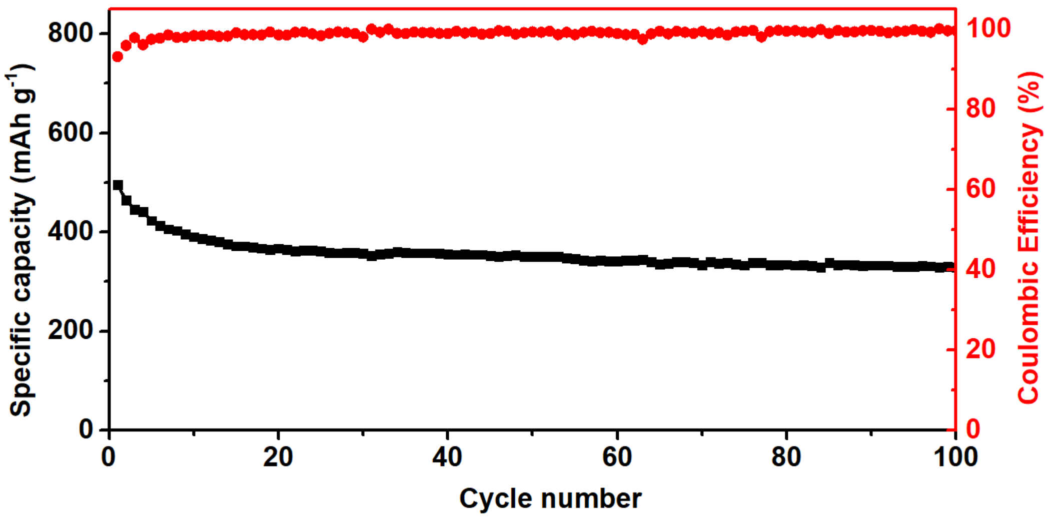

Figure R1 (Figure S22). Cycling performance and Coulombic efficiency for Se@Co_{SA}-HC with areal loading of selenium about 5 mg/cm^2 and low dosage of 5 μL electrolyte per CR2032 coin-cell at 0.2 C for 100 cycles.

The **Figure R1** has been added in the revised supporting information as **Figure S21** on Page 26 in the revised Supporting Information. The following description has been added in the manuscript on Page 11.

Furthermore, **Figure S21** shows the cycling performance of the Se@Co_{SA}-HC cathode with a high areal loading of selenium about 5 mg cm^{-2} at 0.2 C for 100 cycles, demonstrating stable cyclability and high capacities.

References:

1. Fang, R., *et al.* Supercritical CO_2 Assisted Synthesis of 3D Porous SiOC/Se Cathode for Ultrahigh Areal Capacity and Long Cycle Life Li-Se Batteries. J. Mater. Chem. A 6, 24773-24782 (2018).
2. Park, G., *et al.* Carbon Microspheres with Well-developed Micro and Mesopores as Excellent Selenium Host Materials for Lithium–Selenium Batteries with Superior Performances. J. Mater. Chem. A 6, 21410-21418 (2018).
3. Dai, F., *et al.* Hierarchical Electrode Architectures for High Energy Lithium-chalcogen Rechargeable Batteries. Nano Energy 51, 668–679, (2018).
4. Zhao, X., *et al.* Heteroatoms Dual-doped Hierarchical Porous Carbon-selenium Composite for Durable Li–Se and Na–Se Batteries. Nano Energy 49, 137–146, (2018).

2. The mechanism is still not clear. In the original MS, the authors mentioned SEI forms on the surface of the cathode; in the revised version, the second semicircle in EIS spectra for Li-Se batteries was ascribed to the charge transfer and accumulation of electronic/ionic insulating Li2Se2/Li2Se layer on cathode surface. In either case, how do the catalytic sites keep active in the long cycles? Dose the insulating Li2Se2/Li2Se layer have any influence on the catalytic sites?

Response:
We appreciate the reviewer's valuable comments for improving our manuscript. Following the reviewer's comments, we further elucidate the mechanism below.

a) The mechanism of single cobalt catalysts for Li-Se batteries can be explained as follows.

To further understand the enhancement of reaction kinetics of charge/discharge of the Se@Co_{SA}-HC cathodes, first-principles calculations were performed to investigate the different possible reactions of lithium polyselenides on nitrogen-doped carbon support (NC) as a reference and atomic Co/nitrogen-doped carbon supports (Co-NC). As shown in **Figure S37**, two models of nitrogen-doped carbon without and with Co atoms were proposed in our simulation. The reversible overall reaction for the formation of Li_2Se originating from Se_8 and Li was considered.[1] During discharge, the first step involves the reduction of Se_8 and the generation of Li_2Se_8, followed by further reduction and disproportionation with the formation of three intermediate lithium polyselenides, namely, Li_2Se_6, Li_2Se_4, and Li_2Se_2, achieving the formation of Li_2Se as the final product.[1] The Gibbs free energies were calculated for the above reactions on both NC and Co-NC supports (**Table S4**). The optimized structures of the intermediates and their Gibbs free energy profiles are displayed in **Figure 5c**. It can be observed that the transformations from Se_8 to Li_2Se_6 are exothermic and the following three steps involving the conversion of Li_2Se_4, Li_2Se_2, and Li_2Se are endothermic. The largest positive Gibbs free energy can be found in the conversion process from Li_2Se_2 to Li_2Se, revealing its role as the rate-determining step in the whole discharge process. The Gibbs free energy of Co-NC support (0.85 eV) is much lower than that of NC support (0.96 eV), indicating that the

reduction of Se is thermodynamically more favourable on Co-NC than on NC support. In the charging process, the transformation of Li_2Se is the first step.[2] Through the climbing-image nudged elastic band method, the transformation energy and barrier of Li_2Se were calculated to evaluate the delithiation reaction kinetics from Li_2Se to selenium on the surfaces of Co-NC and NC supports. **Figure 5d** and **5e** show the energy profiles for the transformation processes on both Co-NC and NC supports. The calculated energy barriers of Li_2Se transformation of Co-NC supports (1.82 eV) is smaller than that of NC (2.04 eV), revealing that atomic cobalt nanoparticles are serving as active sites to enhance the phase transformation of Li_2Se and the Se utilization in Li-Se batteries. In addition, according to the DFT calculations results shown in **Table R1**, the Li-Se bond length on the surface of Co-NC support is elongated, revealing the interaction between Li atom and selenium atom is weakened. This leads to the easier delithiation for lithium polyselenides, confirming the role of single Co atom catalysts. Therefore, we proposed a mechanism that single Co atoms can quickly catalyse the transformation from Li_2Se_2 into Li_2Se during the discharging process and the decomposition of Li_2Se during the charging process. To demonstrate the mechanism, the schematic illustration of the reaction mechanisms for the Se@Co_{SA}-HC cathodes is clearly shown in **Figure R2**. The confined polyselenides within the carbon framework could be fully reduced into Li_2Se through the catalysis by atomic Co, leading to high Se utilization. Therefore, the single-atom Co in Se@Co_{SA}-HC cathodes plays a significant role in achieving long cycling stability and high reversible capacity.

Table R1 has been added in the revised supporting information as **Table S5** on Page 48. The **Figure R2** has been added in the revised supporting information as **Figure S38** on Page 43. The following description has been added in the manuscript on Page 17.

In addition, from the DFT calculation results shown in **Table S5**, the Li-Se bond length on the surface of Co-NC support is elongated, revealing the interaction between Li atom and the rest of the molecule is weakened. This leads to the easier delithiation for lithium polyselenides, confirming the role of single Co atom catalysts. Therefore, we proposed a mechanism that single Co atoms can quickly catalyse the transformation from Li_2Se_2 into Li_2Se during the discharging process and the transformation of Li_2Se during the charging process. To demonstrate the mechanism, the schematic illustration of electrode reaction mechanisms for the Se@Co_{SA}-HC cathodes is shown in **Figure S38**. The confined polyselenides within the carbon framework could be fully reduced into Li_2Se by single atom Co catalysts, leading to high Se utilization.

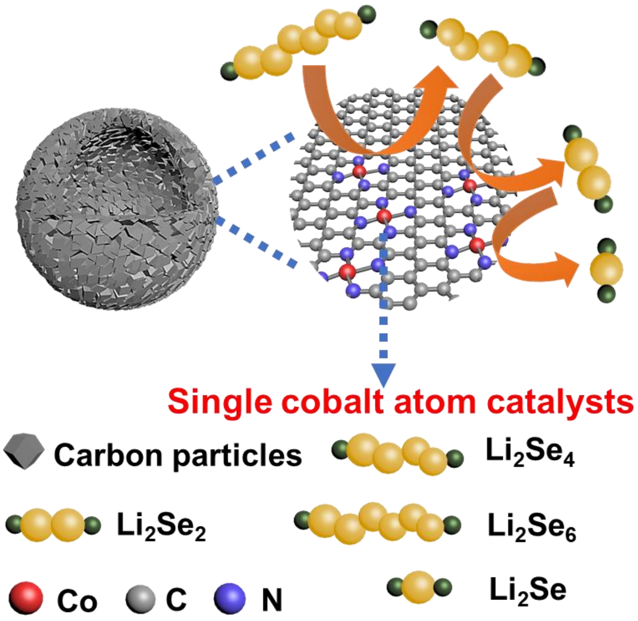

Figure R2 (Figure S38). Schematic illustrations of the electrode reaction mechanism of Se@Co_{SA}-HC.

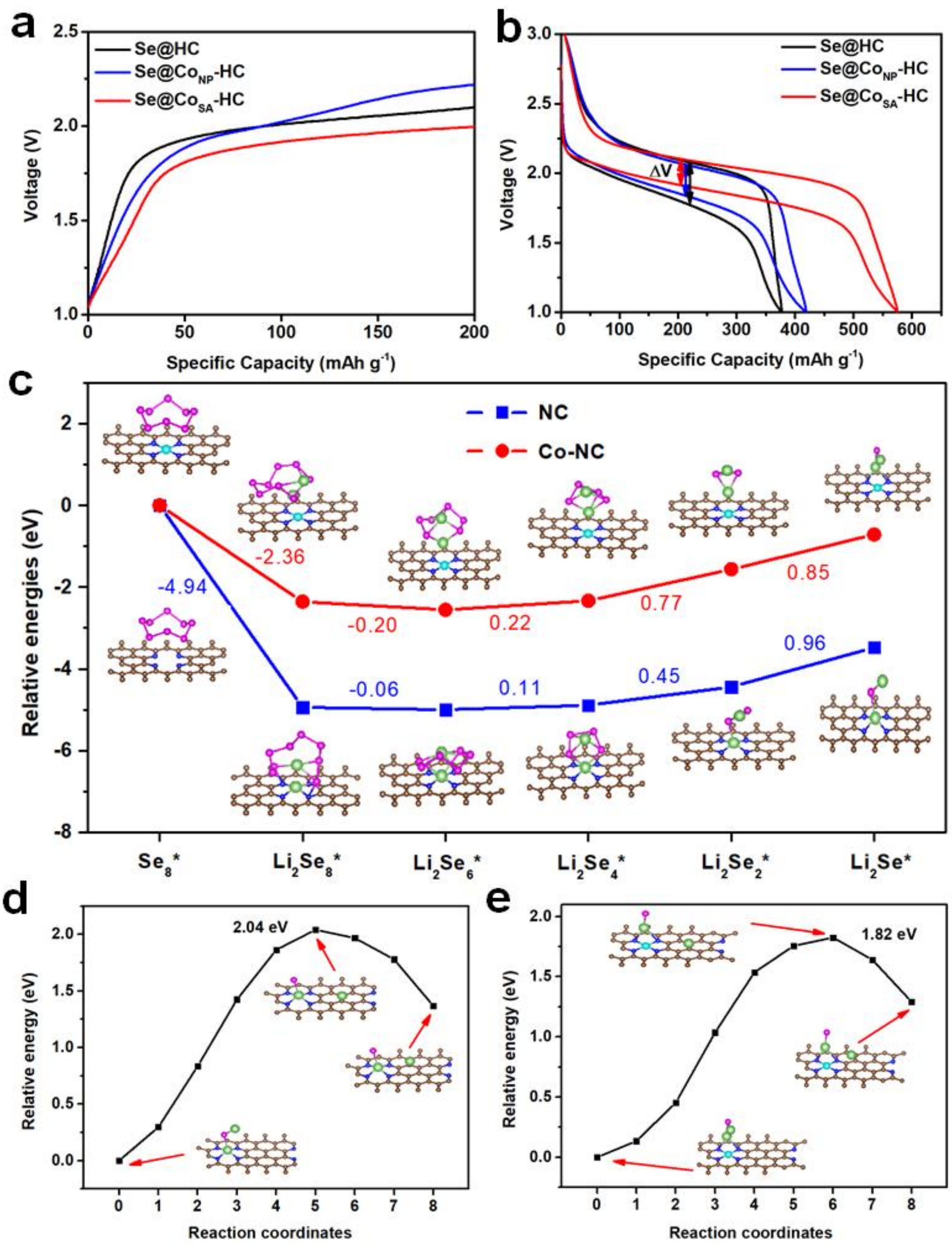

Figure 5. Catalytic effects of Co$_{SA}$-HC particles for Li-Se batteries. (a) The first cycle charge profiles at 0.1C and (b) discharge-charge profile at 0.1C of Se@Co$_{SA}$-HC, Se@HC, and Se@Co$_{NP}$-HC. (c) Energy profiles for the reduction of lithium polyselenides on NC and Co-NC supports (insets: the optimized adsorption conformations of intermediate species on NC and Co-NC substrate). Energy profiles of the transformation of Li_2Se clusters on NC (d) and Co-NC (e). (The insets are: the initial, transition, and final structures, respectively.) The brown, pink, green, blue, and cyan balls represent C, Se, Li, N, and Co atoms, respectively.

Table R1 (Table S5). DFT calculations of the configurations and Li-Se bond length (Å) of lithium polyselenides on NC and Co-NC supports. The brown, pink, green, blue, and cyan balls represent C, Se, Li, N, and Co atoms, respectively.

NC	Configurations and Li-Se bond length (Å)	Co-NC	Configurations and Li-Se bond length (Å)
	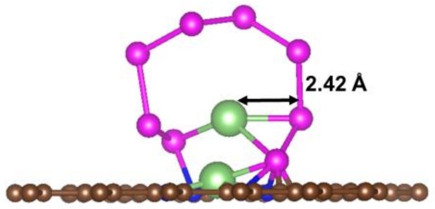 Li_2Se_8		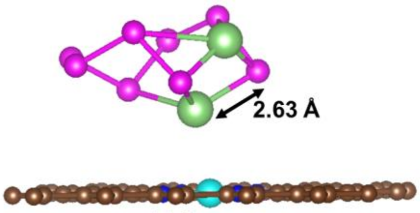 Li_2Se_8
	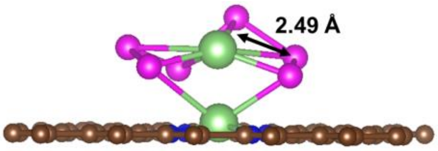 Li_2Se_6		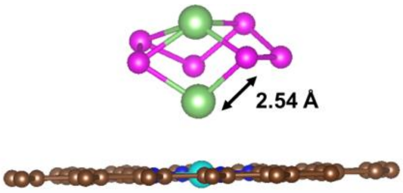 Li_2Se_6
	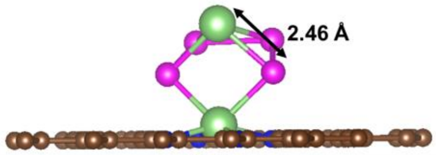 Li_2Se_4		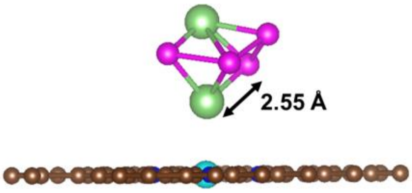 Li_2Se_4
	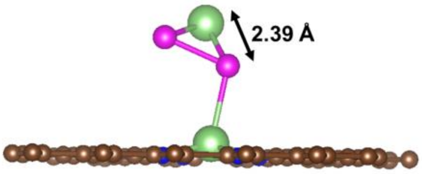 Li_2Se_2		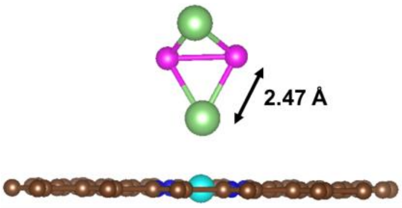 Li_2Se_2
	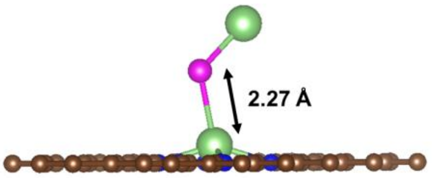 Li_2Se		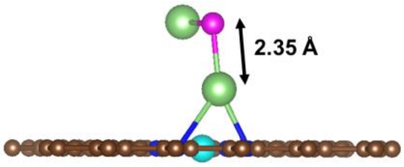 Li_2Se

b) Regarding to the reviewer's concern of Li_2Se_2/Li_2Se layer with the relationship with the catalytic sites, we would like to give detailed explanations as below:

The cell with the Se@Co_{SA}-HC cathode after 1700 cycles was dissembled and the retrieved Se@Co_{SA}-HC cathode materials were characterized by TEM. As shown in **Figure R3a**, it is observed that the morphology of the Se@Co_{SA}-HC has been well preserved, indicating the superior stability of the cathode structure. From STEM element mapping images in **Figure R3c** and **R3d**, energy-dispersive X-ray spectroscopy (EDS) images were obtained to identify the elemental distribution of cobalt and selenium, which are homogeneously distributed in the whole carbon framework. As shown in **Figure R3b**, a homogeneous thin layer with a thickness of ～20 nm has been identified on the surface of the electrode after long-term cycling. The TEM characterizations confirmed that the composition of the layer could be Li_2Se_2/Li_2Se, which could enhance the cycling performance of the Li-Se batteries. The TEM observation results are consistent with the report in the previously published literature[3].

The overall reversible reaction for the formation of Li_2Se originating from Se_8 and Li.[1] During discharge, the first step involves the reduction of Se_8 and the generation of Li_2Se_8, followed by further reduction and disproportionation with the formation of three intermediate lithium polyselenides, e.g., Li_2Se_6, Li_2Se_4, and Li_2Se_2, leading to the formation of Li_2Se as the final discharge product. **Figure S26** in the supporting information shows the EIS results collected at different discharge/charge states and the simulated equivalent circuit is also provided as the inset. At the fresh stage and 2.1V discharging stage, the first semicircle and second semicircle could be ascribed to the charge transfer resistance of Se/C electrode and accumulation of an interfacial layer of surface-adsorbed lithium polyselenide on cathode surface, respectively. In the subsequent EIS at lower potential during discharge, the second semicircles are suppressed, implying decreased resistance by forming more conductive Li_2Se_x during the discharge process. The values of simulated R_2 have been listed in **Table S3**, further proving the formation process of Li_2Se_x during the discharge, as well as the mechanism described in the response (a). In addition, it has been reported that the fully discharged Li_2Se layer on the surface of electrode can enhance the ionic conductivity.[3] Therefore, to more accurately describe this point, we have further corrected the layer as "interfacial layer" in the revised manuscript on Page 13.

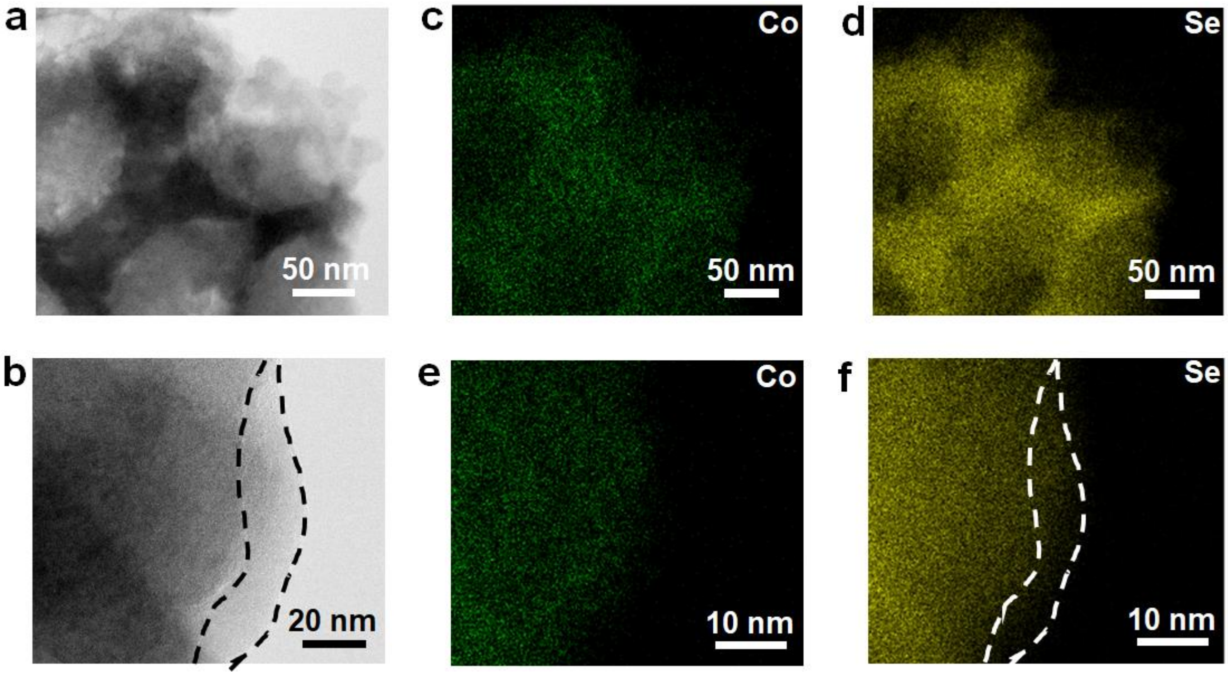

Figure R3 (Figure S28). (a) (b) TEM images and (c) (d) (e) (f) STEM element mapping images of the Se@Co_{SA}-HC cathode after cycling performance at 0.5 C for 1700 cycles.

Figure R3 has been added in the revised supporting information as **Figure S28** on Page 33. The following highlighted and underlined description has been added in the revised manuscript on Page 13 and 14.

The cell with the Se@Co_{SA}-HC cathode after 1700 cycles was dissembled and the retrieved Se@Co_{SA}-HC cathode materials were characterized by TEM. As shown in **Figure S28a**, it is observed that the morphology of the Se@Co_{SA}-HC cathode has been well preserved, indicating the superior stability of the cathode structure. From STEM element mapping images in **Figure S28c** and **S28d**, energy-dispersive X-ray spectroscopy (EDS) images were obtained to identify the elemental distribution of cobalt and selenium, which are homogeneously distributed in the whole carbon framework. As shown in **Figure S28b**, a homogeneous thin layer with a thickness of ~20 nm has been identified on the surface of the electrode after long-term cycling. These characterizations confirmed that the composition of the layer could be Li_2Se_2/Li_2Se, which could enhance the cycling performance of the Li-Se batteries. This result is consistent with the previously reported literature[58].

c) For the reviewer's other concern regarding how the catalytic sites keep active in the long cycles, we would like to give detailed explanation as below:

To confirm the stability of single cobalt atoms after long cycles, the cell after cycling 0.5 C for 1700 cycles was dissembled and the Se@Co_{SA}-HC material after long-term cycling was characterized. From high angle annular dark field scanning transmission electron microscopy (HAADF-STEM) images in **Figure R4**, high-density bright dots (highlighted by red circles) have been detected, which correspond to single atom Co. This unambiguously confirmed that single atom Co kept single atom state and maintained stable in the Se@Co_{SA}-HC cathode after long-term cycling.

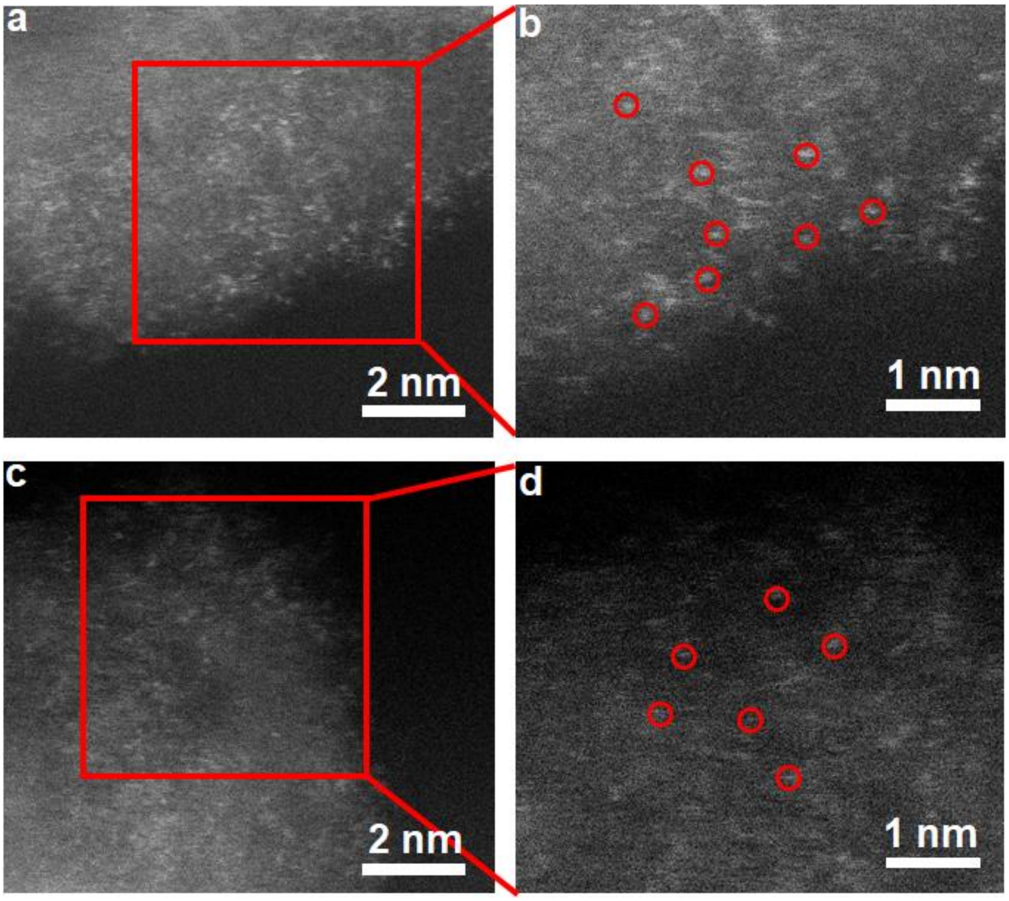

Figure R4 (Figure S35). (a, c) aberration-corrected HAADF-STEM images and (b, d) enlarged images of the Se@Co_{SA}-HC cathode after 1700 cycles.

The above experimental results have been added in the revised Supporting Information.

To further confirm the catalytic activity of single Co atoms after cycling, we conducted a visual observation on the suppression for the formation of lithium polyselenides during the cycling process via single Co atom catalysis. The Se@Co_{SA}-HC cathode after cycled for 1700 cycles and the cycled bare Se@HC cathode after cycled 0.5 C for 100 cycles were used for the visual observation. As shown in **Figure R5a**, H-type cell was used in which the cycled Se@Co_{SA}-HC cathode and bare Se@HC cathode were used as the cathode. The electrolyte in the cell with cycled bare Se@HC cathode (**Figure R5b** and **R5c**) changed from colourless to yellow after the first discharge process, implying that there was a dissolution of polyselenides in electrolyte.[4] It is clear that by using bare Se@HC cathode without single cobalt catalysts, lithium polyselenides were detached from the cathode and dissolved in the electrolyte, indicating that bare Se@HC cathode owns poor immobilization of polyselenides.

In contrast, the colour change of the electrolyte for the cycled Se@Co_{SA}-HC cathode was not observed during three cycles. **Figure R5d** and **R5e** show that the cell cycled Se@Co_{SA}-HC cathode presents transparent electrolyte without colour change, indicating the atomic cobalt electrocatalyst could effectively alleviate the dissolution of polyselenides, maximize polyselenides immobilization, electro-catalyse the transformation from polyselenides to Li_2Se. Therefore, this visual experiment verifies that the single cobalt atoms play an important role that the transformation from polyselenides to Li_2Se have been electro-catalysed, polyselenides immobilization has been maximized and the dissolution of polyselenides has been inhibited. This visual observation using cycled electrodes clearly confirmed that single atom cobalt catalysts remain active after long cycling.

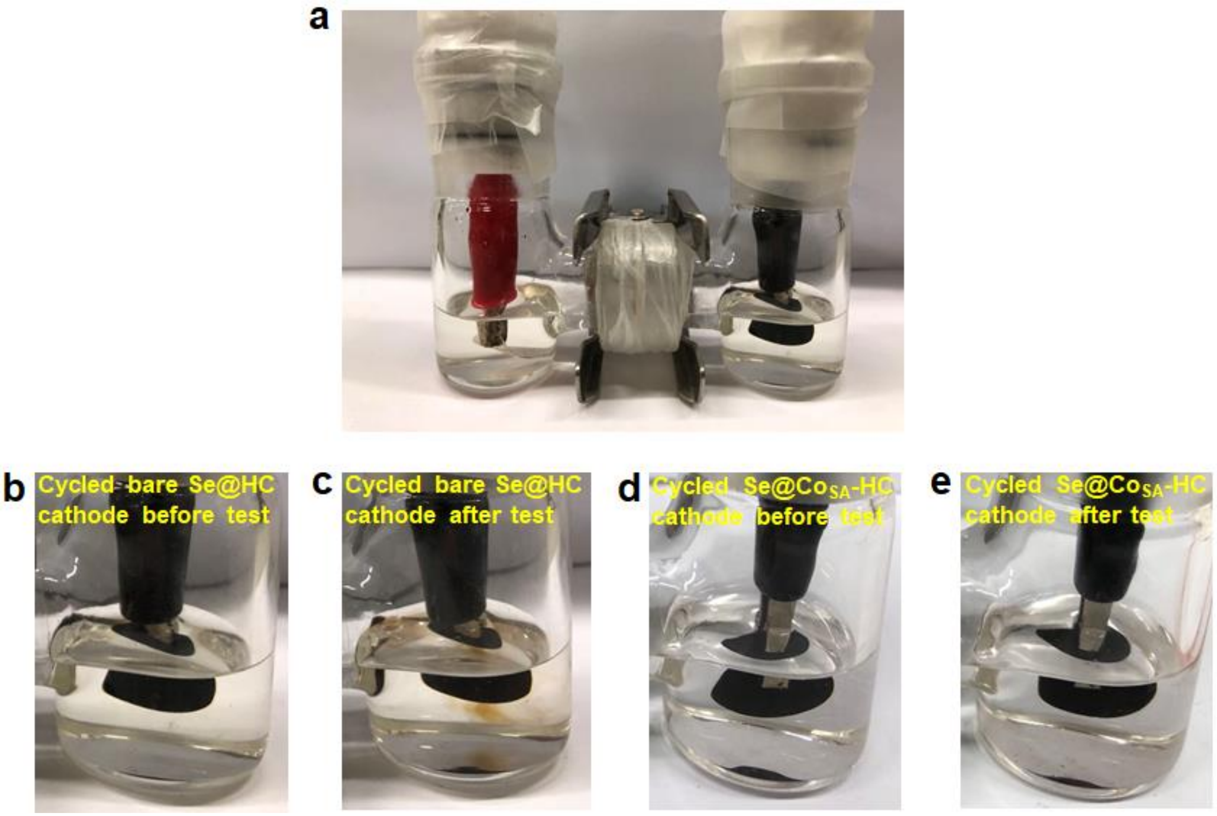

Figure R5 (Figure S36). Visual observation of lithium polyselenide dissolution in the electrolyte before and after cycling (3 cycles) using two types of cycled cathodes for a comparison. (a) H-type cell for Li–Se batteries, (b) cycled bare Se@HC cathode before test, (c) cycled bare Se@HC cathode after test, (d) cycled Se@Co_{SA}-HC cathode (after 1700 cycles) before test, (e) cycled Se@Co_{SA}-HC cathode after test.

The **Figure R3** has been added in the revised supporting information as **Figure S35** on Page 40. The **Figure R4** has been added in the revised supporting information as **Figure S36** on Page 41. The following highlighted and underlined paragraphs have been added in the revised manuscript on Page 14 and 15.

To confirm the catalytic role and stability of single cobalt atoms after long term cycling, the cell after cycled for 1700 cycles was dissembled and the Se@Co_{SA}-HC cathode was retrieved and characterized. From high angle annular dark field scanning transmission electron microscopy (HAADF-STEM) images in **Figure S35**, high-density bright dots (highlighted by red circles) have been clearly detected, revealing that single cobalt atoms in the Se@Co_{SA}-HC cathode are stable during long-term cycling.

To further confirm the catalytic activity of single Co atoms after cycling, we conducted a visual observation on the suppression for the formation of lithium polyselenides during the cycling process via single Co atom catalysis. The Se@Co_{SA}-HC cathode after cycled for 1700 cycles and the cycled bare Se@HC cathode after cycled 0.5 C for 100 cycles were used for the visual observation. As shown in **Figure S36a**, H-type cell was used in which the cycled Se@Co_{SA}-HC cathode and bare Se@HC cathode were used as the cathode. The electrolyte in the cell with cycled bare Se@HC cathode (**Figure S36b** and **S36c**) changed from colourless to yellow after the first discharge process, implying there was a dissolution of polyselenides in electrolyte.[4] It is clear that by using bare Se@HC cathode without single cobalt catalysts, lithium polyselenides were detached from the cathode and dissolved in the electrolyte, indicating that bare Se@HC cathode owns poor immobilization of polyselenides.

In contrast, the colour change of the electrolyte for the cycled Se@Co_{SA}-HC cathode was not observed during three cycles. **Figure S36d** and **S36e** show that the cell cycled Se@Co_{SA}-HC cathode presents transparent electrolyte without colour change, indicating the atomic cobalt electrocatalyst could effectively alleviate the dissolution of polyselenides, maximize polyselenides immobilization, electro-catalyse the transformation from polyselenides to Li_2Se. Therefore, this visual experiment verifies that the single cobalt atoms play an important role that the transformation from polyselenides to Li_2Se have been electro-catalysed, polyselenides immobilization has been maximized and the dissolution of polyselenides has been inhibited. This visual observation using cycled electrodes clearly confirmed that single atom cobalt catalysts remain active after long cycling.

References:

1. Luo, C., et al. Selenium@mesoporous carbon composite with superior lithium and sodium storage capacity. ACS Nano 7, 8003-8010 (2013).
2. Li, Z., et al. Confined selenium within porous carbon nanospheres as cathode for advanced Li–Se batteries. Nano Energy 9, 229-236 (2014).
3. Liu, F., *et al.* A Mixed Lithium-Ion Conductive Li_2S/Li_2Se Protection Layer for Stable Lithium Metal Anode. Adv. Funct. Mater. 30, 2001607 (2020).
4. Gu, X., *et al.* Highly Reversible Li–Se Batteries with Ultra-Lightweight N, S-Codoped Graphene Blocking Layer. Nano-Micro Lett. 10, 59 (2018)

Reviewer #2 (Remarks to the Author):

The manuscript was revised carefully, I recommend acceptation of this revised manuscript.

Responses to Reviewer' Comments

Responses to Reviewer #2

Reviewer Letter:

The manuscript was revised carefully, I recommend acceptation of this revised manuscript.

Response:

We appreciate the reviewer's positive comments on our work. We sincerely thank the reviewer once again for the time and efforts to improve the quality of our work.
